# Genome-wide identification and expression profiling of *Alba* gene family members in response to abiotic stress in tomato (*Solanum lycopersicum* L.)

**DOI:** 10.1186/s12870-021-03310-0

**Published:** 2021-11-12

**Authors:** Antt Htet Wai, Lae-Hyeon Cho, Xin Peng, Muhammad Waseem, Do-jin Lee, Je-Min Lee, Chang-Kil Kim, Mi-Young Chung

**Affiliations:** 1 Department of Agricultural Education, Sunchon National University, 413 Jungangno, Suncheon, Jeonnam 540-950 Republic of Korea; 2 Department of Biology, Yangon University of Education, Kamayut Township, Yangon Region 11041 Myanmar; 3 Department of Plant Bioscience, College of Natural Resources and Life Science, Pusan National University, Miryang-si, Gyeongsangnam-do 50463 Republic of Korea; 4 Institution of Genomics and Bioinformatics, South China Agricultural University, Guangzhou, China; 5 College of horticulture, South China Agricultural University, Guangzhou, China; 6 Department of Horticulture, Kyungpook National University, Daegu, Republic of Korea

**Keywords:** *Alba* genes, *Solanum lycopersicum*, Abiotic stress, Expression profiling, Subcellular localization, 3D structure, Gene co-expression network

## Abstract

**Background:**

Alba (Acetylation lowers binding affinity) proteins are an ancient family of nucleic acid-binding proteins that function in gene regulation, RNA metabolism, mRNA translatability, developmental processes, and stress adaptation. However, comprehensive bioinformatics analysis on the *Alba* gene family of *Solanum lycopersicum* has not been reported previously.

**Results:**

In the present study, we undertook the first comprehensive genome-wide characterization of the *Alba* gene family in tomato (*Solanum lycopersicum* L.). We identified eight tomato *Alba* genes, which were classified into two groups: genes containing a single Alba domain and genes with a generic Alba domain and RGG/RG repeat motifs. *Cis*-regulatory elements and target sites for miRNAs, which function in plant development and stress responses, were prevalent in *SlAlba* genes. To explore the structure-function relationships of tomato Alba proteins, we predicted their 3D structures, highlighting their likely interactions with several putative ligands. Confocal microscopy revealed that SlAlba–GFP fusion proteins were localized to the nucleus and cytoplasm, consistent with putative roles in various signalling cascades. Expression profiling revealed the differential expression patterns of most *SlAlba* genes across diverse organs. *SlAlba1* and *SlAlba2* were predominantly expressed in flowers, whereas *SlAlba5* expression peaked in 1 cm-diameter fruits. The *SlAlba* genes were differentially expressed (up- or downregulated) in response to different abiotic stresses. All but one of these genes were induced by abscisic acid treatment, pointing to their possible regulatory roles in stress tolerance via an abscisic acid-dependent pathway. Furthermore, co-expression of *SlAlba* genes with multiple genes related to several metabolic pathways spotlighted their crucial roles in various biological processes and signalling.

**Conclusions:**

Our characterization of *SlAlba* genes should facilitate the discovery of additional genes associated with organ and fruit development as well as abiotic stress adaptation in tomato.

**Supplementary Information:**

The online version contains supplementary material available at 10.1186/s12870-021-03310-0.

## Background

Alba (Acetylation lowers binding affinity) superfamily proteins belong to an ancient group of nucleic acid-binding proteins that originated prior to the divergence of archaea and eukaryotes and are widely distributed in nearly all kingdoms of life [1, 2]. These small, basic proteins interact with DNA and RNA as homodimers or heterodimers [3–6] and contain a conserved nucleic acid-binding Alba domain with an IF3-C fold [1]. Alba proteins also show the distinct characteristic of being regulated via acetylation by PAT (protein acetyl transferase) and deacetylation by Sir2 (an NAD^+^-dependent histone deacetylase, HDAC), indicating that they function as transcriptional regulators similar to histones [7–11]. Although Alba proteins were initially classified as chromosomal proteins and were considered to function in the maintenance of chromatin architecture and transcriptional repression, many studies have documented their functional diversity, including their roles in genome packaging and organization, transcriptional and translational regulation, RNA metabolism, and development and differentiation processes [2, 12–14].

Alba proteins exist in two forms: relatively small proteins with a single generic Alba domain; and larger proteins with an Alba domain plus RGG/RG repeat motifs or additional domain/s [2, 15]. All archaeal Alba homologs, which belong to the Archaea Alba protein family, are relatively small proteins with only a single Alba domain. Eukaryotic Alba proteins diverged into two paralogous lineages, the Rpp20-like family (with single Alba domains) and the Rpp25-like family (mostly larger proteins with RGG/RG repeat motifs or additional domains) [1]. The RGG/RG repeat motifs are thought to have roles in DNA damage signalling, snRNP biogenesis, the regulation of apoptosis, transcription, pre-mRNA splicing, and translation, many of which are at least partly controlled by the arginine methylation of RGG/RG repeats [16].

*Alba* genes encoding proteins with RGG/RG repeat motifs have been identified in many species [15, 17, 18]. Various types of domains with diverse biological functions have also been identified in combination with the Alba domain in numerous Alba family proteins in many domains of life, such as the F-box domain in the fungus *Taphrina*; the CLIP1 zinc finger domain in the nematode *Pristionchus*; the ATP synthase subunit H domain in the protozoan parasite *Theileria*; the sugar transporter domain in the Florida lancelet; the DEAD/DEAH box helicase domain in plants; and so forth [2]. The addition of RGG/RG repeat motifs and new domains to genes with a single Alba domain appears to have contributed to speciation and the functional diversification of Alba family proteins to meet the demands of increasing cellular variety and complexity.

Diverse abiotic stresses, including drought, salinity, heat, and cold stress, can hamper plant growth and development, resulting in significantly reduced crop productivity [19]. Various environmental stresses trigger changes in *Alba* behaviour. For example, LiAlba1 and LiAlba3, from the protozoan parasite *Leishmania infantum*, repress translation by interacting with RNA-binding proteins and ribosomal subunits. These proteins also function as translation factors and translocate from the cytoplasm into the nucleolus and flagellum in response to heat stress [20]. Similarly, all four Alba proteins of *Trypanosoma brucei* (TbAlba1, TbAlba2, TbAlba3, and TbAlba4) are RNA-binding proteins that localize to the cytoplasm as parts of stress granules (SG) upon exposure to nutrient stress [21]. The stress-induced differential expression has also been observed for *Alba* genes from rice treated with different abiotic stresses and phytohormones [22]. Mild heat stress (37 °C) altered the expression of most Arabidopsis *Alba* genes in inflorescences [15]. In cotton (*Gossypium hirsutum*), *GhALBA4* and *GhALBA5* were significantly induced by water deficit and salinity treatment, and plants in which the expression of these genes was repressed by virus-induced gene silencing (VIGS) were highly sensitive to dehydration as well as salt stress, pointing to their putative roles in abiotic stress tolerance [23].

Genome-wide identification of *Alba* genes has been performed in several plant species, but no comprehensive study of the evolutionary relationships or characteristics of the *Alba* gene family in solanaceous crops has been reported. Tomato (*Solanum lycopersicum* L.) is an economically important fruit whose yields are severely affected by unfavorable environmental conditions [19]. The molecular mechanisms regulating fruit development and ripening have been extensively studied in this model fruit crop with the aim of increasing fruit yield under diverse environmental stresses. Hence, in the current study, we performed a comprehensive genome-wide analysis, gene co-expression network analysis and expression profiling of *SlAlba* genes in response to various abiotic stresses. Our findings provide a basis for further functional characterization of *Alba* genes involved in the development and stress tolerance of tomato.

## Results

### Identification and sequence analysis of Alba family proteins in tomato

We identified eight non-redundant putative *Alba* genes in tomato, which were named *SlAlba1* to *SlAlba8* in accordance with their positions on the chromosomes. The open reading frames of the *SlAlba* genes showed considerable variation in length, ranging from 384 bp to 1188 bp for *SlAlba5* and *SlAlba7*, respectively, with a mean of 701 bp. The predicted sizes of the eight SlAlba proteins varied from 127 (*SlAlba5*) to 395 (*SlAlba7*) amino acids (aa), with a mean of 236 aa. The computed molecular weights of these proteins ranged from 14.24 to 43.29 with iso-electric points (pI) of 5.61 to 10.22. Based on their predicted pI values, the SlAlbas include both acidic and basic proteins. The grand average of hydropathicity (GRAVY) values of these proteins ranged from − 1.126 to − 0.312, indicating that they all are hydrophilic proteins (Table 1).

**Table 1:** Characteristics of the *Alba* genes and corresponding proteins in tomato genome

Gene Name	Locus name	ORF (bp)	Chromosome No.	Protein					Subcellular Localization	No. of Introns
				Length (aa)	Alba Domain Start-End (aa)	MW (kDa)	PI	GRAVY		
*SlAlba1*	Solyc01g097050	498	1	193	67-130	21.20	9.7	−0.312	Chloroplast	3
*SlAlba2*	Solyc04g071690	702	4	233	19-85	25.17	9.86	−0.709	Cytoplasm	5
*SlAlba3*	Solyc04g081880	765	4	254	19-84	28.01	10.22	−0.965	Cytoplasm	6
*SlAlba4*	Solyc06g065980	387	6	128	20-84	14.41	5.61	−0.548	Cytoplasm	4
*SlAlba5*	Solyc06g068050	384	6	127	20-85	14.24	6.32	−0.373	Chloroplast	4
*SlAlba6*	Solyc06g083540	789	6	262	19-82	29.23	9.95	−1.126	Cytoplasm	6
*SlAlba7*	Solyc09g061710	1188	9	395	19-84	43.29	9.28	−0.826	Cytoplasm	8
*SlAlba8*	Solyc09g091590	897	9	303	24-89	32.90	9.68	−1.032	Nucleus	7

*ORF* Open reading frame, *bp* Base pair, *aa* Amino acid, *kDa* Kilo Dalton, *pI* Isoelectric point, *MW* Molecular weight, *GRAVY* Grand average of hydropathicity

### Phylogenetic and domain analysis of tomato Alba proteins

**Fig. 1:**
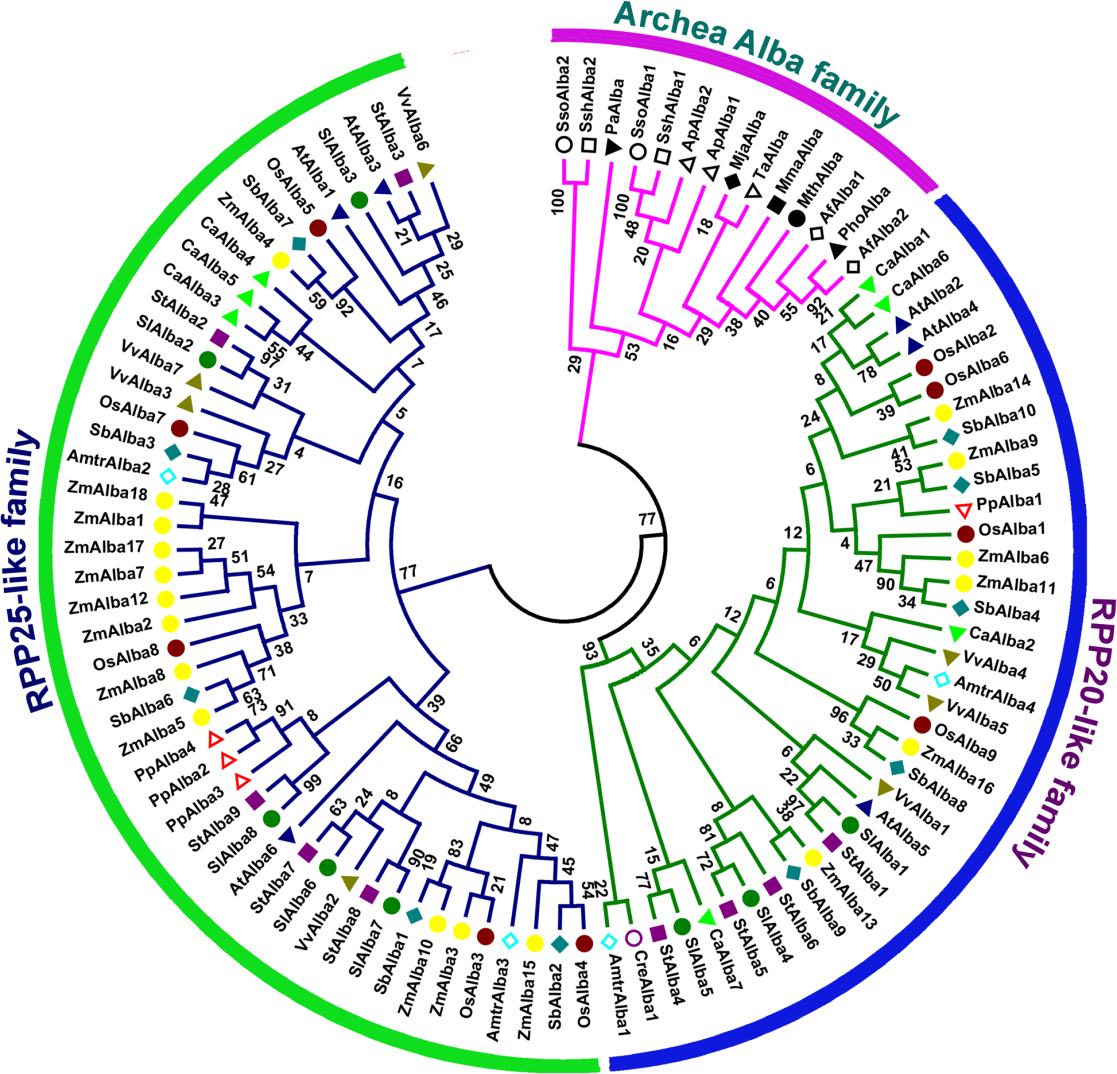
Phylogenetic relationship of Alba proteins from tomato and different organisms. The phylogenetic tree was constructed by the neighbor-joining method in MEGA6 software with 1000 bootstrap replicates using full-length Alba protein sequences. The Alba family proteins were clustered into three distinct families: archaeal Alba family, RPP20-like family and RPP25-like family. A species acronym was added before each Alba protein name: Sl, *Solanum lycopersicum*; St, *Solanum tuberosum;* At, *Arabidopsis thaliana;* Vv, *Vitis vinifera*; Ca, *Cicer arietinum;* Os, *Oryza sativa*; Zm, *Zea mays*; Sb, *Sorghum bicolor*; Amtr, *Amborella trichopoda;* Pp, *Physcomitrella patens; Cre, Chlamydomonas reinhardtii; Sso, Sulfolobus solfataricus; Ssh, S. shibitae; Ap, Aeropyrum pernix; Pho, Pyrococcus horikoshii; Mth, Methanobacterium thermoautotrophicum; Mja, Methanococcus jannaschii; Mma, Methanococcus maripaludis; Af, Archaeoglobus fulgidus; Pa, Pyrobaculum aerophilum and Ta, Thermoplasma acidophilum*

Phylogenetic analysis clearly placed Alba family members from multiple diverse species into three major families: one archaea-specific family and two eukaryote-specific families, the RPP20-like and RPP25-like families (Fig. 1). The archaea-specific family solely comprises archaeal Alba proteins, whereas the eukaryotic homologs of Alba are organized into two distinct families. We constructed an evolutionary tree, which revealed a major cluster containing proteins from dicots and monocots together with lower plants. The tomato Alba proteins were distributed in both eukaryote-specific families, with the largest number of SlAlbas being RPP25-like family proteins with RGG/RG repeat boxes. The remaining SlAlbas lacked RGG/RG motifs and were grouped in the RPP20-like family; no SlAlba protein was found in the archaea-specific family. Phylogenetic tree analysis indicated that the Alba members from tomato were selectively paired with those from the closely related solanaceous crop potato in both eukaryotic families.

Interestingly, the RPP20-like Alba family members from the basal angiosperm *Amborella* showed a close evolutionary relationship with the corresponding proteins from the single-celled green alga *Chlamydomonas reinhardtii* and dicotyledonous grapevine, whereas the RPP25-like proteins of *Amborella* were grouped in the same clades as those of monocotyledonous crop species. Alba homologs from the moss *Physcomitrella* (PpAlb2, PpAlb3, and PpAlba4) were clustered together and showed closer relationships with those of solanaceous crops in the RPP25-like family, whereas the RPP20-like protein (PpAlb1) was clustered together with those of cereal crops. In the archaeal Alba protein group, SsoAlba1 and SsoAlba2 from *Sulfolobus solfataricus* were paired with their related orthologs SshAlba1 and SshAlba2, respectively, from *Sulfolobus shibitae.* However, *Archaeoglobus fulgidus* (AfAlba1) and *Aeropyrum pernix* (ApAlba1) were grouped in the same clades with their paralogs derived from lineage-specific evolution (AfAlba2 and ApAlba2). These results point to the diversification of Alba proteins across various kingdoms of life as well as the sequence conservation in related groupings during the evolutionary process.

**Fig. 2:**
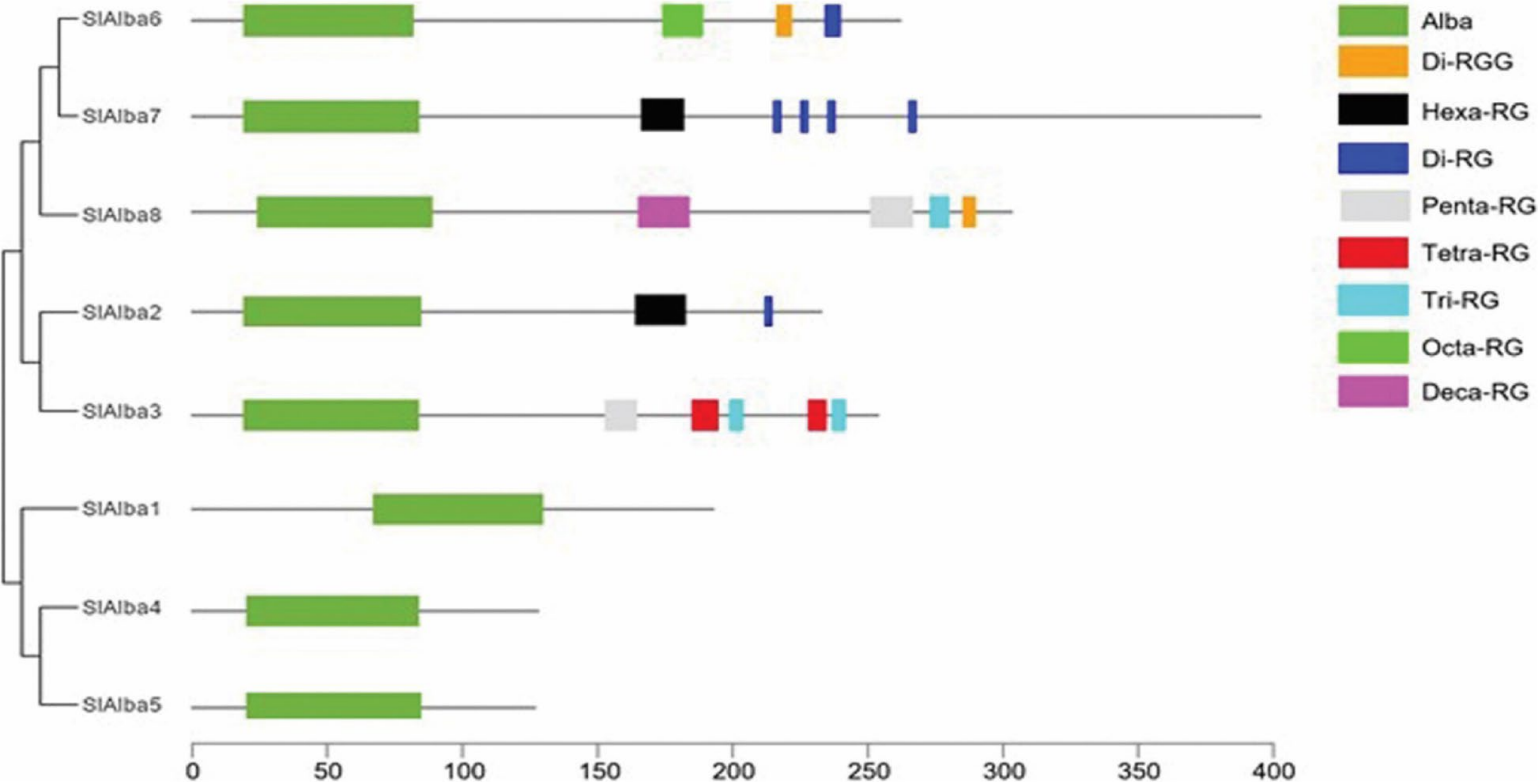
Schematic representation of the Alba domain and RGG/RG repeat motifs identified in SlAlba proteins. RGG/RG repeat motifs are defined in accordance with Thandapani et al. [16] as follows: the di-RGG motif consists of two repeated RGG sequences spaced 0–4 residues apart, denoted as RGG(X0–4)RGG; the deca-, octa-, hexa-, penta-, tetra-, tri-, and di-RG motifs comprise ten, eight, six, five, four, three, and two repeated RG sequences, respectively, 0–4 residues apart

Of the eight Alba proteins identified in tomato, five (SlAlba2, SlAlba3, SlAlba6, SlAlba7, and SlAlba8) displayed a long-form structure comprising an N-terminal Alba domain followed by a C-terminal region with several arginine-glycine (RGG) repeats, which function as RNA recognition motifs to interact particularly with guanine (G)-quadruplexes in RNA [24, 25]. However, these motifs were absent from the three remaining, short-form SlAlba proteins (SlAlba1, SlAlba4, and SlAlba5) (Fig. 2). The clustering of the long-form SlAlba proteins and short-form SlAlba proteins in different eukaryotic families highlights the importance of RGG repeats in the structural and functional diversification of tomato Alba proteins during the course of evolution.

**Fig. 3:**
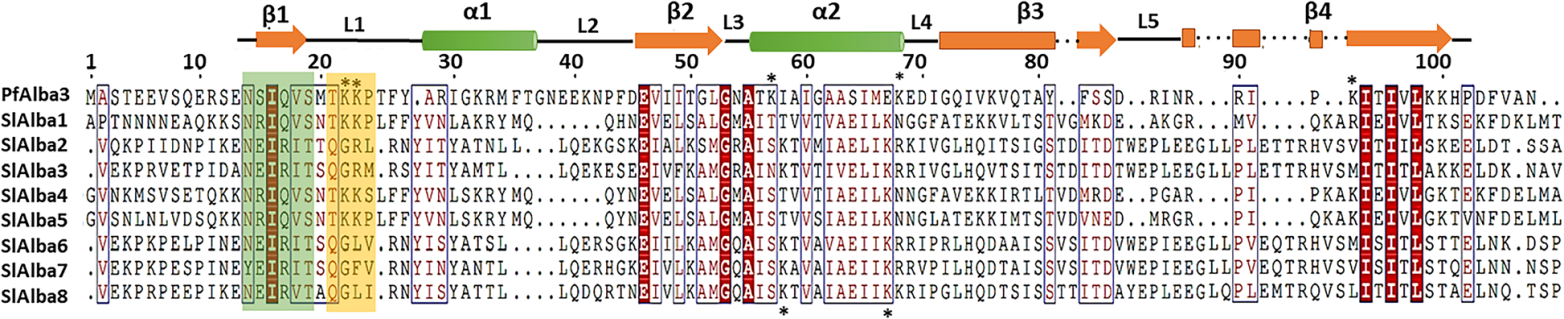
Multiple sequence alignment of the tomato Alba proteins. The protein sequences of the SlAlbas were aligned with that of the homolog from the human malaria parasite using Clustal Omega. The secondary structural elements defined by the ESPript 3.0 web server are also displayed above the alignment. The hexapeptide motifs at the beginning of the Alba domains and the tetrapeptide motifs in the first loop, which are critical for DNA binding, are marked by green and orange boxes, respectively. Asterisks indicate surface-exposed lysines engaged in DNA binding

All Alba proteins from tomato and potato belonging to the RPP20-like family share a conserved NRIQVS-hexapeptide motif at the start of their Alba domains, whereas most of their homologs from the RPP25-like family contain a distinct conserved NEIRIT-hexapeptide motif (Fig. 3). These findings indicate that these motifs were conserved in tomato and potato and are related to the structural and functional similarity of Alba family members from solanaceous crops. Multiple sequence alignment of SlAlba proteins with a well-characterized Alba homolog from the human malaria parasite *Plasmodium falciparum* highlighted the evolutionary conservation of RPP-20-like family Alba proteins from a unicellular parasite and a flowering plant, particularly the presence of similar amino acid residues in the hexapeptide motif at the start of the Alba domain and in the TKKP-tetrapeptide motif of the first loop (L1), which are related to the DNA binding affinity of the Alba domain (Fig. 3).

### Analysis of the exon-intron structures and conserved motifs of *SlAlba* genes

Analysis of the exon-intron structures of the *SlAlba* genes showed that the numbers of exons (4 or 5) in tomato *Alba* genes in the RPP20-like family were almost identical. By contrast, members of the RPP25-like family contained 6 to 9 exons, with an average of 7 (Fig. S1).

*SlAlba7* had the most exons (9), while *SlAlba1* had the fewest (4). However, the genes in the same phylogenetic group shared similar exon-intron compositions in terms of intron number and exon size.

In-depth motif analysis revealed that Alba proteins from the RPP20-like family had fewer motifs (3 or 4) than those from the RPP25-like family (7 to 14 with a mean of 9) (Figs. S2 and S7). Motifs 1 and 2 in the Alba domain were conserved in all Alba proteins, while motifs 7, 8, and 13, which contain the RGG/RG repeat motif, were prevalent only in large Alba proteins belonging to the RPP25-like family. All RPP20-like family members shared similar motif structures and compositions, containing motif 3, which is characteristic of the members of the RPP20-like family, in addition to motifs 1 and 2. These findings highlight the importance of these motifs in the evolutionary conservation of Alba proteins from this family in both monocot and dicot plants. Motifs 1, 2, 4, 5, and 6 were highly conserved in all members of the RPP25-like family, pointing to their role in the structural similarity among Alba members belonging to this group in dicots and monocots. Several conserved motifs were unique to certain RPP25-like family members, such as motif 9 for SlAlba7, motif 11 for SlAlba1 and OSAlba7, motif 12 for SlAlba6 and SlAlba7, motif 14 for SlAlba8 and OsAlba7, and motif 15 for OsAlba5 and OsAlba7. Interestingly, motif 10 was absent from Arabidopsis but present in many rice and tomato family members. Among the conserved motifs identified in Alba proteins, motif7, motif8, and motif13, all of which are composed of RG rich repeats, play a role in the interaction with nucleic acids and proteins [16].

### Chromosomal distribution, gene duplication, and microsynteny analysis

The eight tomato *Alba* genes were distributed on four of the 12 tomato chromosomes (1, 4, 6, and 9), with all genes residing close to the distal ends of the chromosomes. The *SlAlba* genes appeared to be distributed unevenly among the chromosomes, with a single gene on chromosome 1, two genes each on chromosomes 4 and 9, and three genes on chromosome 6 (Fig. S3).

**Fig. 4:**
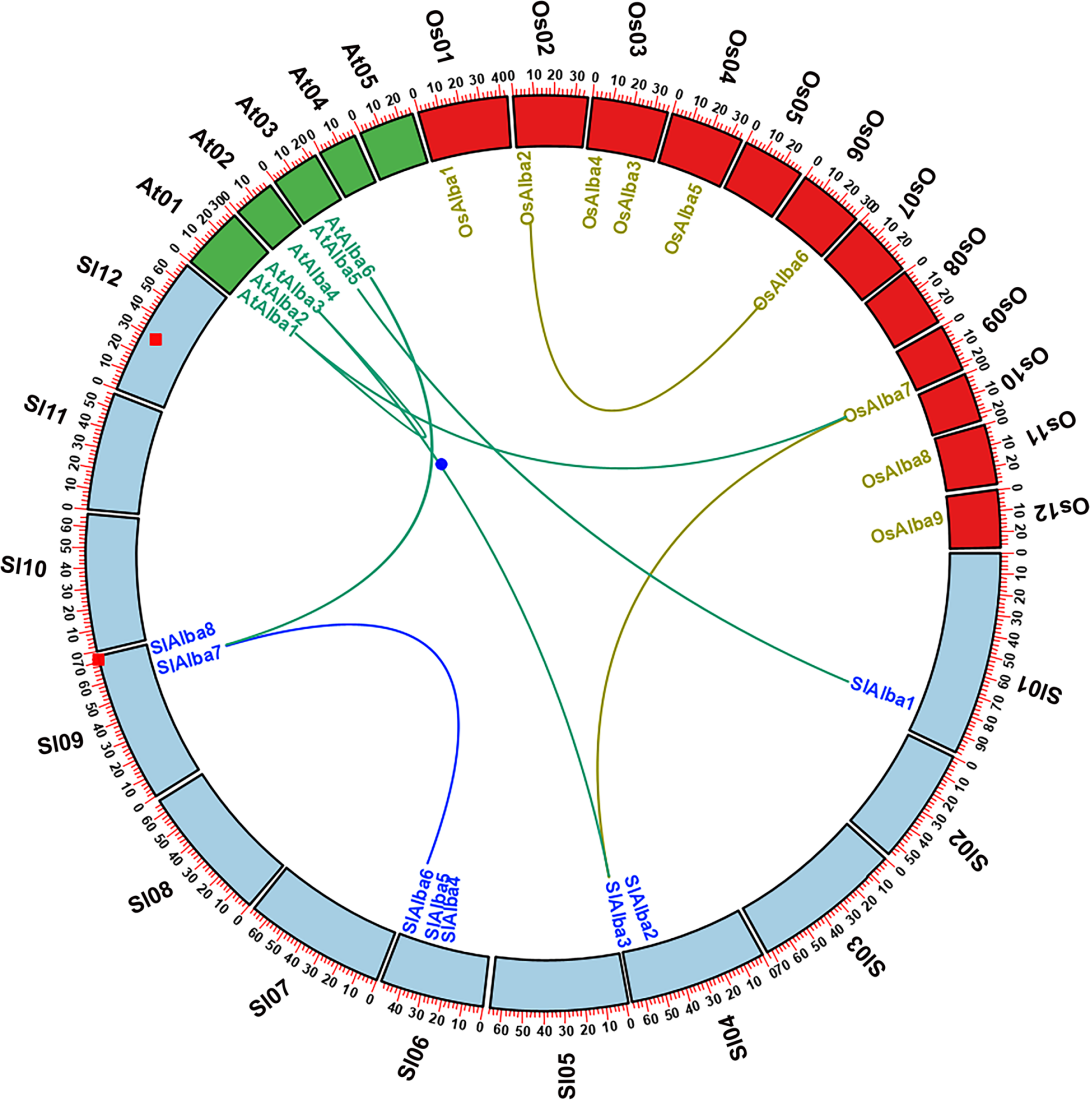
Microsyntenic relationship of *Alba* genes among tomato, Arabidopsis, and rice. The chromosomes of the three species are indicated by different colours: tomato, aqua; Arabidopsis, lime; and rice, red. All chromosomes are depicted to scale in megabase pairs (Mbp). The duplicated *SlAlba* genes in tomato chromosomes are connected by a blue line

Of the eight *SlAlba* genes, only *SlAlba6* and *SlAlba7* were predicted to be a segmentally duplicated gene pair; these genes are located on chromosomes 6 and 9, respectively (Fig. S4). No tandemly duplicated genes were predicted in the *SlAlba* gene family since no genes resided within a 100-kb distance on the same chromosome. SlAlba proteins from the same phylogenetic clades exhibited higher sequence identity compared to those from different clades (Table S4). The Ka/Ks ratio of the duplicated gene pair (*SlAlba6*/*SlAlba7*) was > 1, indicating that these genes had undergone positive selection during the process of evolution. The predicted divergence time of the paralogous gene pair (*SlAlba6*/*SlAlba7*) indicates that the gene duplication event took place 64.09 million years ago (MYA) (Table 2). We constructed a comparative microsynteny map to analyze orthologous *Alba* gene pairs from tomato, rice, and Arabidopsis in order to investigate the evolutionary history and relationships across their genomes. This analysis predicted three orthologous gene pairs between tomato and Arabidopsis, but only one orthologous gene pair between tomato and rice, as well as between Arabidopsis and rice (Fig. 4).

**Table 2:** Estimated Ka/Ks ratio of the duplicated *SlAlba* gene pair with its divergence time in tomato

Duplicated gene pair	Ka	Ks	Ka/Ks	Duplication type	Types of selection	Time (MYA)
*SlAlba6* vs. *SlAlba7*	2.188636	1.922748	1.138286	Segmental	Positive selection	64.09

*Ks* The number of synonymous substitutions per synonymous site, *Ka* The number of non-synonymous substitutions per nonsynonymous site, *MYA* Million years ago

### Analysis of putative stress- and hormone-responsive *cis*-elements and miRNA target sites in *SlAlba* genes

To assess the transcriptional regulation of *SlAlba* genes under abiotic stress conditions, we analyzed the 1500-bp sequences in their promoter regions. We identified various numbers of phytohormone- and stress-responsive *cis*-regulatory elements in the promoters of *SlAlba* genes, including the following: TC-rich repeats (implicated in defence and stress responses); drought-responsive MYB-binding site (MBS); cold- and hypersalinity-responsive low temperature-responsive elements (LTRs); the CGTCA-motif (associated with the jasmonic acid [JA] response); ABRE elements (related to the ABA response); gibberellic acid (GA) response-related TATC-elements and GARG-motifs; TCA-elements (involved in the SA response); WUN motifs (with roles in wounding responses); and TGA elements and AuxRR-core (involved in the auxin response; Fig. S5 and Table S5). We also identified the target sites for several different types of miRNAs involved in various biological processes in the *SlAlba* genes (Table S6), many of which are related to development and stress responses, such as *sly-miR9477-3p*, *sly-miR5303*, *sly-miR9474-5p*, *sly-miR9471a-3p*, *sly-miR9473-3p*, *miR156*, *miR167*, *miR169*, *miR171*, *miR172*, *miR393*, *miR395*, *miR396*, *miR397*, *miR399*, and *miR408*, and *miR827* [26–29].

### Prediction of the three-dimensional structures and functions of SlAlba proteins

**Fig. 5:**
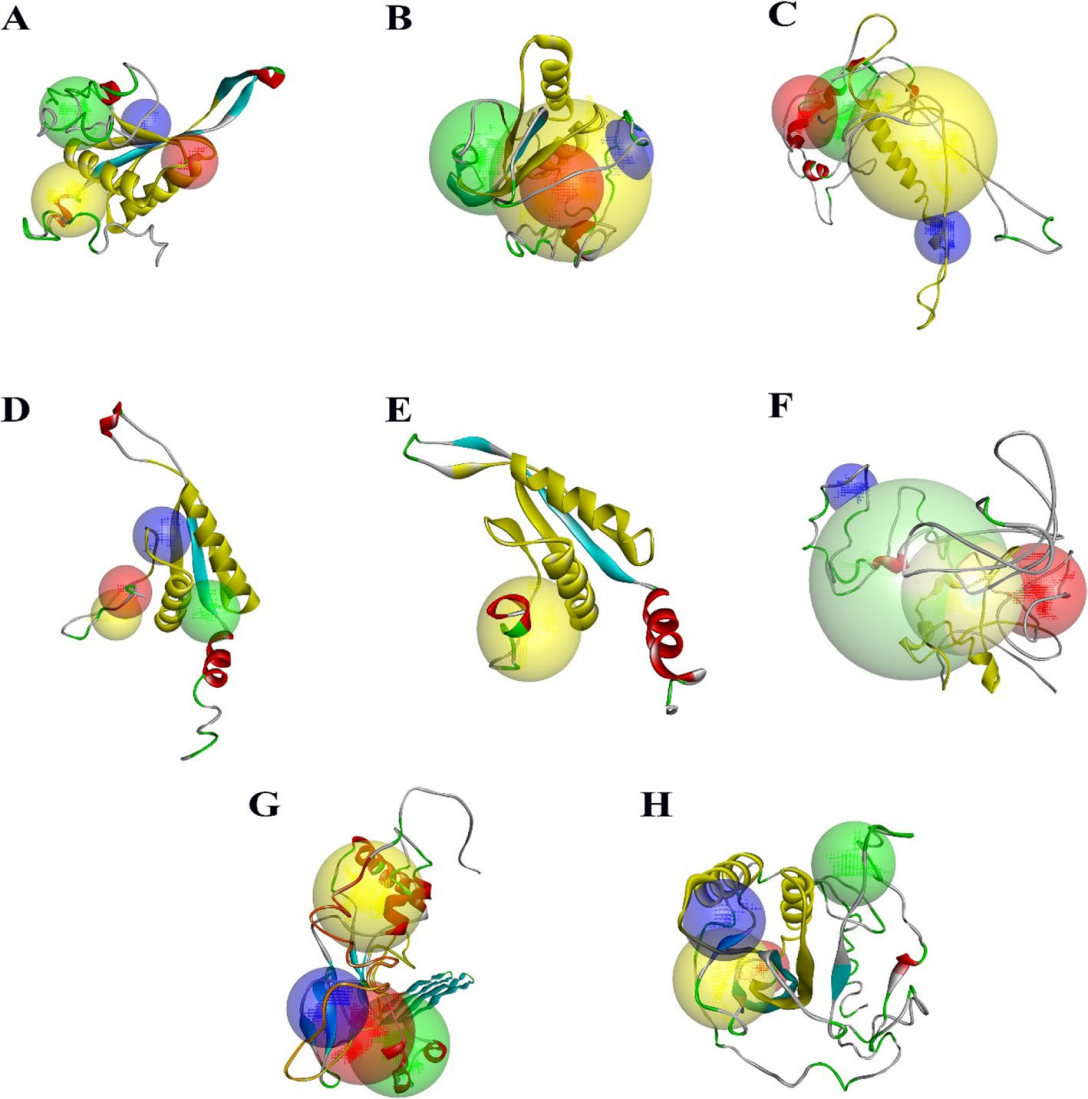
The final 3-dimensional model structures of SlAlba proteins generated by Discovery Studio v.21.1.The secondary structural elements: α-helices (red), β-sheets (cyan), coils (green), and loops (gray) as well as the top four predicted binding sites: site 1 (yellow sphere), site 2 (green sphere), site 3 (red sphere), and site 4 (blue sphere) are depicted for the generated 3D model structures of **A** SlAlba1; **B** SlAlba2; **C** SlAlba3; **D** SlAlba4; **E** SlAlba5; **F** SlAlba6; **G** SlAlba7; and **H** SlAlba8. Alba domain regions in the predicted models are marked with yellow colour

We predicted the 3D structures of SlAlba proteins using the optimal templates listed in Table S7 and other relevant information, such as the percentage of sequence identity, coverage, and Z-scores within the reliable range. An analysis of the resulting models with Discovery Studio v.21.1 revealed the putative binding sites, varied numbers of α-helices, β-strands, and coils in the SlAlba proteins, as shown in Table S8 and Fig. 5. TM-scores of < 0.17 and > 0.5 indicate models with random similarity and a high level of homology, respectively. The TM-scores and RMSD values of the models fell within the reliable range, implying that the models were accurate (Table S9). The C-scores of the predicted models ranged from − 4.30 (SlAlba8) to − 0.70 (SlAlba4), and other parameters, such as the number of decoys and cluster density, demonstrated that the results were within a credible range (Table S10).

We investigated the binding residues for the SlAlba proteins based on an alignment between the template and the newly generated models. Five of the eight SlAlba proteins (SlAlba1, SlAlba2, SlAlba4, SlAlba5, and SlAlba8) were predicted to bind to both DNA and RNA in addition to other ligands, such as peptides, arginine, and manganese. SlAlba3 and SlAlba7 displayed no affinity for nucleic acids, but they did have affinity for other ligands, such as β-carotene and magnesium, whereas SlAlba6 exhibited a specific affinity for RNA (Fig. S6). Gene ontology (GO) analysis suggested that the tomato Alba family members play different roles in diverse biological processes, including chromosome condensation, cellular response to stress, and carbohydrate metabolic process, a putative role in cytolysis and protein complex oligomerization, and rRNA methylation (Table S12).

### Expression analysis of *SlAlba* genes in different organs

Expression profiling revealed that most *SlAlba* genes showed differential expression patterns across the various organs investigated. Four out of the eight *Alba* genes were predominantly expressed in stem tissues, while two other *Alba* genes were preferentially expressed in flowers and the remaining two genes showed maximum expression in 1 cm fruits and leaves, respectively.

*SlAlba1* was highly expressed in flowers at various stages of development, such as floral buds (550-fold relative to roots), full blooming flowers (800-fold relative to roots), and senescent flowers (250-fold relative to roots), whereas it displayed minimal expression in roots and 1 cm fruits and no expression in other organs. By contrast, homologs of *SlAlba1* in the same phylogenetic group, *SlAlba4* and *SlAlba5*, were broadly expressed across all organs examined. *SlAlba4* showed the highest expression in stems (3-fold vs. the control [leaves]), but its transcript levels were significantly lower (2-fold to 4-fold) in fruits at all stages of development except for 1 cm fruits compared to the control. *SlAlba5* was 3- to 8.5-fold more highly expressed in fruits at all six developmental stages except immature fruits compared to the control. The expression of *SlAlba5* peaked (8.5-fold vs. the control) in 1 cm fruits, followed by stems (> 5-fold relative to the control).

**Fig. 6:**
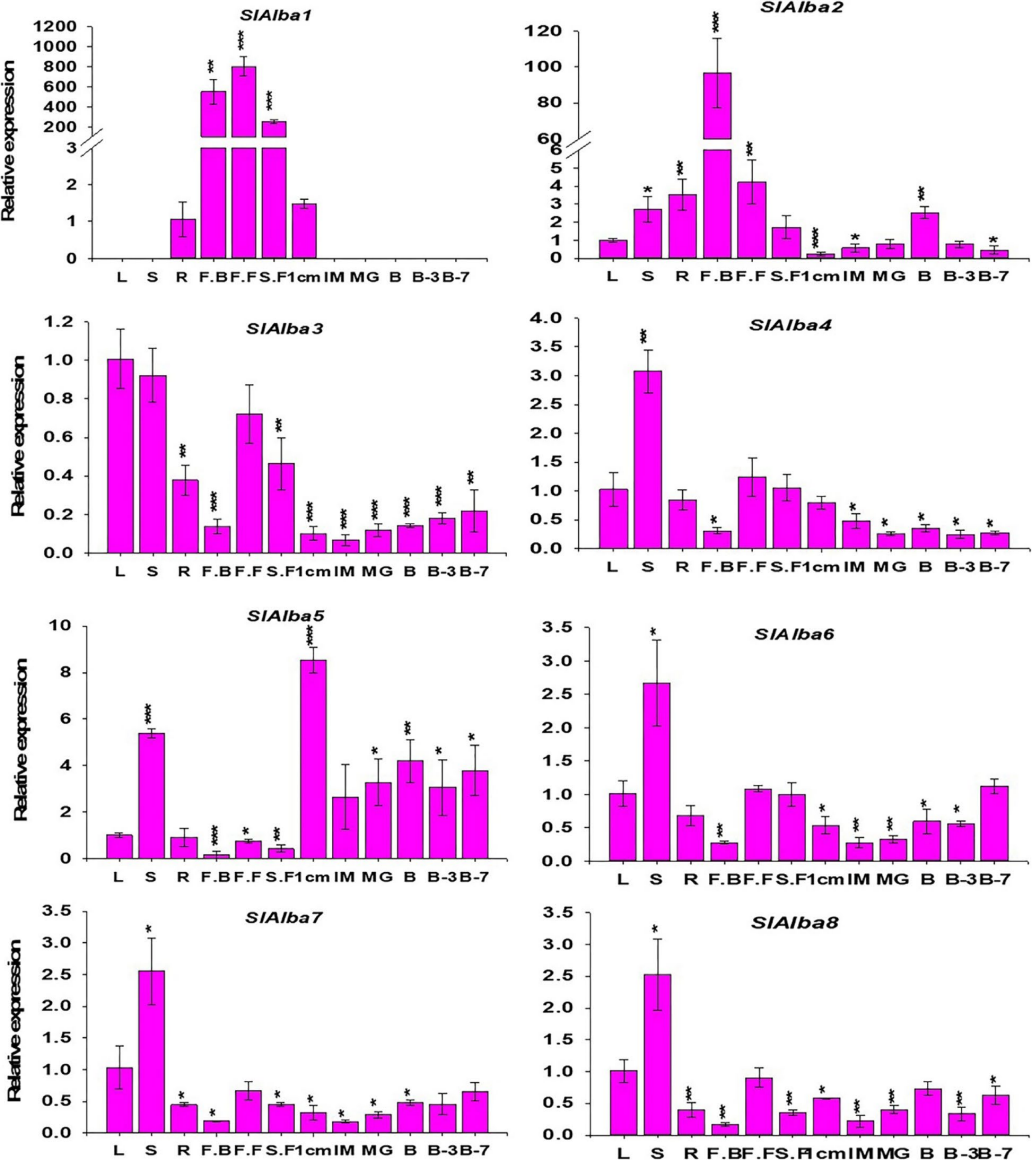
Quantitative reverse-transcription PCR (RT-qPCR) analysis of *SlAlba* genes in 12 organs: leaves, roots, stems, flower buds (FB), full blooming flowers (FF), senescent flowers (SF), 1 cm fruits, immature fruits (IM), mature green fruits (MG), breaker fruits (B), fruits at 3 days after the breaker stage (B3), and fruits at 7 days after the breaker stage (B7). Error bars represent the standard deviations of the means of three independent replicates. *, **, and *** indicate the significant differences between the control samples (roots for *SlAlba1* and leaves for the remaining *SlAlba* genes) and the samples collected from the other organs, as determined by Student’s t-test, at *p*-values ≤0.05, ≤ 0.01, and ≤ 0.001, respectively

*SlAlba2*, belonging to the RPP25-like family, is another flower-specific gene. This gene showed maximum expression in flower buds (> 96-fold higher than the control), followed by full blooming flowers (> 4-fold compared with the control). The expression of *SlAlba2* was 2.5-fold higher in breaker fruits than the control but significantly downregulated in 1 cm fruits, immature fruits, and B7 fruits during fruit development (− 1.7 to 4-fold vs. the control). Intriguingly, *SlAlba3* was the only gene whose transcripts were most abundant in leaves, followed by stems, and its expression was significantly reduced (by 4.5- to 14.8-fold relative to the control) throughout all stages of fruit development. *SlAlba6*, *SlAlba7*, and *SlAlba8*, homologous genes belonging to the same phylogenetic clade, exhibited a 2-fold upregulation in stem tissues. Compared to the control, their transcript levels were significantly reduced in fruits during most stages of development (Fig. 6).

### Expression analysis of tomato *Alba* family genes under different abiotic stresses and phytohormone treatments

**Fig. 7:**
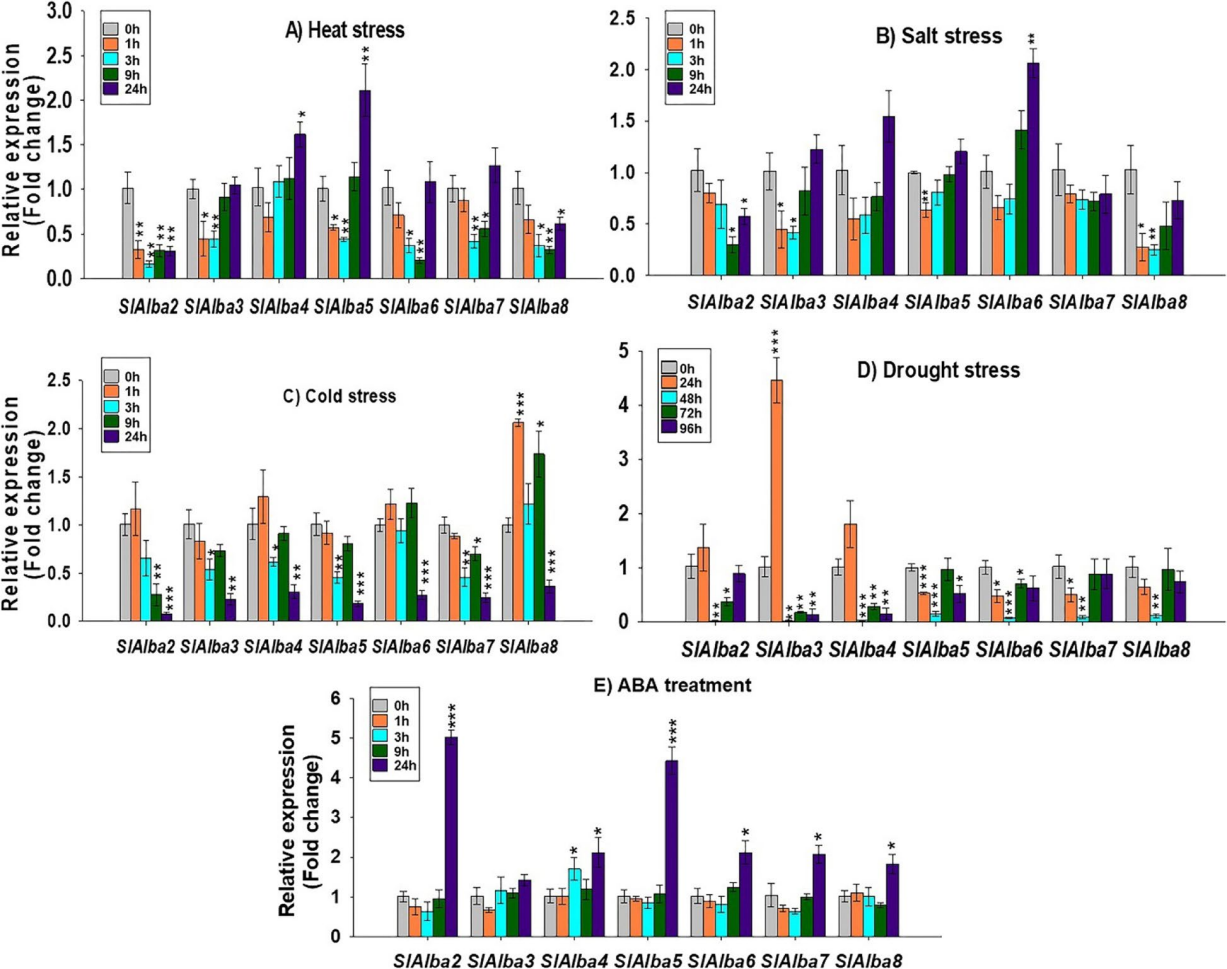
Expression analysis of *SlAlba* genes by RT-qPCR under different abiotic stresses: **a** heat, **b** salt (NaCl), **c** cold, **d** drought, and **e** ABA treatment. Error bars denote standard deviations of the means of three independent replicates. The asterisks indicate significant differences, as determined by Student’s t-test (* *p*-value ≤0.05, ** *p*-value ≤0.01, and *** *p*-value ≤0.001)

*SlAlba1* transcripts were undetectable in all leaf samples from control, abiotic stress-, or ABA-treated plants, whereas many of the other *Alba* genes displayed diverse expression patterns over time following exposure to these treatments (Fig. 7a-e).

Heat stress triggered significant changes in the transcript profiles of the *SlAlba* genes at various time points (Fig. 7a). Compared to the control (0 h), *SlAlba4* and *SlAlba5* expression significantly increased (1.6-to 2-fold) at 24 h after heat treatment. *SlAlba5* expression was repressed (1.7-to 2-fold) during the early phases of heat stress but gradually recovered after 9 h and was upregulated (2-fold) at 24 h after heat stress. By contrast, the majority of *Alba* genes (*SlAlba2*, *SlAlba3*, *SlAlba6*, *SlAlba7*, and *SlAlba8*) showed similar responses to heat treatment, with decreased expression levels (1.6-fold to 6-fold vs. the control) following heat exposure.

Most of the *Alba* genes were responsive to salt treatment but *SlAlba4* and *SlAlba7* showed no significant change in expression under salt treatment compared to the control (Fig. 7b). *SlAlba6* was induced by salt stress; its transcript levels did not significantly change during the early phases of salt treatment but gradually increased and peaked (2-fold vs. the control) at 24 h of exposure to salt stress. Several *Alba* genes, *SlAlba2*, *SlAlba3*, *SlAlba5*, and *SlAlba8*, displayed reduced transcript levels (1.5-to 4-fold lower than the control) at one or two time points during salt-stress treatment.

Seven *Alba* genes were differentially regulated in response to low temperature stress (Fig. 7c). *SlAlba8* was significantly upregulated (1.7- to 2-fold vs. the control) by cold treatment, while multiple *Alba* genes (*SlAlba2*, *SlAlba3*, *SlAlba4*, *SlAlba5*, *SlAlba6*, and *SlAlba7*) were downregulated (1.6- to 12-fold relative to the control) in response to cold stress.

The expression of *SlAlba3* was remarkably induced at 24 h after withholding watering for drought treatment, whereas most *Alba* genes were significantly deregulated (1.4- to 54-fold vs. the control) at one or multiple time points during the drought stress period (Fig. 7d). The *SlAlba* genes also showed similar expression patterns in response to the stress hormone ABA. Most of the genes were considerably upregulated (1.8- to 5-fold higher than the control) under ABA treatment; only one gene, *SlAlba3*, was unresponsive to ABA treatment (Fig. 7e).

### Subcellular location analysis of tomato Alba proteins

**Fig. 8:**
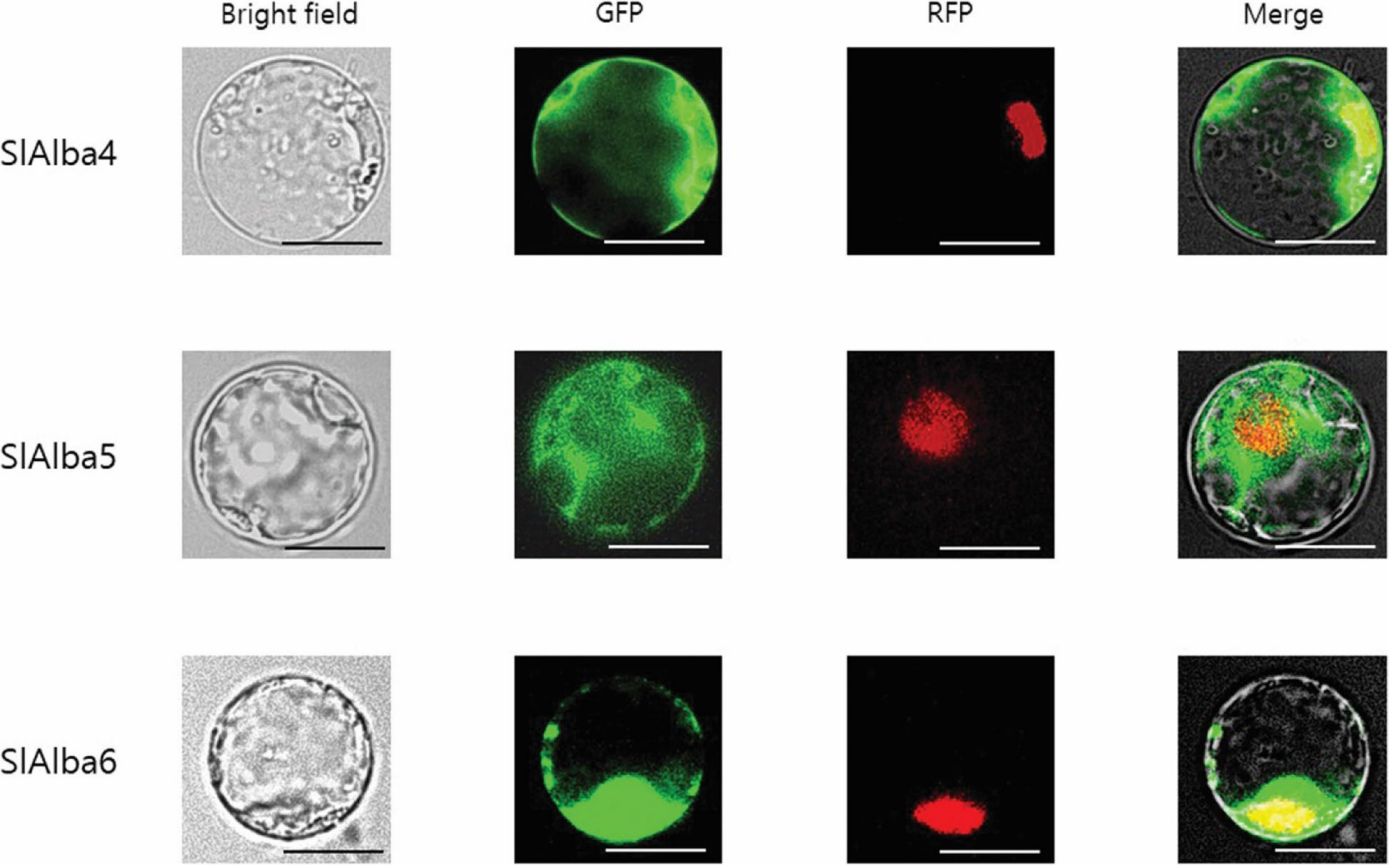
Protoplast transient expression analyses using Alba-GFP fusion constructs. Sub-cellular localization of SlAlba4, SlAlba5 and SlAlba6 were analyzed by transient expression using Oc cell protoplasts after 16 h incubation in dark at 28 C and observing with the confocal microscope. The NLS-mRFP construct was used as a nuclear localization marker. Scale bars = 10 μm

In silico subcellular location analysis suggested that the SlAlba proteins are localized to different parts of the cell, such as the cytoplasm, chloroplasts, and nucleus (Table S11). To verify their predicted localizations, three *Alba* genes (*SlAlba4*, *SlAlba5*, and *SlAlba6*) were used to generate fusion proteins with GFP. These proteins were expressed in rice protoplasts and their subcellular locations were examined. SlAlba4 was predominantly located in the cytosol, but could also be observed in the nucleus. SlAlba5 was primarily located in the cytosol, with strong signals detected in a large portion of the cytoplasm. SlAlba6 was detected in the nucleus as well as the cytoplasm (Fig. 8).

### Weighted gene co-expression network analysis and functional enrichment analysis

**Fig. 9:**
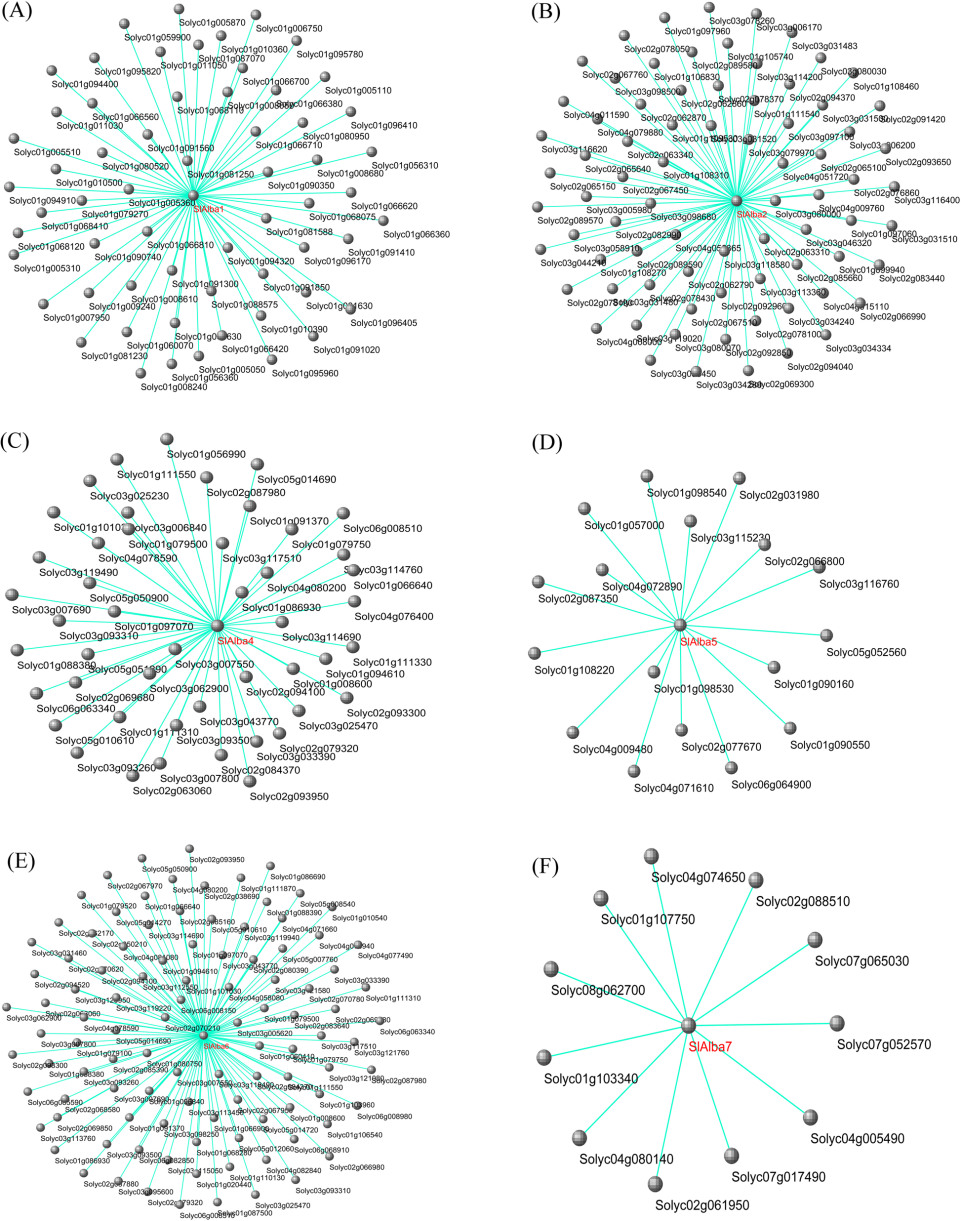
Co-expressed network analysis of 6 *SlAlba* genes which had a high correlation coefficient (0.63–0.89). **A**-**F** The co-expression network of *SlAlba1* (**A**), *SlAlba2* (**B**), *SlAlba4* (**C**), *SlAlba5* (**D**), *SlAlba6* (**E**), *SlAlba7* (**F**). The *SlAlba* genes are highlighted in red colour

The co-expression profiles of *SlAlba* genes in 18 RNA-seq data were analyzed using WGCNA to identify the relationship between the genes. A total of 315 genes were co-expressed with *SlAlba* genes, and the correlation coefficient ranged from 0.63-0.89 (Fig. 9). In details, we found that 60, 77, 47, 17, 103, and 11 genes were co-expressed with *SlAlba1*, *SlAlba2*, *SlAlba4*, *SlAlba5*, *SlAlba6*, and *SlAlba7*, respectively. However, we could not detect the co-expressed genes of *SlAlba3* and *SlAlba8* because they were low expressed in selected RNA-seq data.

**Fig. 10:**
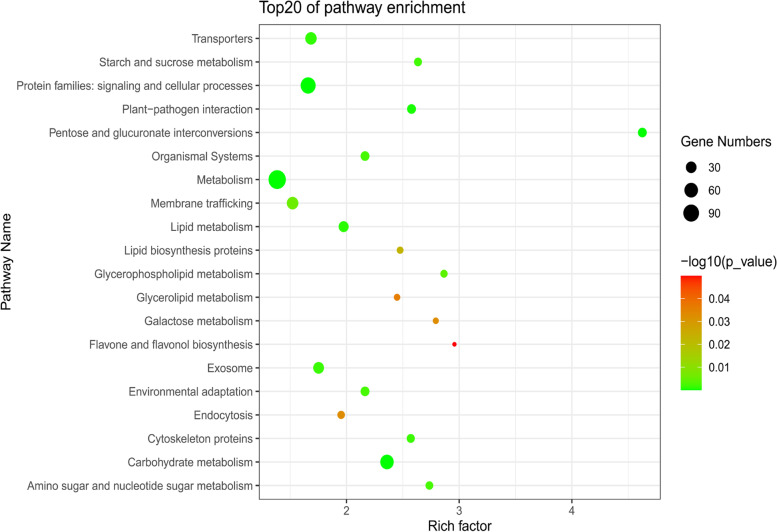
The enriched biological processes identified by KEGG analysis. The co-expressed genes of 6 *SlAlba* genes were selected for KEGG enrichment analysis (see Fig. 9). Enriched processes with FDR < 0.05 were included in the figure

The GO enrichment and KEGG enrichment analysis of the co-expressed genes showed that most of the genes identified in the co-expression network of *SlAlba* genes were involved in various biological processes, such as environmental adaptation, starch and sucrose metabolism, transport, signalling and cellular processes, plant-pathogen interaction, pentose and glucuronate interconversions, membrane trafficking, lipid metabolism, lipid biosynthesis proteins, glycerophospholipid metabolism, glycerolipid metabolism, galactose metabolism, flavone and flavonol biosynthesis, exosome, endocytosis, cytoskeleton proteins, carbohydrate metabolism, amino sugar and nucleotide sugar metabolism, pollen tube growth, unidimensional cell growth, cell wall organization or biogenesis, actin filament bundle organization, etc., while some of them have not been assigned to any biological pathway (Fig. 10, Fig. S8 and Table S13). Interestingly, a flower-specific gene *SlPLIM2a* (Solyc01g094320) in the gene co-expression network of *SlAlba1* [30], *SlCML8* (Solyc03g097100) and *SlCML27* (Solyc02g063340), which are related to environmental adaptation and highly expressed in floral buds, in that of *SlAlba2* [31], a heat-inducible gene *SlDEAH14* (Solyc05g014690) in that of *SlAlba4* [32], a heat-inducible gene *HSP101* (Solyc03g115230) in that of *SlAlba5* [33], a salt-inducible auxin-related gene *LAX2* (Solyc01g111310) and a heat-responsive gene *SlDEAH14* (Solyc05g014690) in that of *SlAlba6* [32, 34], and a salt-inducible gene *SlSYP51.2* (Solyc07g065030) in that of *SlAlba7* [35] were also identified, respectively.

## Discussion

The *Alba* gene family has been found in almost all kingdoms of life, including archaebacteria [1, 2], suggesting that these genes may have evolved before the divergence of archaea and eukaryotes. Genome-wide identification and comprehensive characterization of *Alba* gene family has been reported in some plant species, but the tomato *Alba* gene family has yet to be described. In this study, we identified eight *Alba* genes from tomato via genome-wide analysis. The number of *Alba* gene family members was observed to vary across diverse species of the plant kingdom (Fig. 1). *Alba* genes exist as a multi-gene family in tomato with the average number compared with other plant species, implying that they have biological importance in this plant species.

An in-depth analysis of the phylogenetic relationships among Alba proteins revealed two types. Some of the proteins are relatively large, with an RGG/RG repeat motif or other domain in addition to the generic single Alba domain; most of these proteins belong to the RPP25-like family. The other Alba proteins are relatively small, with only the generic single Alba domain; most of these proteins belong to the RPP20-like family in plants or the archaeal Alba family (Fig. 1). These findings suggest that the emergence of additional motifs and domains in Alba family proteins may have played a significant role in the expansion and diversification of the *Alba* gene family in plant species during the course of evolution.

Our phylogenetic analysis grouped the plant *Alba* genes into two distinct subclusters: *Alba* genes from monocots (rice, sorghum, and maize) and those from dicots (tomato, potato, Arabidopsis, chickpea, and grapevine). In addition, the only *Alba* gene (*CreAlba1*) identified in the genome of the single-celled green alga *Chlamydomonas reinhardtii* was clustered together with those of land plant species in the Rpp20-like phylogenetic group, indicating that common *Alba* genes are shared by chlorophytes and streptophytes, which diverged over 1 billion years ago [36]. Additionally, *Alba* genes from the moss *Physcomitrella patens*, the basal angiosperm *Amborella*, monocots (sorghum, rice, and maize), and dicots (tomato, potato, grapevine, Arabidopsis, and chickpea) were present within both eukaryotic-specific phylogenetic groups (Fig. 1), suggesting that these two families evolved before bryophytes and angiosperms diverged approximately 450 million years ago [37]. Overall, these findings enabled us to retrace ancient evolutionary transitions in this gene family.

From an evolutionary standpoint, gene duplication events increase the number of genes in a particular gene family, which can help plants adapt to adverse environmental stresses [38, 39]. One duplicated gene pair, *SlAlba6*/*SlAlba7*, was predicted in the tomato genome (Fig. S4), indicating that the eight *SlAlba* genes appear to have been derived from an original set of seven ancestral genes. Microsynteny analysis revealed three segmentally duplicated gene pairs between tomato and Arabidopsis, but only a single duplicated gene pair between tomato and rice as well as between Arabidopsis and rice. These results are consistent with the closer evolutionary relationship between tomato and the dicotyledonous model plant Arabidopsis than between tomato and the monocotyledonous model plant rice (Fig. 4).

Analysis of genetic structural diversity is indispensable for the evolutionary analysis of a multi-gene family. Detailed analyses of the conserved motifs and exon-intron structures of the *Alba* genes revealed that exons and introns as well as conserved motifs were organized in a similar pattern among phylogenetically closely related *Alba* genes but in a different manner among those from the different clusters. These findings point to the functional redundancy across phylogenetically closely related Alba family genes and present a likely rationale for the functional divergence among the divergent *Alba* genes during the evolutionary process (Figs. S1 and S2).

The predicted three-dimensional structure of a protein provides valuable information about its possible molecular functions and ligand-binding sites. To gain insight into further understanding of the molecular functions of SlAlba proteins, we predicted the 3D models of tomato Alba proteins. A previous study uncovered the likely binding affinity of rice Alba proteins to several molecules including DNA and RNA. In the current study, 3D-modeling of SlAlba proteins also predicted possible binding interactions with DNA, RNA, and peptide molecules (Fig. S6), supporting their putative functions in transcriptional and translational regulation (Table S12).

The proper transport of a protein to its specific subcellular locations is critical for its optimal activity. A previous subcellular localization experiment revealed that OsAlba1 localizes to the nucleus and cytoplasm [40]. In support of this finding, tomato *Alba* fusion proteins also showed GFP signals in the nucleus and cytoplasm (Fig. 8). These findings suggest that the diverse Alba proteins are involved in a variety of cellular signalling processes in the cytoplasm and nucleus.

Analyzing the expression patterns of a gene during growth and development and upon exposure to stress stimuli may help determine its functions. Thus, we analyzed the expression profiling of *SlAlba* genes in various organs and under different abiotic stresses. The majority of *SlAlba* genes except *SlAlba1* exhibited differential expression profiles across the organs examined, suggesting these genes play distinct regulatory roles in growth and development (Fig. 6). Several of the tomato *Alba* genes (*SlAlba3*, *SlAlba4*, *SlAlba6*, *SlAlba7*, and *SlAlba8*) were predominantly expressed in vegetative organs, but others, including *SlAlba1*, *SlAlba2*, and *SlAlba5*, showed higher transcript levels in reproductive organs such as flowers and fruits. These results suggest that these genes play preferential roles in these organs and developmental phases in tomato.

The initiation and development of a floral bud, a process modulated by multiple floral genes and environmental factors, plays a vital role in fruit set and crop yield [41]. Intriguingly, *SlAlba1* was expressed at strikingly higher levels in flower buds, fully bloomed flowers, and senescent flowers compared to root tissues and 1 cm fruits, and no expression was detected in other organs. *SlAlba2* was predominantly expressed in flower buds, with its expression level many times higher than that in any other organs (Fig. 6). These findings suggest that *SlAlba2* regulates floral bud formation in tomato and that *SlAlba1* regulates flower development at all stages via interactions with other regulatory genes.

The roles of *Alba* genes in fruit development have not previously been studied in any vegetable crop. Tomato is a model organism for the study of climacteric fruits. Therefore, the molecular pathways controlling tomato fruit enlargement (which includes a cell division stage and a cell elongation stage) as well as fruit ripening have been extensively explored [42–44]. Interestingly, *SlAlba5* is the only gene that showed higher expression in tomato fruits at all stages of development (except the immature stage), with its peak expression in 1 cm fruits. By contrast, the remaining seven *SlAlba* genes showed lower expression levels in fruits at all developmental stages compared to the control (leaves; Fig. 6). Our findings suggest that *SlAlba5* plays a regulatory role in fruit enlargement and ripening, particularly during the initial stages of fruit development.

Most tomato *Alba* genes that were highly expressed in vegetative organs had their highest expression levels in stems; only one gene had its highest expression level in leaves, and no gene has its highest expression level in roots. In addition to providing mechanical support to the aerial portions of the plant, the stem facilitates the long-distance translocation of water and nutrients to sustain plant growth under both normal and stressful conditions. The transcript levels of *SlAlba3*, *SlAlba4*, *SlAlba6*, *SlAlba7*, and *SlAlba8* were markedly higher in stems than in any other organ examined, suggesting that they likely function in stem growth, the long-range movement of water and nutrients, and stress tolerance. The higher expression level of *SlAlba3* in leaves suggests that it might play a role in leaf development and signalling cascades in leaves (Fig. 6). Taken together, these findings point to the diverse functions of *SlAlba* family genes in plant development.

Plant responses and adaptation to environmental stresses often involve differential gene expression, which is regulated by a dynamic network of numerous transcription factors and various stress tolerance genes in an ABA-dependent or -independent manner [45–48]. Alba proteins are associated with stress tolerance due to their involvement in genome packaging and organization, transcriptional and translational regulation, post-translational regulation, and RNA metabolism, in addition to responses to different environmental stresses [2, 22, 49]. The possible roles of *Alba* family genes in plant stress responses were supported by previous studies reporting the stress-induced expression of *Alba* genes in several plant species, such as cotton, Arabidopsis, and rice [15, 22, 23]. The role of OsAlba1 as a DNA binding protein involved in oxidative stress tolerance was revealed by complementation analysis in yeast. Moreover, the susceptibility of *ghAlba4-* and *ghAlba5*-silenced cotton plants to drought and salinity conditions highlighted the possible involvement of *Alba* genes in plant stress tolerance [23, 40].

We also observed that tomato *Alba* genes were differentially expressed in response to various abiotic stresses (Fig. 7a-e). *SlAlba4* and *SlAlba5* expression was significantly induced by heat treatment (Fig. 7a), which is in agreement with the finding that many *Alba* family genes in Arabidopsis and rice were markedly upregulated in response to mild heat stress (37 °C) and moderate heat stress (42 °C), respectively [15, 22]. Here we showed that *SlAlba6* was sharply upregulated in plants under saline conditions (Fig. 7b), suggesting its possible role in salt tolerance in tomato. This result is in agreement with the recent study finding that *Alba* genes were expressed at higher levels in cotton upon exposure to salt stress and that *ghAlba4* and *ghAlba5* cotton plants showed increased sensitivity to salt treatment compared to wild type and control plants [23]. The upregulation of *SlAlba3* at 24 h after withholding watering revealed its probable involvement in response to drought stress. This finding is supported by previous reports in which several *Alba* genes in rice and cotton were upregulated by dehydration treatment [22, 23]. The expression level of *SlAlba8* was considerably elevated following cold stress (Fig. 7c), which is consistent with the finding that a few rice *Alba* genes were upregulated in response to low temperature stress [22]. The phytohormone ABA is well known for its role in regulating plant acclimation to adverse environmental stresses, including heat, cold, salt, and drought [50–52]. All tomato *Alba* genes except *SlAlba3* were markedly upregulated under ABA treatment (Fig. 7e), which agrees with the finding that multiple *Alba* genes in rice were induced by ABA treatment [22]. Therefore, *SlAlba* genes might function in abiotic stress tolerance via their direct roles in ABA signalling.

In order to better understand the probable roles of *SlAlba* genes under various abiotic stresses, we analyzed the prevalence of the *cis*-acting elements in the upstream regions of tomato *Alba* genes. The regulation of tomato *Alba* genes under stress conditions was also corroborated by the prevalence of multiple *cis*-elements related to stress tolerance and hormonal responses in their promoter regions. Such elements might facilitate the regulation of these genes under different abiotic stress conditions (Fig. S5, Table S5).

Post-transcriptional regulation of numerous miRNA families plays a vital role in various biological processes, including development and stress tolerance [53, 54]. Several target sites for miRNAs related to stress tolerance and developmental processes were predicted in *SlAlba* genes (Table S6). This result is consistent with the finding that miRNA target sites are present in the *Alba* genes of various plant species including rice, Arabidopsis, maize, and sorghum [22].

To shed light on the putative functional roles of *SlAlba* genes in tomato, a gene co-expression network was constructed using the RNA-seq data. Multiple genes involved in several biological pathways showed co-expression with most *SlAlba* genes (Figs.9 and 10, Fig. S8, and Table S13), highlighting the biological importance of *SlAlba* gene family in tomato. In addition to co-expression with many genes related to several biological pathways, the floral-specific gene *SlAlba1* was co-expressed with a transcription factor *SlPLIM2a* (Solyc01g094320), which shows flower specific expression in tomato [30], suggesting that they might function together in the flower development of tomato [REF]. *SlAlba2*, which is highly expressed in floral buds, displayed co-expression with several genes related to plant-pathogen interaction and environmental adaptation (Table S13). In addition, it was co-expressed with *SlCML8* (Solyc03g097100) and *SlCML27* (Solyc02g063340), which are predominantly expressed in flower buds [31]. These findings corroborated the role of *SlAlba2* in abiotic stress response and flower bud development in tomato. *SlAlba4* and *SlAlba5*, which were upregulated by heat treatment, were also co-expressed with heat-inducible genes *SlDEAH14* (Solyc05g014690) and *HSP101* (Solyc03g115230), respectively, implying that they might have interaction for the adaptation of tomato to heat stress [32, 33]. Similarly, *SlAlba6*, which is upregulated by salt stress, showed co-expression with a salinity induced auxin-related gene *LAX2* (Solyc01g111310) [34], hinting that they might interact with each other for the improvement of salt tolerance in tomato. *SlAlba7* was co-expressed with a salt-inducible gene *SlSYP51.2* (Solyc07g065030) [35], a gene involved in environmental information processing (Solyc01g107750) and the genes related to chaperones and folding catalysts (Solyc02g061950 and Solyc08g062700) (Table S13), suggesting its important role in the abiotic stress response of tomato. Taken together, the findings of this study provide a valuable resource for further analysis to explore the biological functions of Alba proteins in tomato.

## Conclusions

In the current study, we identified eight *SlAlba* genes in tomato. These genes were clustered into two phylogenetic groups based on their domain and motif architectures. The likely involvement of these *SlAlba* genes in diverse signalling pathways, developmental cascades, and plant stress responses is underscored by the presence of development- and stress response-associated *cis*-regulatory elements and miRNA target sites in their sequences, their dual localization to the cytoplasm and nucleus, and their putative binding to several ligands including DNA, RNA, and peptides. Expression profiling revealed that most *SlAlba* genes are differentially expressed across various organs and in response to different stimuli. The expression pattern of *SlAlba5* suggests that it functions in fruit enlargement and ripening, especially during the cell division phase. Several *SlAlba* genes were significantly induced under different abiotic stresses, such as *SlAlba4* and *SlAlba5* by heat, *SlAlba6* by salt, *SlAlba8* by cold stress, and *SlAlba3* by drought stress, pointing to their possible roles in tolerance to these stresses. Co-expression network analysis highlighted the biological importance of *SlAlba* genes in tomato and the identified co-expressed genes with *SlAlba* genes supported their probable roles in the development and abiotic stress response of tomato. Our findings lay the foundation for further exploring the *SlAlba* gene family and provide new perspectives for the genetic improvement of tomato.

## Methods

### In silico identification and sequence analysis of tomato *Alba* genes

We identified tomato Alba gene family members from the Phytozome database (http://www.phytozome.net) using the keyword “Alba” [55] and performed BLAST searches of the Sol genomics database (http://www.solgenomics.net/tools/blast/) [56] using *Arabidopsis thaliana* Alba protein sequences obtained from TAIR (https://www.Arabidopsis.org/) as queries [57]. To validate the presence of the Alba domain, the resulting eight non-redundant Alba protein sequences were subjected to searches using the NCBI CDD search (https://www.ncbi.nlm.nih.gov/Structure/bwrpsb/bwrpsb.cgi) [58] and the SMART web tool (http://smart.emblheidelberg.de/) [59]. The exon/intron structures were visualized using the Gene Structure Display Server-2.0 web server (http://gsds.cbi.pku.edu.cn/index.php) by loading both *SlAlba* genomic and coding sequences [60]. The protein length (the number of amino acids), molecular weight, GRAVY values (grand average of hydropathicity index), and isoelectric points of the SlAlba proteins were determined using ProtParam (http://cn.expasy.org/tools/protparam.html) [61]. The Open Reading Frame Finder tool (https://www.ncbi.nlm.nih.gov/orffinder/) was used to analyze the open reading frames of the *SlAlba* genes. Clustal Omega (Clustal Omega < Multiple Sequence Alignment < EMBL-EBI) and ESPript web server (https://espript.ibcp.fr/ESPript/cgi-bin/ESPript.cgi) were used for multiple-protein sequence alignment [62, 63]. The web tool “Immunomedicine Group” (http://imed.med.ucm.es/Tools/sias.html) was employed to analyze the sequence homology of the eight Alba proteins. The MEME suite motif search tool (http://memesuite.org/) was used to examine the conserved motifs in protein sequences with the following parameters: maximum number of motifs 15 and motif length between six and 50 amino acids [64]. WoLF-PSORT (https://wolfpsort.hgc.jp/) was used to predict the subcellular localizations of the SlAlba proteins [65].

### Phylogenetic analysis

The full-length Alba protein sequences retrieved from the Phytozome and NCBI databases were aligned using Clustal Omega, followed by phylogenetic analysis via the neighbor-joining (NJ) method with 1000 bootstrap replications in MEGA 6.0 [66]. The names of the genes used to build the phylogenetic tree, together with their accession numbers, are described in Table S1.

### Prediction of miRNA target sites, *cis*-regulatory elements, and chromosomal locations

The putative miRNA targets were analyzed using the psRNATarget web tool (http://plantgrn.noble.org/psRNATarget/analysis). The PlantCare database (http://bioinformatics.psb.ugent.be/webtools/plantcare/html/) was employed to investigate the promoter region of each gene 1500 bp upstream of the initiation codon [ATG] to predict putative *cis*-acting elements in these promoters. The *SlAlba* genes were mapped to chromosomes using the online tool MapGene2Chrom web v2 (http://mg2c.iask.in/mg2c_v2.0/) after determining their chromosomal positions from the Sol genomic database.

### Gene duplication and microsynteny analysis

Gene duplications events across tomato Alba genes were identified with the one-step MCScanX program of TBtools software [67] and checked by BLASTP with the E-value < 10^− 10^. The identified duplicated gene pairs were visualized in Advanced Circos. The synonymous (Ks) and non-synonymous (Ka) nucleotide substitution rates of the *SlAlba* genes were computed following the method of Nei and Gojobori [68]. The Ka/Ks ratio was analyzed to determine the mode of selection [69]. The formula T = Ks/2r MYA (millions of years ago) was used to estimate the divergence time (T) for the duplicated gene pairs. Ks is the synonymous substitution rate per site, and r is the constant for dicot plants of 1.5 × 10^− 8^ substitutions per site per year [70]. Using a reciprocal BLAST search against the entire genomes of tomato, Arabidopsis, and rice, the microsyntenic relationship of *Alba* genes across these species was analyzed. The TBtools software was used to visualize the duplicated gene pairs [67].

### Comparative modelling of tomato Alba proteins

The 3D structures of the SlAlba proteins were predicted with the I-TASSER server using the protein sequences of SlAlba1-8 as input [71]. 3D models were generated by multiple threading of the alignments with LOMETS and performing iterative TASSER assembly simulations. The best-modelled structures with the maximum scores were selected and the template analogues were also identified. The resulting molecular models were refined with ModRefiner [72]. The functions of the modelled proteins were predicted using the I-TASSER server based on global and local similarity to template proteins in PDB with known structures and functions.

### Subcellular localization

The tomato *Alba* cDNA was amplified using gene-specific primers (Table S2) and cloned into the pGA3452 vector harbouring the maize *Ubi1* promoter, the synthetic GFP coding region, and the nos terminator [73]. NLS-mRFP under the control of the 35S promoter was employed as a nuclear marker. The constructs were cotransformed via electroporation into protoplasts prepared from rice Oc cells, and the resulting transformants were incubated overnight at 28 °C in the dark for 12 to 16 h. Fluorescent signals were visualized under a filter-equipped microscope (BX61; Olympus, Tokyo, Japan) using bright field illumination, the GFP channel, and the red fluorescent protein channel.

### Preparation of plant materials and stress treatments

Tomato plants (*Solanum lycopersicum* cv. Ailsa Craig) were grown in soil in a growth room under controlled conditions at a temperature of 25 °C during the day and 20 °C at night and a 16 h light 8 h dark photoperiod. For tissue-specific expression analysis, fresh roots, stems, and leaves were collected from 28-day-old plants. The remaining plants were transferred to a greenhouse maintained at 25/20 °C day/night temperatures until the reproductive stage to collect flower and fruit samples. Three different types of flower samples were harvested: floral buds, full-blooming flowers, and senescent flowers. Fruit samples were collected at six different stages: (i) young fruits approximately 1 cm in diameter at 2 weeks after pollination (1 cm fruits) (ii) immature fruits at 5 weeks after pollination (IM fruits), (iii) mature green fruits at 7 weeks after pollination (MG fruits), (iv) breaker fruits, when the colour of mature fruits changes from green to faint yellow-orange (B fruits), (v) breaker after 3 days (B3 fruits), and (vi) breaker after 7 days (B7 fruits) [74].

To study expression patterns of *SlAlba* genes under different abiotic stress conditions (abscisic acid [ABA], heat, cold, and salt [NaCl]), leaf samples from 28-day-old seedlings of uniform growth and development were collected at 0, 1, 3, 9, and 24 h after the start of the stress treatments [74], while they were collected at 0, 24, 48, 72, and 96 h after withholding watering for drought treatment. ABA treatment was applied by spraying leaves with 100 μM ABA. To impose heat and cold stress, the plants were incubated in a growth cabinet at 40 °C and 4 °C, respectively. Salt stress was imposed by submerging roots in a 200 mM NaCl solution. Drought treatment was applied to the plants by withholding watering after watering until the soil mixture is sufficiently wet. Plants grown in soil under normal conditions (25 °C) were used as the 0 h controls for all stress treatments. The samples were collected from three biological replicates, immediately frozen in liquid nitrogen, and stored at − 80 °C for RNA isolation.

### RNA extraction and qPCR expression analysis

Total RNA was isolated from the samples using an RNeasy Mini kit (Qiagen, Hilden, Germany) and purified with an RNase-free DNase I kit (Qiagen, Hilden, Germany) in accordance with the manufacturer’s protocols. RNA concentrations were determined with a NanoDrop® 1000 spectrophotometer (Wilmington, DE, USA), and 1 μg total RNA was used to synthesize cDNA using a Superscript® III First-Strand cDNA synthesis kit (Invitrogen, Carlsbad, CA, USA) as per the manufacturer’s instructions. Primer3 software (http://frodo.wi.mit.edu/primer3/input.htm) was used to design the gene-specific primers for all tomato *Alba* genes (Table S3). Melting curve analysis was performed to verify the specificity of the amplicon for each primer pair [22], and the expression of 18S rRNA (F: AAAAGGTCGACGCGGGCT, R: CGACAGAAGGGACGAGAC) was used as an internal control for normalization [75]. qRT-PCR was performed in a 10 μL reaction mixture consisting of 1 μL (50 ng) cDNA, 2 μL forward and reverse primers (5 pmol concentration), 5 μL of iTaq SYBR Green (Qiagen, Hilden, Germany), and 2 μL double distilled water. A Light cycler® 96SW 1.1 (Roche, Germany) was used to amplify and record the Cq value of each sample with the following conditions: pre-denaturation at 95 °C for 5 min followed by 40 cycles at 94 °C for 10 s, annealing at 58 °C for 10 s, and extension at 72 °C for 15 s. The 2^−∆∆Ct^ method was used to determine the relative transcript levels of each gene against each treatment [76].

### Statistical analysis

Data were analyzed with SigmaPlot 14 (SYSTAT and MYSTAT Products, United States, and Canada) using two-tailed Student’s t-tests. *P ≤ 0.05 and **P ≤ 0.01 and ***P ≤ 0.001 were considered statistically significant.

### Co-expression network analysis of *SlAlba* genes

Raw RNA-Seq data of 18 experiments with 3 repeats were downloaded from the NCBI SRA database under the accessions PRJNA639840, PRJNA484882, PRJNA756379, and PRJNA655574 (Table S14). Raw sequence reads were assessed with the FastQC toolkit [77]. The adapter contaminations and low-quality reads were removed using FASTP 0.19.4 with the parameters: -q 20 -u 40 –n 10 -l 50 [78]. The clean reads were mapped on tomato reference genome ITAG4.0 by using HISAT2 2.1.0 [79]. FeatureCounts 1.6.2 was used to count the number of reads that mapped on exons [80]. Further analyses were carried out based on fragments per kilo-base per million fragments mapped (FPKM) values. removeBatchEffect processing was performed to remove batch effect in R [81]. Weighted gene co-expression network analysis (WGCNA) was used to search for the co-expression genes with *SlAlba* genes. The genes with FPKM less than 1 were filtered out, and the remaining genes were input into the WGCNA v1.69 package in R for co-expression network construction [82]. Pearson correlation matrix and network topology analysis were used to calculate the gene correlation and soft thresholding power, respectively. The power, minModuleSize, and mergeCutHeight value were set to 16, 30, and 0.25, respectively. The networks were visualized using Cytoscape v3.8.2. The GO and KEGG annotations of co-expressed genes with *SlAlba* genes were conducted by pannzer2 [83] and KEGG Automatic Annotation Server [84].

## Supplementary Information

**Additional file 1: Figure S1.** Schematic representation of the exon-intron distribution of *SlAlba* gene family. **Figure S2.** Schematic representation of 15 conserved motifs in Alba proteins from tomato, Arabidopsis, and rice as predicted by Multiple Em for Motif Elicitation (MEME) web server. **Figure S3.** Chromosome distribution of tomato *Alba* genes. **Figure S4.** Gene duplication investigation of *Alba* genes in the tomato genome. **Figure S5.** Putative *cis*-acting elements in the upstream of *SlAlba* genes. **Figure S6.** The predicted binding of putative patterner ligands to SlAlba proteins. **Figure S7.** Overview of conserved motifs of Alba proteins from tomato, *Arabidopsis* and rice determined using MEME web tool. **Figure S8.** The top 30 enriched GO terms of co-expressed genes with 6 SlAlba genes. **Table S1.** List of the Alba amino acid sequences used for phylogenetic investigation. **Table S2.** The primer sequences used for subcellular localization analysis. **Table S3.** The primer sequences of *SlAlba* genes used for qRT-PCR analysis. **Table S4.** Sequence identity among 8 tomato Alba proteins. **Table S5.** List of *cis-*elements in the promoter regions tomato *Alba* genes. **Table S6.** Prediction of miRNA target sequences in tomato Alba genes. **Table S7.** Templates used for 3D structure modelling of SlAlba proteins. **Table S8.** Secondary structural components in SlAlba proteins. **Table S9.** Secondary structure prediction for SlAlba proteins by I-TASSER. **Table S10.** Parameters for 3D structure modelling of SlAlba proteins. **Table S11.** Subcellular localization of SlAlba proteins predicted by in silico analysis. **Table S12.** Gene Ontology (GO) annotation for SlAlba proteins. **Table S13.** Annotated pathways of co-expressed genes. **Table S14.** Information of samples used for RNA seq analysis.

## Data Availability

We declare that the dataset(s) required to reproduce the results of this article are included in the article and additional file(s) available in the journal webpage.

## Abbreviations

Alba: Acetylation lowers binding affinity
3D: Three Dimensional
GFP: Green fluorescent protein
IF3-C: Translation initiation factor 3 carboxy-terminal
Sir2: Sirtuin 2
Rpp20: Ribonuclease P protein subunit p20
Rpp25: Ribonuclease P protein subunit p25
NAD^+^: Nicotinamide adenine dinucleotide
RGG/RG: Glycine-arginine-rich
SnRNP: Small nuclear ribonucleoproteins
CLIP1: CAP-Gly domain-containing linker protein 1
ATP: Adenosine triphosphate
TM: Template modeling
RMSD: Root-mean-square deviation
C-score: Confident score
ABA: Abscisic acid
WGCNA: Weighted gene co-expression network analysis
