# Supplementary material for: Genome-wide identification and expression profiling of Alba gene family members in response to abiotic stress in tomato (Solanum lycopersicum L.)
Source: BMC Plant Biol. 2021 Nov 12;21:530. doi: 10.1186/s12870-021-03310-0 (PMC8588595; doi:10.1186/s12870-021-03310-0)
Supplement: Supplementary file 1 — Additional file 1: Figure S1. Schematic representation of the exon-intron distribution of SlAlba gene family. Figure S2. Schematic representation of 15 conserved motifs in Alba proteins from tomato, Arabidopsis, and rice as predicted by Multiple Em for Motif Elicitation (MEME) web server. Figure S3. Chromosome distribution of tomato Alba genes. Figure S4. Gene duplication investigation of Alba genes in the tomato genome. Figure S5. Putative cis-acting elements in the upstream of SlAlba genes. Figure S6. The predicted binding of putative patterner ligands to SlAlba proteins. Figure S7. Overview of conserved motifs of Alba proteins from tomato, Arabidopsis and rice determined using MEME web tool. Figure S8. The top 30 enriched GO terms of co-expressed genes with 6 SlAlba genes. Table S1. List of the Alba amino acid sequences used for phylogenetic investigation. Table S2. The primer sequences used for subcellular localization analysis. Table S3. The primer sequences of SlAlba genes used for qRT-PCR analysis. Table S4. Sequence identity among 8 tomato Alba proteins. Table S5. List of cis-elements in the promoter regions tomato Alba genes. Table S6. Prediction of miRNA target sequences in tomato Alba genes. Table S7. Templates used for 3D structure modelling of SlAlba proteins. Table S8. Secondary structural components in SlAlba proteins. Table S9. Secondary structure prediction for SlAlba proteins by I-TASSER. Table S10. Parameters for 3D structure modelling of SlAlba proteins. Table S11. Subcellular localization of SlAlba proteins predicted by in silico analysis. Table S12. Gene Ontology (GO) annotation for SlAlba proteins. Table S13. Annotated pathways of co-expressed genes. Table S14. Information of samples used for RNA seq analysis. [file 12870_2021_3310_MOESM1_ESM.zip › supplementary material.docx]

Supplementary Materials: Genome-wide identification and expression profiling of *Alba* gene family members in response to abiotic stress in tomato (*Solanum lycopersicum* L.)

Antt Htet Wai, Lae-Hyeon Cho, Muhammad Waseem, Xin Peng, Do-jin Lee, Je-Min Lee, Chang-Kil Kim and Mi‑Young Chung*


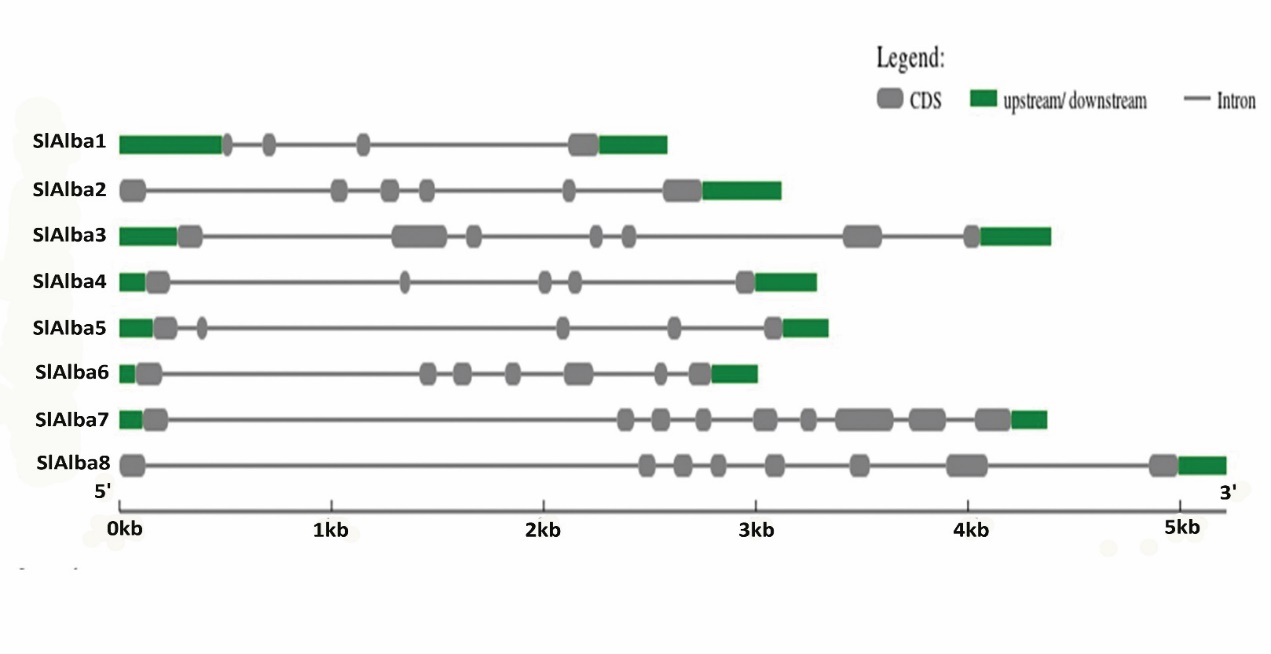


Figure S1. Schematic representation of the exon-intron distribution of *SlAlba* gene family. Gray boxes depict exons, black lines denote introns, and green boxes indicate untranslated regions. The lengths of exons/introns can be estimated using the scale below.


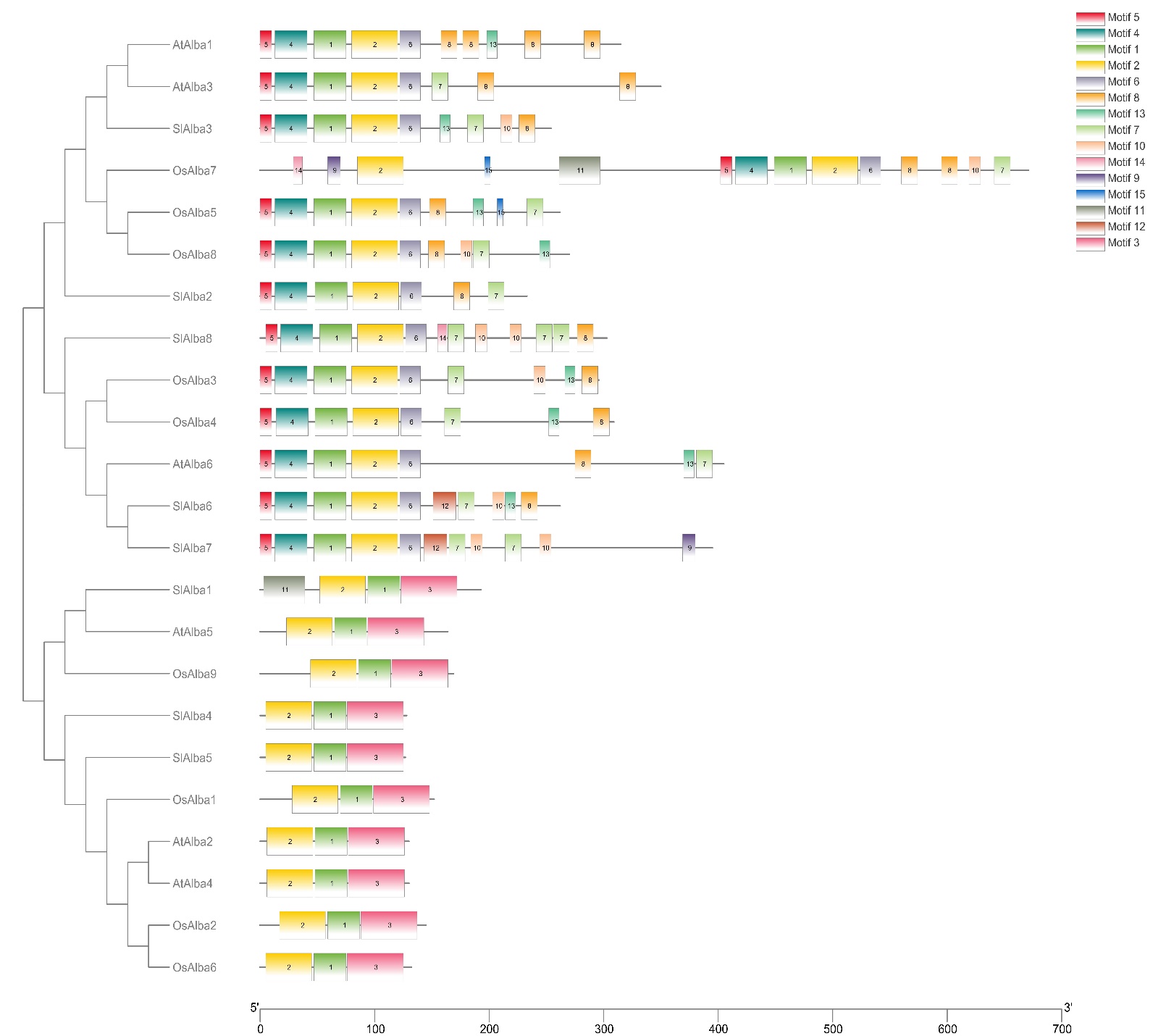


Figure S2. Schematic representation of 15 conserved motifs in Alba proteins from tomato, Arabidopsis, and rice as predicted by Multiple Em for Motif Elicitation (MEME) web server. The Alba domain contains two types of motifs viz, motif 1 and motif 2. Different motifs are denoted by different colored boxes with the motif names indicated in center.


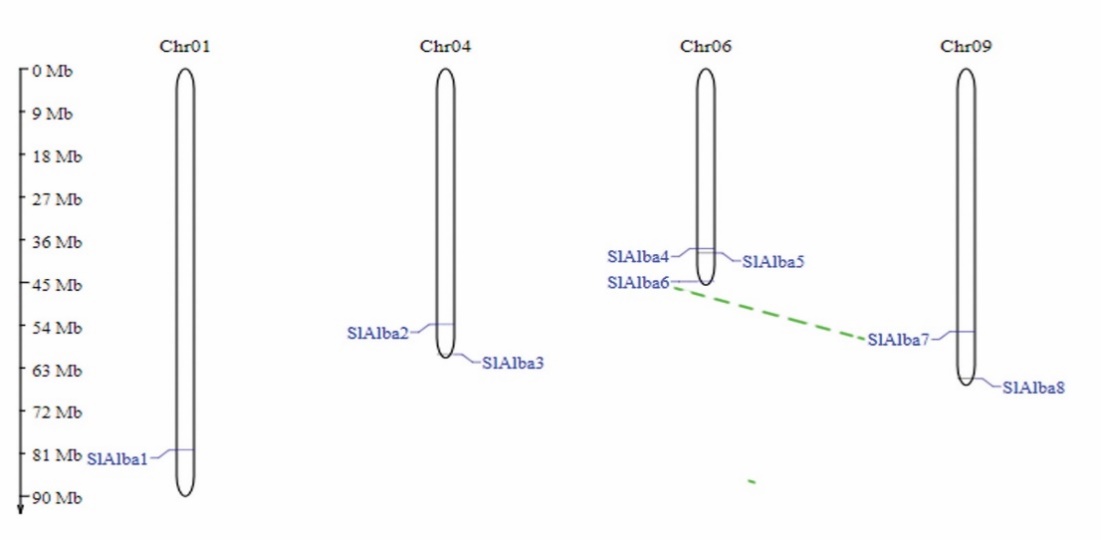


Figure S3. Chromosome distribution of tomato *Alba* genes with chromosome numbers indicated at the top of each chromosome and scale represents the length of chromosomes in megabases (Mb). The duplicated gene pair is connected by the green dotted lines.


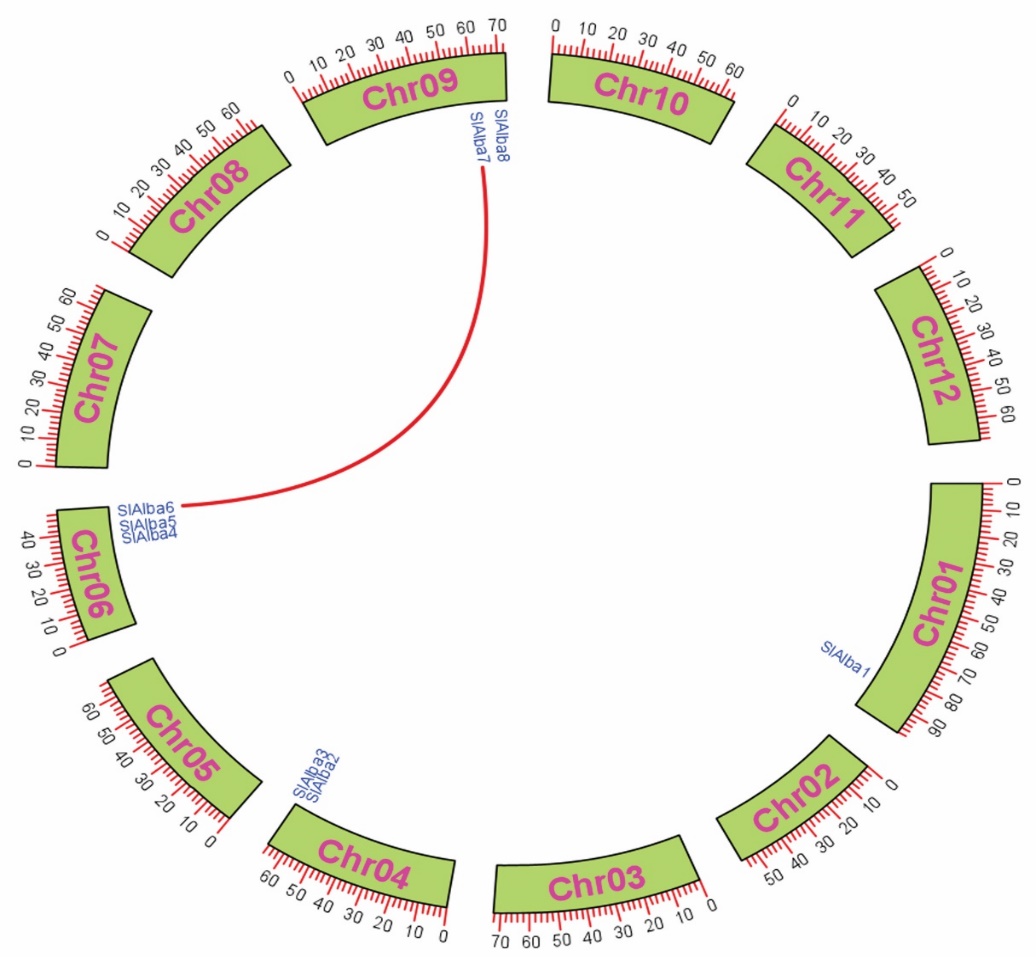


Figure S4. Gene duplication investigation of *Alba* genes in the tomato genome. Chromosome numbers and gene locations are depicted and the length of chromosome is scaled in Megabase pairs (Mbp). The segmentally duplicated gene pair, SlAlba6/SlAlba7, was connected by a red line.


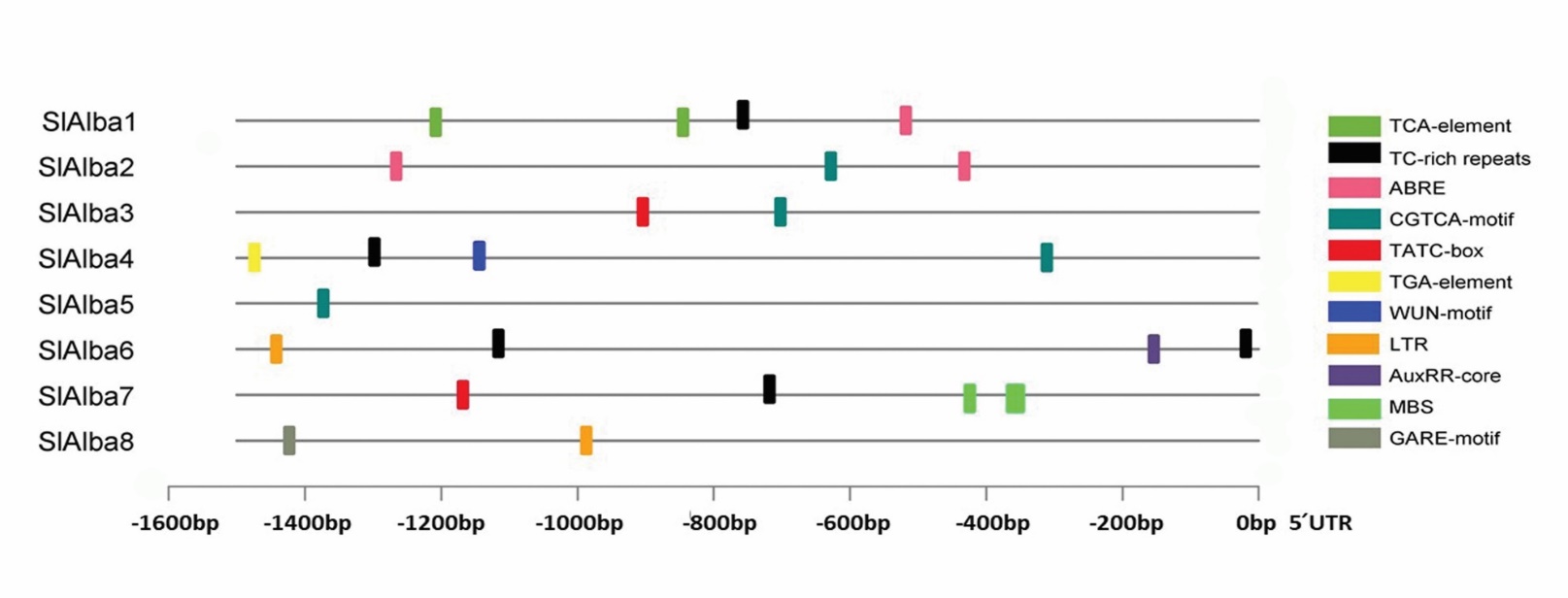


Figure S5. Putative cis-acting elements in the upstream of *SlAlba* genes. Predicted elements displayed are: a defense and stress-responsive element (TC-rich repeats), jasmonic acid-responsive elements (CGTCA-motif), auxin responsive elements (TGA-element and AuxRR-core), a ABA-responsive element (ABRE), a low-temperature-responsive element (LTR), gibberellin-responsive elements (GARE-motif and TATC-element), drought response related MYB-binding site (MBS), and a wound-responsive element (WUN-motif). The scale represents the location of each cis-regulatory element in relation to the translation initiation site (taken as +1 bp).


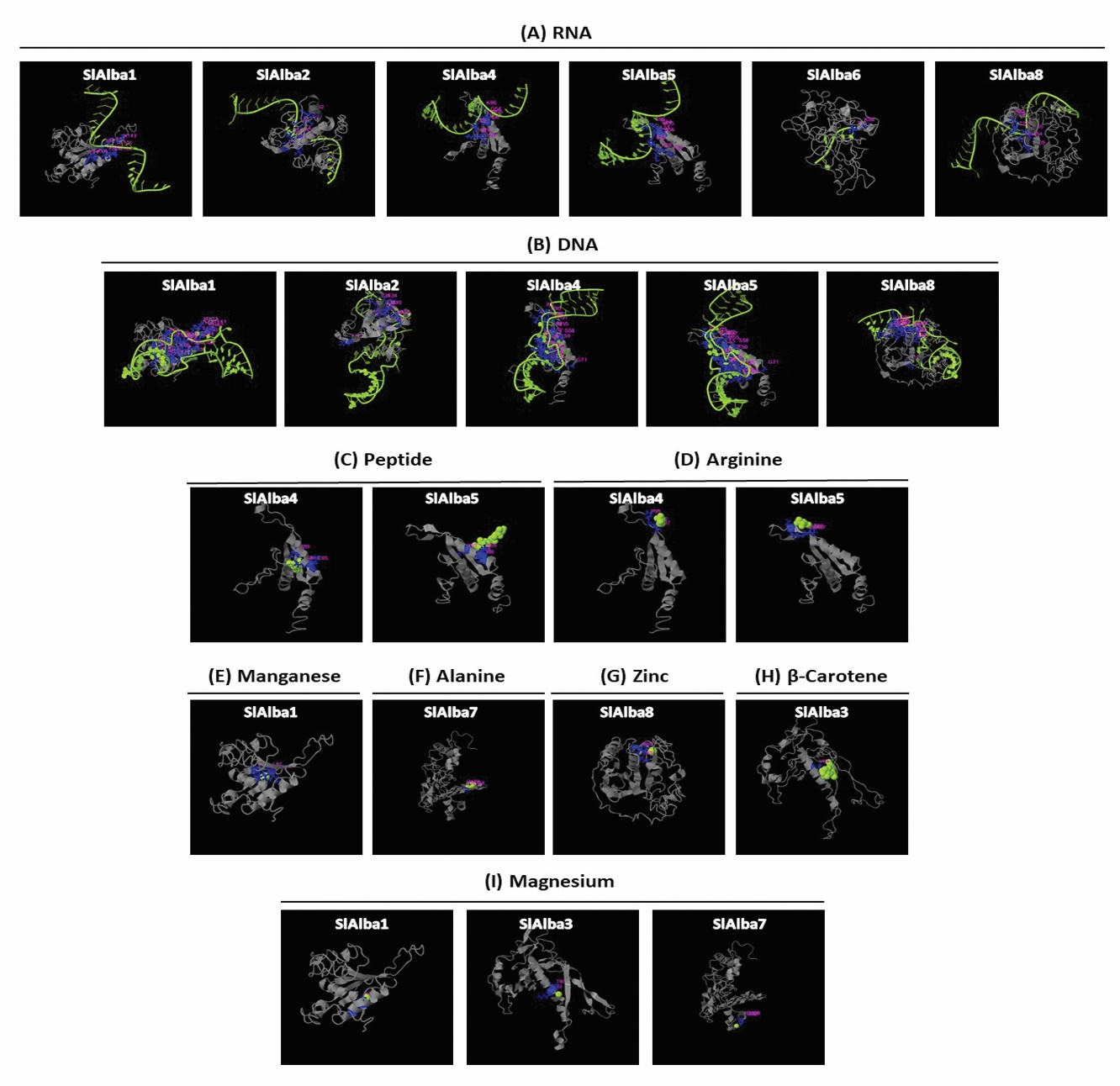


Figure S6. The predicted binding of putative patterner ligands to SlAlba proteins. SlAlba proteins binding to (A) RNA, (B) DNA, (C) Peptide, (D) Arginine (ARG) , (E) Manganese, (F) Alanine (ALA), (G) Zinc, (H) β-Carotene and (I) Magnesium.


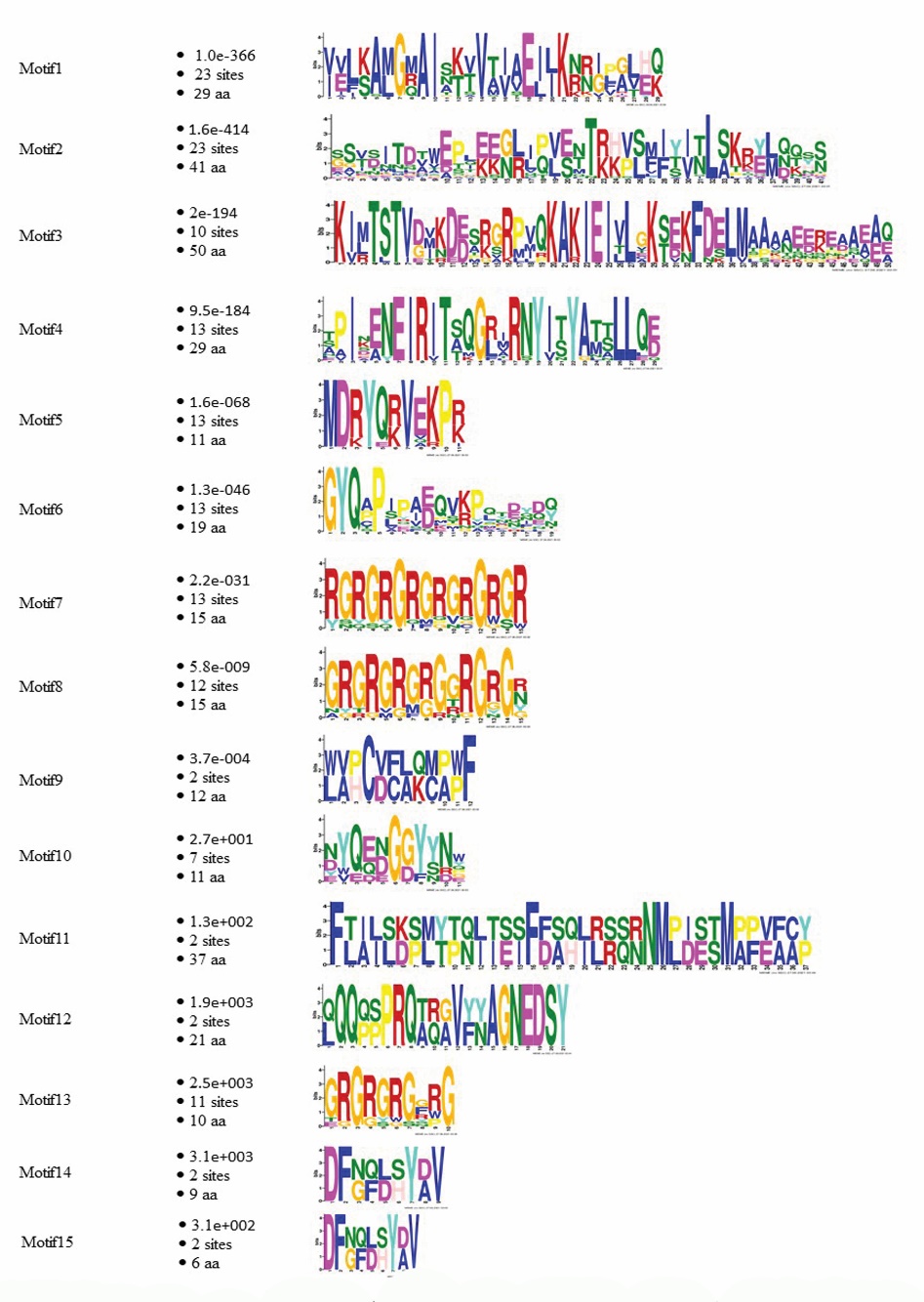


Figure S7. Overview of conserved motifs of Alba proteins from tomato, *Arabidopsis* and rice determined using MEME web tool.


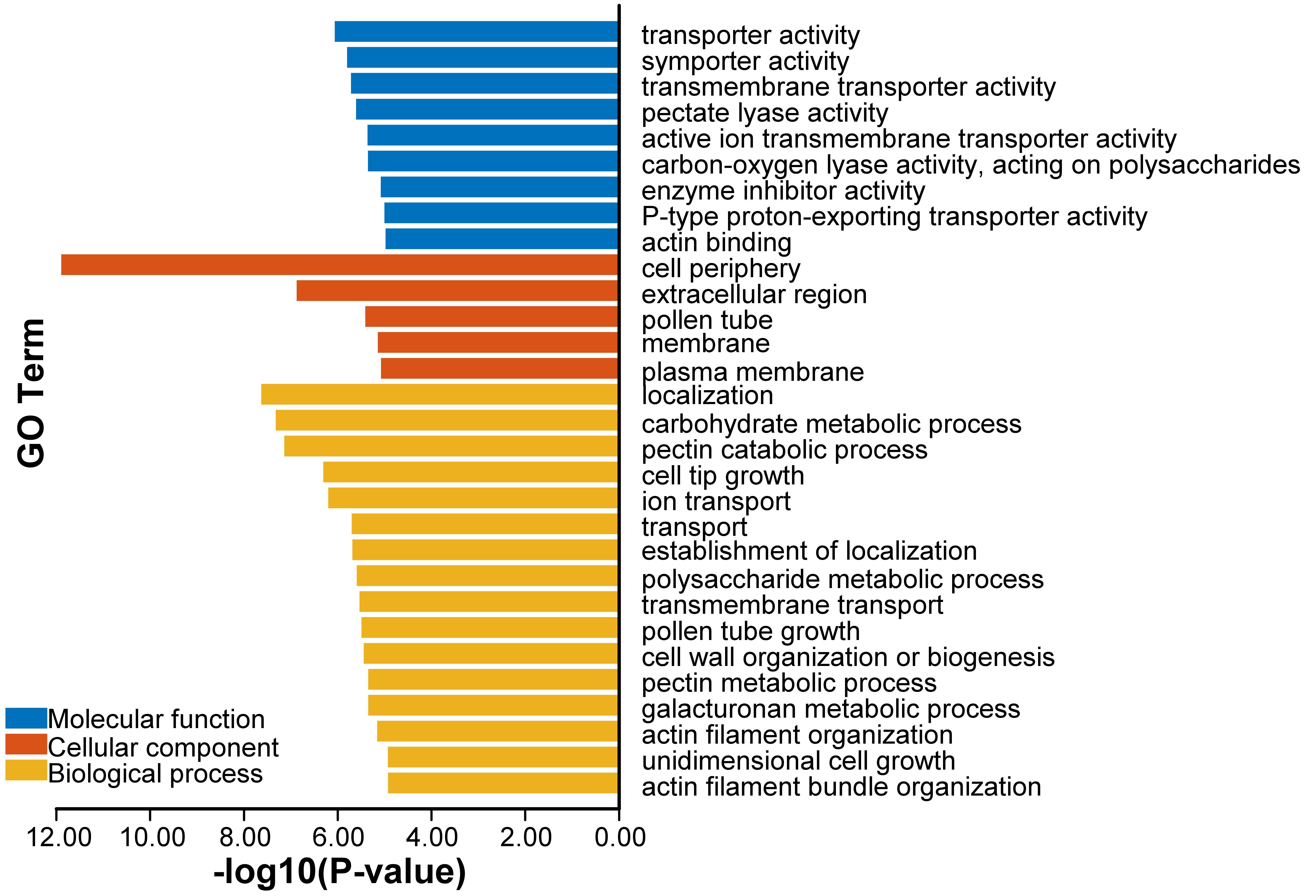


Figure S8. The top 30 enriched GO terms of co-expressed genes with 6 *SlAlba* genes.

| **Table S1.** List of the Alba amino acid sequences used for phylogenetic investigation | | |  |
| --- | --- | --- | --- |
| **Name** | **Accession** | **Sequence** | |
| SsoAlba1 | Ssol_1940 | MEKMSSGTPTPSNVVLIGKKPVMNYVLAALTLLNQGVSEIVIKARGRAISKAVDTVEIVRNRFLPDKIEIKEIRVGSQVVTSQDGRQSRVSTIEIAIRKK | |
| SsoAlba2 | Ssol_1942 | MTEKLNEIVVRKTKNVEDHVLDVIVLFNQGIDEVILKGTGREISKAVDVYNSLKDRLGDGVQLVNVQTGSEVRDRRRISYILLRLKRVY | |
| SshAlba1 | ssh10b | MSSGTPTPSNVVLIGKKPVMNYVLAALTLLNQGVSEIVIKARGRAISKAVDTVEIVRNRFLPDKIEIKEIRVGSQVVTSQDGRQSRVSTIEIAIRKK | |
| SshAlba2 | Alba2 | MTEKLNEIVVRKTKNVEDHVLDVIVLFNQGIDEVILKGIGREISKAVDVYNSLKDRLGDGIQLVNVQTGSEVRDRRRISYILLRLKRVY | |
| ApAlba1 | APE_1832a.1 | MSIEPQKPNTILVGRKPTINYVMAALKLLNEEGAPEVVIKARGRNICNAVDTVEMLKNLFIKNLVIKKVNIYSESLDSEGKKKVSAIEIVVAKG | |
| ApAlba2 | APE1823 | MACEGAPEVRIGRKPVMNYVLAILTTLMEQGTNQVVVKARGRNINRAVDAVEIVRKRFAKNIEIKDIKIDSQEIEVQTPEGQTRTRRVSSIEICLEKAGESA | |
| PhoAlba | PH1839.1 | MTEEHVVYIGKKPVMNYVLAVITQFHEGAKEVSIKARGRAISRAVDVAEIVRNRFLKDDVDVKEIKIGTEELPTADGRTTNTSTIEIVLARKT | |
| MthAlba | MTH_1483 | MSEENVVYIGNKPVMNYVLAVVTQMNGGTSEVILKARGIAISRAVDVAEIVRNRFIPDIQIENIDICTEEIIGNEGTATNVSAIEIQLRKD | |
| MjaAlba | MJ0212 | MDNVVLIGKKPVMNYVVAVLTQLTSNDEVIIKARGKAINKAVDVAEMIRNRFIKDIKIKKIEIGTDKVKNPDGREVNVSTIEIVLAK | |
| MmaAlba | MmarC5_1795 | MDNTVYVGNKGVMNYVLAVITQFNSENADEVIIKARGKAISRAVDVEEMVRKRFMSEIKIKEINLGTDHVQSEDGKSINVSTIEIILTK | |
| AfAlba1 | AF_1067 | MAENSVFVGNKPVMNYVLAVLTQFNSGATEVSIKARGRAISRAVDVAEIVRKRFLPDVDVKDIKISTEQIDSEQGTANVSAIEIILAKK | |
| AfAlba2 | AF_1956 | MAEHVVYVGNKPVMNYVLATLTQLNEGADEVVIKARGRAISRAVDVAEIVRNRFMPGVKVKEIKIDTEELESEQGRRSNVSTIEIVLAK | |
| PaAlba | PAE2226 | MATEQTILVGKKPTTNYVIATVMAFNAGVKKVVLKARGAAISKAVSTAVMVRDRFLPGKVQIKDIKLLSDKVQGQGGRERTVAAIEVVLEMA | |
| TaAlba | Ta0912 | MAEENIIFVGKKPTMNYVLAVVTQFNNNANKIIIKARGKTISKAVDVAEITRHKFIPDAKYEEIKLDTETLQGERGSSNVSSIEITLSR | |
| SlAlba1 | Solyc01g097050 | NILFLIILKSLTPNLIEIFFSHLLRQNNMPIETMAPVAAPTLATATPVSTPASAPTNNNNEAQKKSNRIQVSNTKKPLFFYVNLAKRYMQQHNEVELSALGMAITTVVTVAEILKNGGFATEKKVLTSTVGMKDEAKGRMVQKARIEIVLTKSEKFDKLMTPSNTNSDHAVAQDGAATNKNTITNDTKEQTKK | |
| SlAlba2 | Solyc04g071690 | MDRYEKVQKPIIDNPIKENEIRITTQGRLRNYITYATNLLLQEKGSKEIALKSMGRAISKTVMIAELIKRKIVGLHQITSIGSTDITDTWEPLEEGLLPLETTRHVSVITIILSKEELDTSSAGYQPPIPADQVRPLTEYDYGGGRFNDGVPGNGGEGGWKGGRGYNGRGRTGGRGRGNRGRGNNYGGVAGSMPIQGQGRGQGQGQGQVQGRGRGRAQGTKSNDTVRASVAYV | |
| SlAlba3 | Solyc04g081880 | MDRYQKVEKPRVETPIDANEIRITSQGRMRSYITYAMTLLQEKESEEIVFKAMGRAINKTVTIVELIKRRIVGLHQVTSITSTDITDTWEPLEEGLLPLETTRHVSMITITLAKKELDKNAVGYQPPLPADQVKVSTDFDYDGEGSPSGGRRGRGGRGRGRSRGFPGNGFMPAEYDDGGYDRNRSYGRGRGRGRGRSSRGRGRGGYNGPQDVQQDGGFYNQEAPMQGRGRGRGRGTRGRGRGFRSNGPIQGGGA | |
| SlAlba4 | Solyc06g065980 | MEGITDGVNKMSVSETQKKNRIQVSNTKKSLFFYVNLSKRYMQQYNEVELSALGMAISTVVTIAEILKNNGFAVEKKIRTLTVDMRDEPGARPIPKAKIEIVLGKTEKFDELMAAEAEQNGDNEEQQN | |
| SlAlba5 | Solyc06g068050 | MEELAEGVSNLNLVDSQKKNRIQVSNTKKPLFFYVNLSKRYMQQYNEVELSALGMAISTVVSIAEILKNNGLATEKKIMTSTVDVNEDMRGRPIQKAKIEIVLGKTVNFDELMLAGQKDGNNIQEQS | |
| SlAlba6 | Solyc06g083540 | MDRYQKVEKPKPELPINENEIRITSQGLVRNYISYATSLLQERSGKEIILKAMGQAISKTVAVAEIIKRRIPRLHQDAAISSVSITDVWEPIEEGLLPVEQTRHVSMISITLSTTELNKDSPGYQAPSDIQQTNYYNNNNNYAPRYSYQPRQQQPPPRQAQAVFNAGNEDSYDRGRGRGRGRGRGRGRGWNRGGYTNYQDGYDNYQENGGYSNWGRGGGRGGWGYRDSGYGRVRGGGGRGYGYGGGRGRMGNRPRGGNNNQA | |
| SlAlba7 | Solyc09g061710 | MDRYQKVEKPKPESPINEYEIRITSQGFVRNYINYANTLLQERHGKEIVLKAMGQAISKAVAIAEIIKRRVPILHQDTAISSVSITDVWEPIEEGLLPVEQTRHVSVISITLSTQELNNNSPGYQAPSEVEQMKPQYNNYQPQLQQQSPRQTRGVYYAGNEDSYGRGRGRGIGRGRGWSRGGYGNYQENGDYSNWGRENDGYSNRGRENGGISNRGRGNGGYSNRGRGNGGYSNRGRGNGGYSNWGGENGGYSNWGGGNGGYSNRGRGNGGYSNRGGENGAKEMVVTQIGAEKMVVTQTGAKEMVVTQTGAGKMVVTQTGAEEMVVTQTGAEVDGKVVTLDMKEAEVMDADVDAWTSVLKVVATRLSRGLAHCVFAQMPWFSSYYVQSRSKERKA | |
| SlAlba8 | Solyc09g091590 | SLYCGMDRYQKVEKPRPEEPIKENEIRVTAQGLIRNYISYATTLLQDQRTNEIVLKAMGQAISKTVAIAEIIKKRIPGLHQDTSISSTTITDAYEPLEEGLQPLEMTRQVSLITITLSTAELNQTSPGYQAPSRFDQSGAENQQLQQGKQAYVPSDFNQDSYDVRGRGRGRGRGRGRGRGRGRGGYGNYYQDDGGYYNPGRGGGLADDGTFYNPSRGGGFADNDGYYNQGRGGGFADNGGYYQQGQGGGRGRGWGYRGTGYGRGRGGGWGGGRGYSRGRGRMGGRGGRGGGNQYRQEEFAVKA | |
| StAlba1 | PGSC0003DMG400027494 | MTPVAAPTPATATPASTPAAPAPTNNNNNEAQKKSNRIQVSNTKKPLFFYVNLAKRYMQQHTEVELSALGMAITTVVTVAEILKNGGFATEKKVLTSTVGMKDEAKGRMVQKARIEIVLTKSEKFDKLMTPNNTNSDRAVAQDGAATNKTTITSDTKEQNKK | |
| StAlba2 | PGSC0003DMG400006363 | MDRYEKVQKPRIDNPIKENEIRITTQGRLRNYITYATNLLLQEKGSKEIALKSMGRAISKTVMIAELIKRKMVGLHQITSIGSTDITDTWEPLEEGLLPLETTRHVSVITIILSKEELDTSSAGYQPPIPADQVRPLTEHDYGGGRFNDGVPGNGGEGGWKGGRGYNGRGRTGGRGRGNRGRGNNYGAVAGSMPIQGQGRGQGQGQGQVQGRGRGRAQATKSNDPVRASVAYV | |
| StAlba3 | PGSC0003DMG400009952 | MDRYQKVEKPRVETPIDANEIRITSQGRMRSYITYAMTLLQEKESEEIVFKAMGRAINKTVTIVELIKRRIVGLHQVTSITSTDITDTWEPLEEGLLPLETTRHVSMITITLSKKELDKNAVGYQQPLPADQVKVSTDFDYDGEGSPSGGRRGRGGRGRGRSRGFPGNGFMPAEYDDGGYDRNRSYGRGRGRGRGRSFRGRGRGGYNGPQDAQPDGGFYNQEAPMQGRGRGRGRGTRGRGRGFRSNGPIQGGGA | |
| StAlba4 | PGSC0003DMG400028540 | MEELAEGVSNLNLVDSQRKNRIQVSNTKKPLFFYVNLSKRYMQQYNEVELSALGMAISTVVSIAEILKNNGLATEKKIMTSTVDVKEDMRGRPVQKAKIEIVLGKTVNFDELMLAGLKDGNNIQEQS | |
| StAlba5 | PGSC0003DMG400020460 | MEGITEGVNKMSVSETQKKNRIQVSNTKKSLFFYVNLSKRYMQQYNEVELSALGMAISTVVTIAEILKNNGFAVEKKIKTLTVDMRDEPGARPIPKAKIEIVLGKTEKFDELMAAEAEQNGDNEEQQN | |
| StAlba6 | PGSC0003DMG400020480 | MEGITEGVNKMSVSETQKKNRIQVSNTKKSLFFYVNLSKRYMQQYNEVELSALGMAISTVVTIAEILKNNGFAVEKKIRTLTVDMRDDPGARPIPKAKIEIVLGKTEKFDELMAAEAEQNGDNEEQQN | |
| StAlba7 | PGSC0003DMG401020090 | MDRYQKVEKPKPELPINENEIRITSQGLVRNYISYATSLLQERSGKEIILKAMGQAISKTVAVAEIIKRRIPRLHQDAAISSVSITDVWEPIEEGLLPVEQTRHVSMISITLSTTELNKDSPGYQAPSEIQQTNYYNNNNNNYAPRYSYQPRQQQPPPRQAQAVYNAGNEDSYGRGGGRGRGRGRGRGRGWNRGGYTNYQDGYDNYQENGGYSNWGRGGGRGGWGYRDSGYGRGRGGGGRGYGYGRGRGRMGIRPRGGNNNQA | |
| StAlba8 | PGSC0003DMG400003423 | MDRYQKVEKPKPESPINEYEIRITSQGLVRNYINYANTLLQERHGKEIVLKAMGQAISKAVAIAEIIKRRMPILHQDTAISSVSITDVWEPIEEGLLPVEQTRHVSVISITLSTQELNKNSAGYQAPSGVEQMKPRYNNYQPQLQQQSPRQTRAVYYAGNEDSYGQGRGRGIGRGRGWSRGGYGNYQENGGYSNWGRENGGYSNRGRENGGYSNRGRGNGGYSNRGRENGGYSNRGRENGGYSNRGRENGGYSNRGRWGEENGGYSNRGGENGGYSNRGRGNGGYSNRGRGNGGYSNRGRGNGGYSNRGRGNGGYSNRGRGNGGYSNRGRGNGGYSNRGRGNGGYSNRGRGNGGYSNRGRGNGGYSNWGRGGVRSGWEYRDINTGSGYGGGRGYGRGRTDLRPRGGGNQA | |
| StAlba9 | PGSC0003DMG400029627 | MDRYQKVEKPRPEEPIKENEIRVTAQGLIRNYISYATTLFQDQRTNEIVLKAMGQAISKTVAIAEIIKKRIPGLHQDTSISSTTITDAYEPLEEGLQPLEMTRQVSLITITLSTAELNQSSPGYQAPSRFDQSGAENQLLQQGKQAYVPSDFNQDSYGVRGRGRGRGRGRGRGRGRGRGGYGNYYQDDGGYNNPGRGGRLADDGAFYNPSRGGGFADNDGYYNQGRGGGFADNGGYYNQGQGGGRGRGWGYRGTGYGRGRGGGWGGGRGYSRGRGRMGGRGGRGTGYGRGRGGGWGGGRGYSRGRGRMGGRGGRGGGNQYRQEEFAVRA | |
| CreAlba1 | XP_001695217.1 | MSDAAAGRQPNRVQVSSNKKPLQFYLNLSKRLLNEYGEVELSALGLAVSNMVTVAEILKKDGWAVEKSIRTGLELLEHSAAVEEDGEGGAAPAAGGRSVSKPKMEVVLAKSADFDRLFAEHPPPPRGPKAAAADGEGEEVEAE | |
| PpAlba1 | XP_001768991.1 | MEEITEGVADMSVYDAHKKNRIQVSNTKKPLFFYVNLAKRYMQQHEEVELSALGMAIATVVTVAEILKNNGLAIEKRILTSTVDMKDETRGRPIQKAKVEIILGKSEHFDELMAAAAEERELAPEEDQVGP | |
| PpAlba2 | XP_001767525.1 | MDRYQRVEKPRPEIPINENEIRITTQGKMRNYITYATTLLQEKGASEIVLKAMGRAINKTVTIAEIIKRRIAGLHQNTSIGSTDITDLWEPLEEGLLPLETTRHVSMITITLSTKELETSSTGYQPPLPADQVKPLAEFEYDGDSPPRGDGGRGRGRGRGRGRGGCEMTTFEYDGGLDGGLVRGRGRGRGRGRGRGGYRGRGREQDAGGFDESNADGPPRDQGNDFSQETSYSGMCTFA | |
| PpAlba3 | XP_001753647.1 | MDRYQRVEKPRPEIPISENEIRITTQGKMRNYITYATTLLQDKGASAIVLKAMGRAINKAVTIAEIIKRRIAGLHQNTSIGSTDITDLWEPLEEGLLPLETTRHVSMITITLSTKELENSTGYQPPLLADQVKPLAEFEYEGDGSPRGGRGRGRGRGRGRGRGRGANETANSEYNGGLDGGWVRGRGRGRGRGRGEYRGRGREQDAGGFDEPNADGPLRDQGNGFSQDMSYSDRGRGRGRGRGQGRGQGRGFRTDGPSQDQSQAGAAQ | |
| PpAlba4 | XP_001755565.1 | MDRYQRVEKPRPEIPISENEIRITTQGKMRNYITYATTLLQDKGSSEIVLKAMGRAINKTVTIAEIIKRRIAGLHQNTSIGSTDITDLWEPIEEGLLPLETTRHVSMITITLSTKELEKSSIGYQPPLPADQVKPLVEFDYEGDGSPRGGRGRGRGRGRGRGRGNDVENSEYNGLDGGWGRGRGRGRGRGRGRGAYRGRGRDQDAGGFDEPNADGPPRGRGTDFPQETSYSGRGRGRGRGRGQGRGQGRGFRTDGPTQEQPQSGAA | |
| AmtrAlba1 | XP_020520141.1 | MAGGGAVPASAAKRESNCDNRRNQITVARNKPILFYVDVAKRRLKHNDEVVISGLGTAITSVVMISEILTNNGFAFQKKILTSRISMIDGNTGRVIQKARMEVVLGKTENFNQVLGQGSASPESPEAYENE | |
| AmtrAlba2 | XP_006847137.1 | MDRYQRVEKPRPDTPINENEIRITTQGRMRNYITYATSLLQEKGANEIVLKAMGRAINKTVMIAELIKRRIVGLHQNTAIGSTDITDMWEPLEEGLLPLETTRHVSMITITLSKKELDTSSTGYQSPLPADQVKPWNEFDYEGEGSPGLRGRGRGGRGRGRPRGNMNGVGEYNGDGGWEGGRGYGRGRGRGRGRPFRGHGRGYGGGEMQQEAGGYNDFGGPDAPPAQGRGRGRGRGRGRGRGGRGDYRSDGPIQAAAAGAEAG | |
| AmtrAlba3 | ERN18878.1 | MDRYQRVEKPRPESTINENEIRITTQGLIRNYISYATSLLQEKRVREIVLKAMGQAISKTVAIAEIIKKRMPGLHQETSISSTSITDVWEPIEEGLVPLEMTRHVSMISISLSTKELNKNSPGYQAPASLEQHPPQQQQKSHMAKEIYMDGGEDEVVAGEEAGVGVEVITIKGMTTKGTTIKVITKDTVTIKYITIRVITTKDIKKMEGGTTIIIGTEEVVEEEGGAIVVAEEDMREVGEEEGAMVEGVEGRAGGAEEIENGGRLYFHYGWEIWPLYYFPPFIHLSMYSYPFSIWSDLDF  GFD | |
| AmtrAlba4 | XP_006837817.1 | MAAIEEVTEAVSNLHVSESHKKNRIQVSNTKKPLFFYVNLAKRYMQQHNEVELSALGMAIATVVTIAEILKNNGLAIEKKIMTSTVDMKDESRGRPIQKAKIEILLGKTANFDELMAAAAEEREIAEGEEQS | |
| VvAlba1 | XP_002280347.1 | MEAIVVAENLEESMKKSSLGNATETQKKNRIQVSNSKKPLFFYINLAKRYIKQYNDVELSALGMAIPSVVTIAEILKKNGVATQKKILTSTVDMKWETNGRTVQKAKIEIVLGKPEDSDNQAAATAVAAAAAATPAEENADKKE | |
| VvAlba2 | XP_010652481.1 | MDRYQRVEKPRPESTINENEIRITAQGVIRNYISYASTLLQEKRVREIVLKAMGQAISKTVAIAEIIKKRIPRLHQDTAISSVSITDVWEPIEEGLVPLEMTRHVSMISITLSTRELNKNLPGYQAPYHVEQPKSQYHYQPQNQPRQAARPPYNAVNEDSYGRGRGRGRARGRGWGRGGYGNYQDNGGYNGGYPNWGRGGGRGRGWGPYRGAGYERGRGGGGRGYGRGRGRMGGGRGRGGGNQA | |
| VvAlba3 | CBI21553.3 | MQVDLIVKSLVLPPSRTPERARTQLPTMDRYQRVEKPRTETPINENEIRITTQGRMRNYITYATTLLQEKGSNEIVLKAMGRAINKTVMISELIKRRIVGLHQNTSIGSTDITDMWEPLEDGLLPLETTRHVSMITITLSKKELDTSSTGYQPPIPVDQVKPLNELEYEGDSPGMRGRGRGRGRGRGRGNYNGVGGEYNNGDGWDGGRGFGGRGRGRGRGRGFRGRGRGYGGGDMQYESGGYYDYGDSEVPAQGRGRGRSRGRGRGRGRNFRTEGPPQAIAA | |
| VvAlba4 | CBI36290.3 | MEEITEAVNNINISDLHKKNRIQVSNTKKPLFFYVNLAKRYMQQHNEVELSALGMAIATVVTIAEILKNNGLAVEKKITTSTVDMKDESRGRPVQKAKIEILLGKTANFDELMAAAAEEREAGDVEEQS | |
| VvAlba5 | XP_002285115.1 | MEEITEGVNSMNIADSHKKNRIQVSNTKKPLFFYVNLAKRYMQQHNEVELSALGMAIATVVTIAEILKNNGLAVEKKIMTSTVDMKDESRGRPIQKAKIEILLGKTENFDELMAAAAEEREAGDVEEQQS | |
| VvAlba6 | CBI18930.3 | MGRRGHTALLSLLLSLVLFYVFEHESIVRAVSIPAKFDGFVYNYTGTGIDSIIIEAFFDPVCPDSRDAWPPLKRAIAYYAPRVSLIVHPFALPYHDNAFATSRALHIVNKLNSSATYHLLEMLFKHQEIFYNQITVNMSRTAIVDCIVKFVSKAVGESLFSAIKSGFSDRQTDLTTRVSFKYGCSRGVLGTPYFFVNGFPLPDPGSAINYSKWRSILDPLPFLNIGPKLRQKRSQRRGLFLRGASILSLQKNIQSEKMDRYQRVEKPREETPIDENEIRITSQGRMRSYITYAMSLLQEKGSNEIVFKAMGRAINKTVTIVELIKRRIVGLHQNTSIGSTDITDTWEPLEEGLLPLETTRHVSMITITLSKKELNTSSIGYQPPLPAEQVKAWTEFDYEGEGSPSGRGRGRGGRGRGRTRGVSGNGFVSAEYEDGGWDRNRGYARGRGRGRGRGFRGRGRGGYNGPQVDAQQDMGGYNQEPPFQGRGRGRGRGNRGRGRGARSNGPIHATAGGA | |
| VvAlba7 | XP_002264932.1 | MDRYQRVEKPRPETPINENEIRITAQGRMRNYITYATTLLQDKGSDEIALKAMGRAINKTVMIAELIKRRIAGLHQNTSIGSTDITDMWEPLEEGLLPLETTRHVSVIAITLSKKELDISSTGYQPPIPADQVRPWTEYDYEGEGSPAMRGRGRSGRGRGRGRGNNNNGMVEYNGDGGWDGVRGGYGGRARGRGRGRGYRGRGRGYGGGDMQQEMGGYNDYGSGAVPAQGRGRGRWRGRGRGRGPGRDIKSDGPVQAAA | |
| CaAlba1 | XP_004486682.1 | MEGITEGLNNININNNSDSYKKNRIQVSNTKKPLFFYVNLAKRYMQQHNEVELSALGMAIATVVTVAEILKNNGLAVEKKIMTSTVDIKDDSRGRPVQKAKIEIVLGKTANFDELMAAAAAEDAENGDIEEQTA | |
| CaAlba2 | XP_004495605.1 | MTTIAAAVTATPTTNGNGNDSHKKNRIQVSNTKKPLFFYVNLAKRYIQQHNEVELSALGMAIATVVTIAEILKNNGFATEKKVLTSTVGMKDENKGRLVQKAKIEIVLGKSEKFDNLMAPPATKTESEATTDDNK | |
| CaAlba3 | XP_004502885.1 | MDRYQRVEKPKADTPINENEIRITTQGRMRNYITYATTLFQEKGSDEIVLKAMGRAINKTVMITELIKRRIVSLHQITQIGSTDITDTWEPLEEGLLPLETTRHVSMITITLSKKELDTSSTGYQPPLPADQVKPLNEYEEEAEGSPRMRGRGRGRGRGRGRGRGMYNGGMEYGDGWDGGRGFGGRGRGRSWGRAFQGRGRGGYGAQPVGYYDNGEYDAPPAPRGRGRGRGRGRGRGRDAGRGAPA | |
| CaAlba4 | XP_004502886.1 | MRPRPHNPTHQRFYPACVAYLLSFLGLKLKPQIKSISPKPVNCVALTYSPKPTHPASLSFHSRTTQTMDRYQRVEKPKADTPISENEIRITTQGRMRNYITYATTLFQEKGSDEIILKAMGRAINKTVTITELIKRRIVGLHQNTLIGSTDITDTWEPLEEGLLPLETTRHVSMITITLSKKELDTSSIGYQPPLPADQVKPLNEYEEEGEGSPRIRGRGRGRGRGRGRGRGIYNGGMEYGDGWDGGRGYGGRGRGRAWGRSFQGRGRGYGAQPVGYYDNGEYDAPPAPRGRGRGRGRGRGRGRDAGRGAAA | |
| CaAlba5 | XP_004502888.1 | MDRYQRVEKPKPETPISENEIRITTQGRMRNYITYATTLFQEKGSDDIILKAMGRAINKTVMITELIKRRIVGLHQNTQIGSTDITDTWEPLEEGLLPLETTRHVSMVTITLSKKELDTSSTGYQPPLPVDQVKPLNEYEDEGEGSPRMQGRGRGRGRGRGRGRGMYNGGMEYGDGWDGGRGYGGRGRGRAFRGRGQSYGAQPVTYYDYGEYDAPPALRGHGRGRGRGRGRGRNAGREAAA | |
| CaAlba6 | XP_004506910.1 | MEAITEGVNNINISDSIKKNRIQVSNTKKPLFFYVNLAKRYMQQHNEVELSALGMAIATVVTVAEILKNNGLAVEKKIMTSTVDIKDDSRGRPVQKAKIEILLGKTANFDELMAAAAAAAEDGENGDVEEHAA | |
| CaAlba7 | XP_004511396.1 | MEAIVTNEMKNAKINGEVEKVTKIFRIQVSKTKKPLFFYLNLAKKHIKMGNDVELCALGLAIPTIIVIAEILKNNGWAIEKSIMTSTIEAKEDKEGRGAPKAKLDILLGSAKSVDQSTGSARE | |
| AtAlba1 | NP_564108.1 | MDKYQRVEKPKADTPIAENEIRITSMGRARNYITYAMALLQENKSNEVIFKAMGRAINKSVTIVELIKRRIPGLHQITSIGSTDITDTWEPTEEGLQTIETTRHVSMITITLSKEQLNTSSVGYQCPIPIEMVKPLAEIDYEGQDGSPRGRGGRRGRGGRGRGRGRGGRGNGPANVEYDDGGRGRGGRGNGYVNNEYDDGGRGRGGRGSGYVNNEYNDGGMEQDRSYGRGRGRGRGGGRGGRGRGGYNGPPPPYYEAQQDGGDYGYNNVAPPADHGYDGPPPQGRGRGRGRGGRGRGGGRGGFNRSNGAPIQAAA | |
| AtAlba2 | NP_564325.1 | MEEITEGVNNMNLAVDTQKKNRIQVSNTKKPLFFYVNLAKRYMQQYTDVELSALGMAIATVVTVAEILKNNGFAVEKKIMTSTVDIKDDSRGRPVQKAKIEITLAKSEKFDELMAAANEEKEAAEAQEQN | |
| AtAlba3 | NP_565124.1 | MDKYQRVVKPKADTPIDANEIRITSQGRARNYITYAMTLLQDKGSTEVVFKAMGRAINKTVTIVELIKRRIPDLHQNTSIGSTDITDTWEPTEEGLLPLETTRHVSMITITLSKIELNTSSVGYQCPIPIELVKPMGDIDYEGREGSPGGRGRGRGRGRGRGRGRGGRGNAYVNVEHEDGGWEREQSYGRGRGRGRGRSSRGRGRGGYNGPPNEYDAPQDGGYGYDAPHEHRGYDDRGGYDAPPQGRGGYDGPQGRGGYDGPQGRRGYDGPPQGRGGYDGPSQGRGGYDGPSQGRGGYDGPSQGRGGYDGPQGRGRGRGRGRGGRGRGGGRGGDGGFNNRSDGPPVQAAA | |
| AtAlba4 | NP_565781.1 | MEEITDGVNNMNLATDSQKKNRIQVSNTKKPLFFYVNLAKRYMQQYNDVELSALGMAIATVVTVTEILKNNGFAVEKKIMTSTVDIKDDARGRPVQKAKIEITLVKSEKFDELMAAANEEKEDAEAQVQN | |
| AtAlba5 | NP_187113.1 | MAMEVATPAPAPIPSERNIVLAPATTTTTATVETHKKNRIQVSNTKKPLFFYVNLAKRYIQQHNEVELSALGMAITTVVTISEILKNNGLATEKKVLTSTVGMKDETKGKMVQKAKIEIVLGKSDKFDSLVPPVTNGKTPEEEASAETEASVEAQEEVAAATEV | |
| AtAlba6 | NP_187359.2 | MDRYQRVAKPKPESPINENEIRITSKGLIRNYISYATSLLQEKSVKDIVLKAMGQAISKTVAISEILKNKIPGLHQDIAISSISITDVWEPTEEGLFPVELTRHVSMISITLSLSELNKDSPGYQAPAQSDQSKPQYQPQQGRQARLPYNAYGEEGEVVAEGEAGEEVDMETTKGVMKEKTKGTIKKIIKTMKVGIQTRAEAVDVVDEAMAIVGGRGGYGGGRDGGYGGGRDDGYGERRNDGYGERRNDRYGGGRDDGYGGGRDDGYGGGRNDGYGGRRGGFRGGRGGGRDEGYGGGRGGYGGRSGGQGDGYGGGRGDGYGGGRGDGYGGGRGDGYGGGRVDRYDGGRRDGYGGGRYDGYGGGKSDGYGGGRGGYRGGRGGYGRGRGRMGNGGRSRDGASNQNEA | |
| OsAlba1 | NP_001042157.1 | MAVEEITEGVRNLAVEGEPAAAAAAAGGGGEGAQRRAAGSSSNRIQVSNTKKPLFFYVNLAKRYMQQHGDVELSALGMAIATVVTVAEILKNNGFAVEKKIRTSTVEINDESRVRPLQKAKIEIVLEKSEKFDELMAAAAEEREAAEAEEQA | |
| OsAlba2 | BAD15498.1 | MEEVTEAVGNLTIAAGEAAGAGGGAAEAHKKNRIQVSNTKKPLFFYVNLAKRYMQLHNEVELSALGMAIATVVTVAEILKNNGLAVEKKIMTSTVDVKDDSRSRPMQKAKIEIVLGKTDKFDELMAAAAEEREAAAAEAEAEEQS | |
| OsAlba3 | EEE59881.1 | MDRYQRVERPRPESAIEENEIRITAQGLIRNYVSYATSLLQDRRIKEIVLKAMGQAISKSVAVAEIIKKRVPGLYQDTNISSVSITDVWEPIEEGLVPLEMTRHVSMISITLSPRDLDKNSPGYQTPVYVEQPRQQPRLQQAPPPPQRQVRQPPPDYEDSYVRGRGRGRGRGRGRGWGRGGYGGYGGYGNNQGGYNQGGGYYDNQGGYGGYDNQGGYGGYDNQGGYGGGGYGYNQGRYGNYQENGGYNRGRGGMRGRGNWNYRGGYERGRGGGFPGGRGYGGRGRGRMGGRGGRGN | |
| OsAlba4 | NP_001049072.1 | MDRYQRVEKPRPEAAVISENEIRITTQGLIRNYVTYATSLLQEKRVKEIVLKAMGQAISKTVAIAEIIKKRIPGLHQDTSISSVSITDVWEPIEEGLVPLEMTRHVSMISISLSPKELNKSSAGYQAPLHAEPIKPQRYQQTQQYQQQQHQSRPSQVQTDSYGRGRGRGRGRGRGWGGRGGYGGGYGGYDNNQGGYGGYGHQGGYGHQGGYGNQGGYGHNQGGYGGYGYNQGGYGGYENGGWNYNRNRGGGGGGGRGRGNWGYGGPGGYERSGPAYERGGRGGGSPGGRGYARGRGRMGGGRGRGNQNY | |
| OsAlba5 | CAD41015.3 | MDRYQRVEKPREEAAIGANEIRITAQGRTRNYITYALALLQDNATDEIVIKAMGRAINKTVAIVELLKRRIVGLHQNTSIESIDITDTWEPLEEGLNTLETTRHVSLITITLSKKELDTSSPGYQPPIPADQVRPPTDFDQEAEAVPSGRGRGRGRRGRGRGRGFNNEDYDDEHGDAEVPQPQGYRGRGRGRGRRGSFGPGRGYGGDGFAMEEAGGYDDGEPNAPPMQGHEGGRGRGRGRGRGCGRGRGGGRGRGPPPPPQE | |
| OsAlba6 | NP_001058008.1 | MEEVTEAVSNLSITEPHKKNRIQVSNTKKPLFFYVNLAKRYMQQHNEVELSALGMAIATVVTVAEILKNNGLAVEKKIMTSTVDVKDDSRSRPMQKAKIEILLGKTEKFDELMAAAAEEREAAAAAEGEEQG | |
| OsAlba7 | EEE70143.1 | MRRRSLGVGGGGGGAEFAFADGNDTYRLNDFGFLHYAVVALGTPNVTFLVALDTGSDLFWVPCDCLKCAPFQSPNYGSLKFDVYSPAQSTTSRKVPCSSNLCDLQNACRSKSNSCPYSIQYLSDNTSSSGVLVEDVLYLTSDSAQSKIVTAPIMFGCGQVQTGSFLGSAAPNGLLGLGMDSKSVPSLLASKGLAANSFSMCFGDDGHGRINFGDTGSSDQKETPLNVYKQNPYYNITITGITVGSKSISTEFSAIVDSGTSFTALSDPMYTQITSSFDAQIRSSRNMLDSSMPFEFCYSVSANGIVHPNVSLTAKGGSIFPVNDPIITITDNAFNPVGYCLAIMKSEGVNLIGGYNFDESSRLPVNPSPSAVPSKPGLGPSSYTPEAAKGALPNGTQLRRGGMDRYQRVEKPREEAPIKENEIRITTQGRMRNYITYATTLLQDKGSDEVVFKAMGRAINKTVMIAELIKRRIVGLHQNTTTGSTDITDMWEPLEEGLLPLETTRHVSMITITLSKKELDTSSIGYQSPLPADKVKPLVEYENEEDAPSPAGRGRGRGGQGRGRGRGRGTRGNGYMDYADGGWEDDHAPPAYAGNGYTRGRGRGFRGRGRRGGGYGAQPDYQQDGGYYDEAPVHAPPRGRGRGRGRGRGPVRGRGRGGNVNGVMHATAVGA | |
| OsAlba8 | AAX92995.1 | MDRYQRVEKPRNETPISENEIRITAQGRMRNYISYGMSLLEENGHDEIVIKAMGRAINKTVMVVELIKRRIGGLHQITSTESIDITDTWEPLEEGLLPLETTRHVSMIAITLSKKALDTLSPGYQPPIPAEEVRPAFDYEHEESFPTNRGRGRGGGRRGRGRAMSNGPPAYDYGEEWEEEGDYYNYRGRGRGRFRGRGRGRGRGGYYGGGRRGGYGYDYGYGGRGDYYEDQGEYFEEPEDYPPPGRGRGRGRRGGGPGPFRGRGRGRGRF | |
| OsAlba9 | EEC69301.1 | MQAVREEEEQVVEEVVRAGAVAEEEEGPEEKEVAMVGEEMAEAEHDEEEAEAGASAKKNRIQVSTNKKPLYFYVNLAKRYMQNYDEVELSALGMAIGTVVTVAEILKNNGLATEKKILTSTIGTKDESKGRLVRKAKIEILLCKSENFNSIMSSKKSDRPKSAEEEIKV | |
| ZmAlba1 | DAA48353.1 | MMLDQGVSGFLLRLLEFKDKGSDEVVLKAMGRAINKIVMTVELIKISTGRGWDLKGQPVLLFYTSYPAGTEMLTNTAKLYKAALGNCFKTYHIGC | |
| ZmAlba2 | DAA48594.1 | MGVFRCSGGWPAGGGGVQRSLGHQTQSQEGLQTGKGPGTPPQGGHRTAAATETTHLEALRTVAAAGTLPQAVRQRAARWKLKASAPARTADKGSDEVVFKAMGRAINKTVMVVELIKRRIVGLHQNTTTGSTDITDMWEPLEEGLLLLETTRLVSMITITLSKKEVDTSSIGYQSPLPPDEVKPLVKYDNDEGIMHTHLVARGRGRSGRGCGQGQGQRNGFNEFADVGWEDDHAPAYMGNGYACGRGRSFRGRGRRGGYNNQPEYQQDGGYYEEAPVHAPALGGFSPFVMELKPASHRSGNLFLVVNIYMVYPVVLGTSNFVHIEPVRMTSSLKVIEMLQSSNVIQHVPCAAIPALISCAGNAFLVLAMQTGTGSVCSSSNRIGHRWLVRQSPTLRTLSLGMVAFGFFKATDKYGHVLYLFWEVLPVVLKRLKMF | |
| ZmAlba3 | NP_001140948.1 | MDRYHRVERARNQSTIEENEIRITAQGLIRNYVSYATFLLQERRIKEIVLKAMGQAISKSVAVAEIIKKRAPELHQDTNISSVSITDVWEPIEEGLVPLEMTRHVSMISITLSPRELDKHTPGYQAPAYVEHPRQQQVPPLQRQPRRPSGQQFQQLEYEDSYARGRGRGRGRGRGRGWARGGYGGYGNNQGGYNQGGGYYDNQGGYGGYDDQGGYGGGYGYNQGRYGNYQENGGYNRGRGGGMRGRGNWGYRGGYEGGRGGGYEGGRVGGGYEGGRGGGYEGGRVGGGYEGGRGGGGPGGRGYGGRGRGRMGGGGRAN | |
| ZmAlba4 | NP_001140391.1 | MDRYQRVEKPREESPIGANEIRITAQGRPRNYITYALALLQDNATDDIVIKAMGRAINKTVAIVELLKRRIAGLHQNTSIESIDITDTWEPLEEGLVTLETIRHVSLITIKLSKKELDTSSPGYQPPIPADQVRPAADFDQDAEAVPSGRGRGRGRRSRGRGRGRGFSSGVDYDDEIGEPEEAPRGYRGRGRGRGGRGSFAVGRGYGGDNYAMEEAGRYDDDGYNAPPMQRYEGGRGRGRGRGRGRGRGRQGHGPPPQQ | |
| ZmAlba5 | NP_001146602.1 | MDRYQRVEKPRNDTPISQNEIRITTQGRMRNYISYGMSLLEENGHDEISIKAMGRAINKTVMVVELIKRRVGGLHQNTATESVDITDTWEPLEEGLLPLETTRHVSMITVTLSKNPLDTSSPGYQPPIPAEEVKPAFDYDHEESYPTGRGRGRGGGRRGRGRGMSNGPPPPAYGYNEEWEEEGDYYNRGRGRGRLRGRGGRGRGGYYGGGRRGGYGYDYGYAPRGGYYEEQDEYYDEPEEYAPPPGRGRGRGRRGGMPWRGRGGRGPPRGGRGSYY | |
| ZmAlba6 | NP_001141319.1 | MVVEEIAEGVKNLTVTGDAAASGGEGQRRGGGGSSNRIQVSNTKKPLFFYVNLAKRYMQQHEDVELSALGMAIATVVTVAEILKNNGFAVEKKIRTSTVEINDESRGRPFQKAKIEIILGKSDKFDELMAAAAEERGEVEDGEEQA | |
| ZmAlba7 | XP_008676084.1 | MASLYEILLLLCNILLLCCVPDKLHHNISYFKDKGSDEVVFKAMGRAINKTVMVVELIKRRIVGLHQNTTTGSTDITDMWEPLEEGLLPLETTRHVSMITITLSKKELDTSSIGYAYCTLLSAAFNDFLCQFVLTKFYGFLCHELTSRYVKSVLTIKGAV | |
| ZmAlba8 | AFW60929.1 | MDRYQRVEKPRNDTPISQNEIRITTQGRMRNYISYGMSLLEENGHDEISIKAMGQAINKTVMVVELIKRRVGGLHQNSATESVDITDTWEPLEEGLLPLETTRHVSMITVTLSKKPLDTSSPGYQPPIPAEEVKPAFDYDHEDSYPTGRGRGRGGGRRGRGRGMSNGPSPPAYGYNEEWGEEGDYYNRGRGRGRSRGRGRVRGGYYGGGRRGGYSYGYGARGGYYEEQDEYYDDPEEYAPPGRRGRGRGRRGMPWRGRGVYY | |
| ZmAlba9 | NP_001143331.1 | MDEVTEAVNNLNISGVGAAGVAGAEGHKKNRIQVSNTKKPLFFYVNLAKRYMQLHNEVELSALGMAIATVVTVAEILKNNGLAVAKSIMTSTVDIKDETRTRPIQKAKIEILLGKTEKFDELMAAAAEEREANGAEEQS | |
| ZmAlba10 | XP_008644524.1 | MDRYQRVERPRNESTIEENEIRITAQGLIRNYVSYATSLLQERRIKEIVLKAMGQAISKSVAVAEIIKKRIPGLHQDTNISSVSITDVWEPIEEGLVPLEMTRHVSMISITLSPRELDKHTPGYQAPVYIEQPRQQQVPPPQRQPRRPPAQFQQLEYEDSYARGRGRGRGRGRGWGRGGYGGYGGYGNNQGGYHQGGGYYDNQGGYNGYDNQGGYSGYDNQGGYGGGYGYNQGRYGNYQENGGYNRGRGSGMRGRGNWGYRGGYEGGRGGGYGGGRGGGYEGGRGGGYEGGRGGGYEGGRVYEGGRGGGYEGGRGGGYEGGRGGGYEGGRGGSAQGGRGYGGRGRGRMGGRGRGN | |
| ZmAlba11 | ACN31274.1 | MVEEEITEGLKNLTVTGDAAASGGEGQRRGGGISSNRIQVSNTKKPLFFYVNLAKRYMQQHGDVELSALGMAIATVVTVAEILKNNGFAVEKKIRTSTVDINDESRGRPFQKAKIEIILGKSDRFNELMAAAAEERGEVEEGEEQA | |
| ZmAlba12 | AFW81235.1 | MGRAINKTVMVVELIKRRIVGLHQNTTTGSTDITDMWEPLEEGLLPLETTRHVSMITITLSKKELDTSSIGKRMDRALKDLKEKQNNKKESNIPDVYPFQNHSWQYHFCYNILYVEIKFVVNCFLTCSVAADIQTMLEETQLKFEEERQNLLKVLSNTSKEHIRAGAGHAHPIPAEAHVRLRHLHVCRDGEGHPEQGNPHFVCDVRVLVKPYKEKGKVPGRFRKLQHAHHGGAEFAGCASPTGLLDSRDPYALLLLSGAQVFSWA | |
| ZmAlba13 | XP_008656129.1 | MSGDTAAAVQAGGDAQQAVGRNRIQVSSSKKPLFFYVNLAKRYMQHHDDDVELSALGLAISTAVTVAEILKNNGLAVEKKVRTSTVDIKNEISTRSIQKAKIEIVLGKTNKFDELMAANDGDGTAGDGVGKNDKFDELMAANDGDGTAGGGNKQRFVIDFPTIFISSPLLDRSS | |
| ZmAlba14 | AFW87068.1 | MPRVRVRVSVVAEMEELTEGVNNLAITEPQMKNKIQVSNTKKPLFFYVNLAKRYMQQHEEVELSTLGMTIATMVTVAEILKNNRLVVEKKIMTSTADVKDDSRAHPIQKAKIEIVLGKDREI | |
| ZmAlba15 | NP_001132775.1 | MDRYQRVEKPPPEPAAIGENEVRITTQGLVRNYVTYATSLVQEKQVKEIVLKAMGQAISKAVAIAEIIKKRIPGLHQDTIISSVSITDVWEPIEEGLVPLEMTRHVSMISISLSPKEHNKNSPGYQAPLHSDLKPQRYQQPQQYQQYQPRQHQVQTDSYGRGRGRSRGRGRGWGTRGGYGGGYGGYEYDNNQVGYGGYEHHGGYGHQGGYGNQGGYGHNQGGYGHDQGGYGGYGYNQGGYGGYENGGWNYNRNRGGGGRGRGNWGYGGPGYDRGGRGAGGPGGRGYVRGRGRMGGGRGRGNQNYY | |
| ZmAlba16 | XP_008662548.1 | MQAMRPAAEGEAQEQAVRAEADEVKREVAKAHEEEEAVPEEKDVAVVGEEAEAEAETETEGEAEAEVEVEAEVEVEAEAGASSAKKNRIQVSTNKKPLYFYVNLAKRYMQNYDEVELSALGMAIGTVVTVAEILKNNGLATEKKILTSTIGTKDEAKGRLVRKAKIEILLCKSENFNSIMSSKKSERPKPPAEEEIKV | |
| ZmAlba17 | AFW57019.1 | MGVFRCSAGWPAGGGSAQRSLGHQTQSQEGLQTGKGPGTPPRGGHRTAATTEMTHLEDLRTVAAAGTLPQAVRQRAARQKLTASAPARTVDKGSYEVVFKAMGRAINKTVMVVELIKRRIVGLHQNTTTGSTDMWEPLEECLLLLETTRHVSMITITLSKKEVDTSSIGYQSPLPADEVKPLVKCDTDEDAHSPGGRGRGRSGRGRGQGRGRGGRGNGFNEFADAGWEDDHAPAYMGNGYARGRGRSFRGRGRRGGYNNQPEYQQDGGY | |
| ZmAlba18 | AFW58594.1 | MGQWGTDKGSDEVVFKAMGRTINKTFMVVELIKRRIVGLHQNTTTGSTGITDMWEPLEEGLLPLRQQDMSLCLYDHYNSFKEGAGHIIYQITACNLSRFVEPTPIQSQGWPMTLKGRDLIDIAQTGSGKTLSYLLPRLVHVGAQPRLEFKSGKSPIMAATDVAARGLDLKLTP | |
| SbAlba1 | XP_002463920.1 | MDRYQRVERPRNESTIEENEIRITAQGLIRNYVSYATSLLQERRIKEIVLKAMGQAISKSVAVAEIIKKRIPGLHQDTNISSVSITDVWEPIEEGLVPLEMTRHVSMISITLSPRELDKQTPGYQAPVYVEQPRQQQGPPLQRQPRRPPGQQFQQLEYEDSYARGRGRGRGRGRGRGWGWGRGGYGGYGGYGNNQGGYNQGGGYYDNQGGYGGYDNQGGYGGGYGYNQGRYGNYQENGGYNRGRGGGMRGRGNWGYRGGYDGGRGGGYEGGRGGGYEGGRGGGYEGGRGGGYEGGRGGGYEGGRGGGYEGGRGGGYEGGRGGGAPGGRGYGGRGRGRMGGRGRGN | |
| SbAlba2 | KXG40112.1 | MDRYHRVEKPRPEAAAISENEIRITTQGLIRNYVTYATSLVQEKRVKEIVLKAMGQAISKTVAIAEIIKKRIPGLHQDTIISSVSITDVWEPIEEGLVPLEMTRHVSMISISLSPKELNKNSPGYQAPLHLDLKPQRYQQPQQYQQHQPRQNPIQTDSYGRGRGRGRGRGRGWGSRGGYGGGYGGYEYDNQGGYGGYGHQGGYGHQGGYGNQGGYGHNQDGYGGYGYNQGGYGGYENGGWNYNRNRGGGGGGGRGRGNWGYGGPGYDRGGRGAGGPGGRGYVRGRGRMGGGRGRGNQNY | |
| SbAlba3 | XP_002462756.1 | MDRYQRVEKPREEAPIKENEIRITTQGRMRNYITYATALLQDKGSDEVVFKAMGRAINKTVMIAELIKRRIVGLHQNTTTGSTDITDMWEPLEEGLLPLETTRHVSMITITLSKKELDTSSIGYQSPLPADEVKPLVEYDNDEDAHSPGGRGRGRGGRGRGRGRGRGRGGRGNGYNDYADVGWEDDHAPAYMGNGYPRGRGRGFRGRGRRGGYNGQPDYQQDGGYYEEAPVHAPARGRGRGRGRGPSRGRGRGGNANGVMHAAAPGA | |
| SbAlba4 | XP_002455102.1 | MVVEEITEGVKNLAVAGDAAAASGGEGQRRGGGGSSNRIQVSNTKKPLFFYVNLAKRYMQQHGDVELSALGMAIATVVTVAEILKNNGFAVEKKIRTSTVEINDESRGRPFQKAKIEIILGKSDKFDELMAAAAEERGEVEDGEEQA | |
| SbAlba5 | XP_002453505.1 | MEEVTEAVNNLSISGGGATAGAGAGAEGHKKNRIQVSNTKKPLFFYVNLAKRYMQLHNEVELSALGMAIATVVTVAEILKNNGLAVEKKIMTSTVDVKDETRPRPIQKAKIEILLGKTDKFDELMAAAAEEREANEAEEQS | |
| SbAlba6 | XP_002449052.1 | MDRYHRVEKPRNDTPISQNEIRITTQGRMRNYISYGMSLLEENGHDEINIKAMGRAINKTVMVVELIKRRVGGLHQNTATESVDITDTWEPLEEGLLPLETTRHVSMITVTLSKKPLDTSSPGYQPPIPAEEVKPAFDYDHEESYPTGRGRGRFGGRRGRGRGMSNGPPPPAYGYNDEWEEDGDYYNRGRGRGRSRGRGGRGRGGYYGGGRRGGYGYDYGYGGRGGYYEEQDEYYDEPEEYAPPPGRGRGRGRRGMPWRGRGGRGPPRGGRGGYY | |
| SbAlba7 | XP_002447839.1 | MDRYHRVEKPREEEAPIGANEIRITAQGRPRNYITYALALLQDNATDDIVIKAMGRAINKTVVIVELLKRRIAGLHQNTSIESINITDTWEPLEEGLVTLETVRHVSLITIKLSKKELDTSSPGYQPPIPADQVRPAAEFDQDAEAVPSGRGRGRGRRGRGRGRGRGFSNGGVDYHDEFGEPEEAPRGYGGRGRGRGGRGSFAPGRGYGGDNYAMEEAGGYDDGYNAPPMQGYEGGRGRGRGRGRGRGRGRGSQGQGPPQQ | |
| SbAlba8 | XP_002443196.1 | MQAVRPAAEGEEAQAQEQAVREEVAEVKREVAKAHEEEAAPEEKDVAVVGEEADAEAEAEmplptpatsaAETEGEAEAEAEAGASAKKNRIQVSTNKKPLYFYVNLAKRYMQNYDEVELSALGMAIGTVVTVAEILKNNGLATEKKILTSTIGTKDESKGRLVRKAKIEILLCKSENFNSIMSSKKSDR  PKSAEEEIKV | |
| SbAlba9 | XP_002440693.1 | MSGGDMAAQAGGDAQQQQQQAVGGNRIQVSSSKKPLFFYVNLAKKYMQQHGDVELSALGLAISTVVTIAEILKNNGLAVEKKIRTSTVEIIDETKARPIQKAKIEIVLGKTDKFEELMAANVGDANAGDGEEQT | |
| SbAlba10 | XP_002437246.1 | MEEVTEGVNNLAITEPHKKNRIQVSNTKKPLFFYVNLAKRYMQQHEEVELSALGMAIATVVTVAEILKNNGLAVEKKIMTSTVDVKDDTRARPIQKAKIEIVLGKTDKFDELMAAADAEREAAEAEEQS | |

| **Table S2.** The primer sequences used for subcellular localization analysis | | | | | | | | | | |  |  |  |
| --- | --- | --- | --- | --- | --- | --- | --- | --- | --- | --- | --- | --- | --- |
| **Gene Name** | | **Primer sequence (5' to 3')** | | | | | | | | |  |  |  |
| *SlAlba4* | | GCAGCCCGGGGGATCCAAAACTAAAAGTAGAAGAAAAATGGAAGGAATCACAGACGG | | | | | | | | |  |  |  |
|  | | CCATTCTAGAACTAGTGTTCTGCTGCTCCTCATTGTCC | | | | | | | | |  |  |  |
| *SlAlba5* | | GCAGCCCGGGGGATCCAAAACTAAAAGTAGAAGAAAAATGGAAGAGTTAGCAGAAGGAGTG | | | | | | | | |  |  |  |
|  | | CCATTCTAGAACTAGTACTCTGTTCCTGAATATTGTTTCC | | | | | | | | |  |  |  |
| *SlAlba6* | | GCAGCCCGGGGGATCCAAAACTAAAAGTAGAAGAAAAATGGATAGGTACCAAAAAGTAGAGA | | | | | | | | |  |  |  |
|  | | CCATTCTAGAACTAGTAGCCTGGTTGTTATTGCCAC | | | | | | | | |  |  |  |
|  | |  | | | | | |  | | |  |  |  |
| **Table S3.** The primer sequences of *SlAlba* genes used for qRT-PCR analysis | | | | | | | | | | | | | |
|  | |  | | | | | |  | | | | | |
| **Gene Name** | | **Forward Primer** | | | | | | **Reverse Primer** | | | | | |
| *SlAlba1* | | CGGTTGTCACAGTTGCTGAG | | | | | | TGCTTTCTGAACCATGCGGC | | | | | |
| *SlAlba2* | | GGGTAGAGCAATTAGCAAAACA | | | | | | AGTCCTTCTTCTAAAGGTTCC | | | | | |
| *SlAlba3* | | CGAGACCACCAGGCATGTAT | | | | | | TCTGTTGACACTTTCACCTGGT | | | | | |
| *SlAlba4* | | GTGGAACTCTCTGCTCTTGG | | | | | | ATGTCCACGGTGAGTGTCCT | | | | | |
| *SlAlba5* | | AGAGTTAGCAGAAGGAGTGAG | | | | | | CCCAAAGCTGAAAGCTCCAC | | | | | |
| *SlAlba6* | | GCAATGGGTCAGGCAATAAG | | | | | | AGGCCCTCTTCAATAGGTTC | | | | | |
| *SlAlba7* | | GGTCAGGCAATTAGCAAAGCA | | | | | | AGACCCTCTTCAATTGGCTC | | | | | |
| *SlAlba8* | | CTGAGTTGAATCAAACCTCTCC | | | | | | CCTCGACCACGGACATCATA | | | | | |
| **Table S4.** Sequence identity among 8 tomato Alba proteins | | | | | | | | | | | | |  |
|  | SlAlba1 | | SlAlba2 | SlAlba3 | SlAlba4 | SlAlba5 | SlAlba6 | | SlAlba7 | SlAlba8 | |  |  |
| SlAlba1 | 100% | |  |  |  |  |  | |  |  | |  |  |
| SlAlba2 | 15.02% | | 100% |  |  |  |  | |  |  | |  |  |
| SlAlba3 | 14.50% | | 62.23% | 100% |  |  |  | |  |  | |  |  |
| SlAlba4 | 59.37% | | 25.78% | 23.43% | 100% |  |  | |  |  | |  |  |
| SlAlba5 | 60.62% | | 22.04% | 17.32% | 68.50% | 100% |  | |  |  | |  |  |
| SlAlba6 | 18.65% | | 46.78% | 47.24% | 23.43% | 19.68% | 100% | |  |  | |  |  |
| SlAlba7 | 17.09% | | 47.21% | 48.81% | 23.43% | 22.04% | 67.55% | | 100% |  | |  |  |
| SlAlba8 | 19.17% | | 49.78% | 52.36% | 27.34% | 24.40% | 61.83% | | 53.79% | 100% | |  |  |

| **Table S5.** List of *cis* elements in the promoter regions tomato *Alba* genes | | | | | | | |
| --- | --- | --- | --- | --- | --- | --- | --- |
| **Gene** | **Promoter** | **Consensus sequence** | **Position** | **Strand** | **organism** | **Function** | |
| *SlAlba1* | TCA-element | CCATCTTTTT | 836 | - | *Nicotiana tabacum* | | cis-acting element involved in salicylic acid responsiveness |
|  | TCA-element | CCATCTTTTT | 1199 | + | *Nicotiana tabacum* | | cis-acting element involved in salicylic acid responsiveness |
|  | TC-rich repeats | GTTTTCTTAC | 748 | + | *Nicotiana tabacum* | | cis-acting element involved in defense and stress responsiveness |
|  | ABRE | ACGTG | 509 | + | *Arabidopsis thaliana* | | cis-acting element involved in the abscisic acid responsiveness |
| *SlAlba2* | ABRE | ACGTG | 423 | - | *Arabidopsis thaliana* | | cis-acting element involved in the abscisic acid responsiveness |
|  | ABRE | CACGTG | 1257 | - | *Arabidopsis thaliana* | | cis-acting element involved in the abscisic acid responsiveness |
|  | CGTCA-motif | CGTCA | 619 | - | *Hordeum vulgare* | | cis-acting regulatory element involved in the MeJA-responsiveness |
|  | AACA_motif | TAACAAACTCCA | 3 | - | *Oryza sativa* | | involved in endosperm-specific negative expression |
|  | ARE | AAACCA | 645 | + | *Zea mays* | | cis-acting regulatory element essential for the anaerobic induction |
| *SlAlba3* | CGTCA-motif | CGTCA | 693 | - | *Hordeum vulgare* | | cis-acting regulatory element involved in the MeJA-responsiveness |
|  | CAT-box | GCCACT | 93 | - | *Arabidopsis thaliana* | | cis-acting regulatory element related to meristem expression |
|  | O2-site | GATGA(C/T)(A/G)TG(A/G) | 561 | - | *Zea mays* | | cis-acting regulatory element involved in zein metabolism regulation |
|  | TATC-box | TATCCCA | 895 | + | *Oryza sativa* | | cis-acting element involved in gibberellin-responsiveness |
| *SlAlba4* | TGA-element | AACGAC | 1465 | - | *Brassica oleracea* | | auxin-responsive element |
|  | CGTCA-motif | CGTCA | 302 | - | *Hordeum vulgare* | | cis-acting regulatory element involved in the MeJA-responsiveness |
|  | TC-rich repeats | ATTCTCTAAC | 1289 | + | *Nicotiana tabacum* | | cis-acting element involved in defense and stress responsiveness |
|  | WUN-motif | AAATTTCCT | 1135 | + | *Brassica oleracea* | | wound-responsive element |
|  | ARE | AAACCA | 134 | + | *Zea mays* | | cis-acting regulatory element essential for the anaerobic induction |
|  | ARE | AAACCA | 1489 | + | *Zea mays* | | cis-acting regulatory element essential for the anaerobic induction |
| *SlAlba5* | CGTCA-motif | CGTCA | 1364 | - | *Hordeum vulgare* | | cis-acting regulatory element involved in the MeJA-responsiveness |
|  | O2-site | GATGA(C/T)(A/G)TG(A/G) | 419 | + | *Zea mays* | | cis-acting regulatory element involved in zein metabolism regulation |
|  | HD-Zip 1 | CAAT(A/T)ATTG | 727 | + | *Arabidopsis thaliana* | | element involved in differentiation of the palisade mesophyll cells |
| *SlAlba6* | LTR | CCGAAA | 1436 | - | *Hordeum vulgare* | | cis-acting element involved in low-temperature responsiveness |
|  | ARE | AAACCA | 741 | - | *Zea mays* | | cis-acting regulatory element essential for the anaerobic induction |
|  | TC-rich repeats | ATTCTCTAAC | 1107 | - | *Nicotiana tabacum* | | cis-acting element involved in defense and stress responsiveness |
|  | AuxRR-core | GGTCCAT | 145 | - | *Nicotiana tabacum* | | cis-acting regulatory element involved in auxin responsiveness |
|  | TC-rich repeats | GTTTTCTTAC | 10 | + | *Nicotiana tabacum* | | cis-acting element involved in defense and stress responsiveness |
| *SlAlba7* | MBS | CAACTG | 353 | - | *Arabidopsis thaliana* | | MYB binding site involved in drought-inducibility |
|  | MBS | CAACTG | 415 | - | *Arabidopsis thaliana* | | MYB binding site involved in drought-inducibility |
|  | TC-rich repeats | GTTTTCTTAC | 709 | - | *Nicotiana tabacum* | | cis-acting element involved in defense and stress responsiveness |
|  | MBS | CAACTG | 343 | + | *Arabidopsis thaliana* | | MYB binding site involved in drought-inducibility |
|  | TATC-box | TATCCCA | 1159 | + | *Oryza sativa* | | cis-acting element involved in gibberellin-responsiveness |
| *SlAlba8* | LTR | CCGAAA | 981 | - | *Hordeum vulgare* | | cis-acting element involved in low-temperature responsiveness |
|  | ARE | AAACCA | 810 | - | *Zea mays* | | cis-acting regulatory element essential for the anaerobic induction |
|  | ARE | AAACCA | 1419 | - | *Zea mays* | | cis-acting regulatory element essential for the anaerobic induction |
|  | GARE-motif | TCTGTTG | 1414 | + | *Brassica oleracea* | | gibberellin-responsive element |
|  | ARE | AAACCA | 63 | + | *Zea mays* | | cis-acting regulatory element essential for the anaerobic induction |
|  |  |  |  |  |  | |  |

**Table S7.** Templates used for 3D structure modeling of SlAlba proteins

| **No.** | **Protein** | **PDB Hit** | **IDEN** | **Cov** | **Z-score** |
| --- | --- | --- | --- | --- | --- |
| 1 | SlAlba1 | 2q3vB | 0.74 | 0.53 | 2.34 |
| 2 | SlAlba2 | 6ahrF | 0.34 | 0.53 | 1.92 |
| 3 | SlAlba3 | 6ahrF | 0.33 | 0.49 | 1.96 |
| 4 | SlAlba4 | 2q3vB | 0.75 | 0.77 | 2.00 |
| 5 | SlAlba5 | 2q3vB | 0.73 | 0.78 | 1.93 |
| 6 | SlAlba6 | 6ahrF | 0.31 | 0.47 | 1.80 |
| 7 | SlAlba7 | 5fmwA | 0.08 | 0.99 | 1.11 |
| 8 | SlAlba8 | 6ahrF | 0.32 | 0.42 | 1.70 |

^IDEN- percentage sequence identity of template in the structurally aligned region. Cov- coverage of threading alignment by TM align. Z-score^ **^-^** ^alignment with a normalized z-score larger than 1 indicates good alignment.^

**Table S8.** Secondary structural components in SlAlba proteins

| **No.** | **Protein name** | **α- Helix** | **β- Sheets** | **Coils** |
| --- | --- | --- | --- | --- |
| 1 | SlAlba1 | 5 | 6 | 10 |
| 2 | SlAlba2 | 3 | 5 | 7 |
| 3 | SlAlba3 | 2 | 6 | 9 |
| 4 | SlAlba4 | 4 | 6 | 9 |
| 5 | SlAlba5 | 5 | 4 | 9 |
| 6 | SlAlba6 | 2 | 5 | 8 |
| 7 | SlAlba7 | 3 | 6 | 10 |
| 8 | SlAlba8 | 3 | 6 | 10 |

**Table S9.** Secondary structure prediction for SlAlba proteins by I-TASSER.

| **No.** | **GI Number** | **Type** | **TM-Score** | **RMSD** |
| --- | --- | --- | --- | --- |
| 1 | 1104569164 | SlAlba1 | 0.36+-0.12 | 12.6+-4.3 |
| 2 | 723693946 | SlAlba2 | 0.35+-0.12 | 13.5+-4.0 |
| 3 | 460385381 | SlAlba3 | 0.37 ± 0.13 | 13.1 ± 4.1 |
| 4 | 460392354 | SlAlba4 | 0.62 ± 0.14 | 5.9 ± 3.6 |
| 5 | 460392188 | SlAlba5 | 0.61 ± 0.14 | 6.1 ± 3.8 |
| 6 | 460393594 | SlAlba6 | 0.32 ± 0.10 | 14.8 ± 3.6 |
| 7 | 1104635736 | SlAlba7 | 0.56 ± 0.15 | 9.5 ± 4.6 |
| 8 | 723732812 | SlAlba8 | 0.26 ± 0.08 | 17.0 ± 2.8 |

^GI number, GenInfo Identifier number; TM-score,^ ^Template modeling score; RMSD: root-mean-square deviation between residues structurally aligned by TM-align.^

**Table S10.** Parameters for 3D structure modeling of SlAlba proteins.

| **No.** | **Type** | **GI Number** | **C-Score** | **No. of Decoys** | **Cluster Density** |
| --- | --- | --- | --- | --- | --- |
| 1 | SlAlba1 | 1104569164 | -3.16 | 1608 | 0.0197 |
| 2 | SlAlba2 | 723693946 | -3.30 | 1231 | 0.0141 |
| 3 | SlAlba3 | 460385381 | -3.06 | 615 | 0.0187 |
| 4 | SlAlba4 | 460392354 | -0.70 | 7038 | 0.1925 |
| 5 | SlAlba5 | 460392188 | -0.83 | 7083 | 0.1685 |
| 6 | SlAlba6 | 460393594 | -3.65 | 621 | 0.0108 |
| 7 | SlAlba7 | 1104635736 | -1.20 | 600 | 0.1370 |
| 8 | SlAlba8 | 723732812 | -4.30 | 418 | 0.0056 |

^GI number, GenInfo Identifier number; C-Score, confident score.^

**Table S12.** Gene Ontology (GO) annotation for SlAlba proteins

| **Name** | **Molecular Process** | **Biological Process** | **Cellular component** |
| --- | --- | --- | --- |
| SlAlba1 | metal ion binding, protein binding, | rRNA methylation, carbohydrate metabolic process, anaerobic glutamate catabolic process | obsolete cytoplasmic part |
| SlAlba2 | RNA, DNA Binding | fatty acid biosynthetic process | Chromosome, cytoplasm |
| SlAlba3 | flavin adenine dinucleotide binding, nucleotide binding | response to bacterium | Cytoplasm, extracellular space |
| SlAlba4 | RNA, DNA Binding | chromosome condensation | Chromosome, cytosol |
| SlAlba5 | RNA, DNA Binding | chromosome condensation | Chromosome, cytosol |
| SlAlba6 | flavin adenine dinucleotide binding, sequence-specific DNA binding | cellular response to stress, carbohydrate metabolic process, L-phenylalanine catabolic process, respiratory electron transport chain, | mitochondrial respiratory chain complex II, succinate dehydrogenase complex (ubiquinone) |
| SlAlba7 | protein binding, channel activity, ion binding | Cytolysis, protein complex oligomerization | membrane attack complex, extracellular space, |
| SlAlba8 | flavin adenine dinucleotide binding, nucleotide binding, RNA cap binding, protein binding | glutamine metabolic process, regulation of innate immune response, peptidoglycan biosynthetic process, cell division, obsolete oxidation-reduction process, membrane protein ectodomain proteolysis | endoplasmic reticulum membrane, cytosol |

| **Table S14. Information of samples used for RNA seq analysis** | | | |  |  |  |
| --- | --- | --- | --- | --- | --- | --- |
| Run | Project | Sample_ID | Tissue | Develoment_stage | Treatment | Species |
| SRR12026415 | PRJNA639840 | S-H-C-1 | leaf | Seedling stage | Control | *Solanum lycopersicum* |
| SRR12026416 | PRJNA639840 | S-H-C-2 | leaf | Seedling stage | Control | *Solanum lycopersicum* |
| SRR12026417 | PRJNA639840 | S-H-C-3 | leaf | Seedling stage | Control | *Solanum lycopersicum* |
| SRR12026418 | PRJNA639840 | S-1 | leaf | Seedling stage | Salinity | *Solanum lycopersicum* |
| SRR12026419 | PRJNA639840 | S-2 | leaf | Seedling stage | Salinity | *Solanum lycopersicum* |
| SRR12026420 | PRJNA639840 | S-3 | leaf | Seedling stage | Salinity | *Solanum lycopersicum* |
| SRR12026424 | PRJNA639840 | H-1 | leaf | Seedling stage | Heat | *Solanum lycopersicum* |
| SRR12026425 | PRJNA639840 | H-2 | leaf | Seedling stage | Heat | *Solanum lycopersicum* |
| SRR12026426 | PRJNA639840 | H-3 | leaf | Seedling stage | Heat | *Solanum lycopersicum* |
| SRR12026421 | PRJNA639840 | S-H-1 | leaf | Seedling stage | Salinity + heat | *Solanum lycopersicum* |
| SRR12026422 | PRJNA639840 | S-H-2 | leaf | Seedling stage | Salinity + heat | *Solanum lycopersicum* |
| SRR12026423 | PRJNA639840 | S-H-3 | leaf | Seedling stage | Salinity + heat | *Solanum lycopersicum* |
| SRR7652567 | PRJNA484882 | DL-C-1 | leaf | Seedling stage | Control 1 | *Solanum lycopersicum* |
| SRR7652566 | PRJNA484882 | DL-C-2 | leaf | Seedling stage | Control 2 | *Solanum lycopersicum* |
| SRR7652565 | PRJNA484882 | DL-C-3 | leaf | Seedling stage | Control 3 | *Solanum lycopersicum* |
| SRR7652569 | PRJNA484882 | D-1 | leaf | Seedling stage | drought 1 | *Solanum lycopersicum* |
| SRR7652568 | PRJNA484882 | D-2 | leaf | Seedling stage | drought 2 | *Solanum lycopersicum* |
| SRR7652563 | PRJNA484882 | D-3 | leaf | Seedling stage | drought 3 | *Solanum lycopersicum* |
| SRR7652564 | PRJNA484882 | L-1 | leaf | Seedling stage | low temperature 1 | *Solanum lycopersicum* |
| SRR7652571 | PRJNA484882 | L-2 | leaf | Seedling stage | low temperature 2 | *Solanum lycopersicum* |
| SRR7652570 | PRJNA484882 | L-3 | leaf | Seedling stage | low temperature 3 | *Solanum lycopersicum* |
| SRR7652562 | PRJNA484882 | L-D-1 | leaf | Seedling stage | low temperature and drought 1 | *Solanum lycopersicum* |
| SRR7652573 | PRJNA484882 | L-D-2 | leaf | Seedling stage | low temperature and drought 2 | *Solanum lycopersicum* |
| SRR7652572 | PRJNA484882 | L-D-3 | leaf | Seedling stage | low temperature and drought 3 | *Solanum lycopersicum* |
| SRR15607684 | PRJNA756379 | E116-1 | inflorescences | Flowering stage | Early_116_1 | *Solanum lycopersicum* |
| SRR15607683 | PRJNA756379 | E116-2 | inflorescences | Flowering stage | Early_116_2 | *Solanum lycopersicum* |
| SRR15607674 | PRJNA756379 | E116-3 | inflorescences | Flowering stage | Early_116_3 | *Solanum lycopersicum* |
| SRR15607673 | PRJNA756379 | E117-1 | inflorescences | Flowering stage | Early_117_1 | *Solanum lycopersicum* |
| SRR15607672 | PRJNA756379 | E117-2 | inflorescences | Flowering stage | Early_117_2 | *Solanum lycopersicum* |
| SRR15607671 | PRJNA756379 | E117-3 | inflorescences | Flowering stage | Early_117_3 | *Solanum lycopersicum* |
| SRR15607670 | PRJNA756379 | M116-1 | inflorescences | Flowering stage | Mid_116_1 | *Solanum lycopersicum* |
| SRR15607669 | PRJNA756379 | M116-2 | inflorescences | Flowering stage | Mid_116_2 | *Solanum lycopersicum* |
| SRR15607668 | PRJNA756379 | M116-3 | inflorescences | Flowering stage | Mid_116_3 | *Solanum lycopersicum* |
| SRR15607667 | PRJNA756379 | M117-1 | inflorescences | Flowering stage | Mid_117_1 | *Solanum lycopersicum* |
| SRR15607682 | PRJNA756379 | M117-2 | inflorescences | Flowering stage | Mid_117_2 | *Solanum lycopersicum* |
| SRR15607681 | PRJNA756379 | M117-3 | inflorescences | Flowering stage | Mid_117_3 | *Solanum lycopersicum* |
| SRR15607680 | PRJNA756379 | L116-1 | inflorescences | Flowering stage | Late_116_1 | *Solanum lycopersicum* |
| SRR15607679 | PRJNA756379 | L116-2 | inflorescences | Flowering stage | Late_116_2 | *Solanum lycopersicum* |
| SRR15607678 | PRJNA756379 | L116-3 | inflorescences | Flowering stage | Late_116_3 | *Solanum lycopersicum* |
| SRR15607677 | PRJNA756379 | L117-1 | inflorescences | Flowering stage | Late_117_1 | *Solanum lycopersicum* |
| SRR15607676 | PRJNA756379 | L117-2 | inflorescences | Flowering stage | Late_117_2 | *Solanum lycopersicum* |
| SRR15607675 | PRJNA756379 | L117-3 | inflorescences | Flowering stage | Late_117_3 | *Solanum lycopersicum* |
| SRR12443441 | PRJNA655574 | GR-1 | fruit | green ripe stage | GR-G-P-biological replicate1 | *Solanum lycopersicum* |
| SRR12443430 | PRJNA655574 | GR-2 | fruit | green ripe stage | GR-G-P-biological replicate2 | *Solanum lycopersicum* |
| SRR12443419 | PRJNA655574 | GR-3 | fruit | green ripe stage | GR-G-P-biological replicate3 | *Solanum lycopersicum* |
| SRR12443437 | PRJNA655574 | HaR-1 | fruit | half ripe stage | bee-H-P-biological replicate1 | *Solanum lycopersicum* |
| SRR12443436 | PRJNA655574 | HaR-2 | fruit | half ripe stage | bee-H-P-biological replicate2 | *Solanum lycopersicum* |
| SRR12443435 | PRJNA655574 | HaR-3 | fruit | half ripe stage | bee-H-P-biological replicate3 | *Solanum lycopersicum* |
| SRR12443404 | PRJNA655574 | HR-1 | fruit | hard ripe stage | B-HD-P-biological replicate1 | *Solanum lycopersicum* |
| SRR12443403 | PRJNA655574 | HR-2 | fruit | hard ripe stage | B-HD-P-biological replicate2 | *Solanum lycopersicum* |
| SRR12443402 | PRJNA655574 | HR-3 | fruit | hard ripe stage | B-HD-P-biological replicate3 | *Solanum lycopersicum* |
| SRR12443371 | PRJNA655574 | FR-1 | fruit | full ripe stage | S-F-P-biological replicate1 | *Solanum lycopersicum* |
| SRR12443370 | PRJNA655574 | FR-2 | fruit | full ripe stage | S-F-P-biological replicate2 | *Solanum lycopersicum* |
| SRR12443369 | PRJNA655574 | FR-3 | fruit | full ripe stage | S-F-P-biological replicate3 | *Solanum lycopersicum* |
