# Supplementary material for: Genome-wide identification and expression profiling of Alba gene family members in response to abiotic stress in tomato (Solanum lycopersicum L.)
Source: BMC Plant Biol. 2021 Nov 12;21:530. doi: 10.1186/s12870-021-03310-0 (PMC8588595; doi:10.1186/s12870-021-03310-0)
Supplement: Supplementary file 1 — Additional file 1: Figure S1. Schematic representation of the exon-intron distribution of SlAlba gene family. Figure S2. Schematic representation of 15 conserved motifs in Alba proteins from tomato, Arabidopsis, and rice as predicted by Multiple Em for Motif Elicitation (MEME) web server. Figure S3. Chromosome distribution of tomato Alba genes. Figure S4. Gene duplication investigation of Alba genes in the tomato genome. Figure S5. Putative cis-acting elements in the upstream of SlAlba genes. Figure S6. The predicted binding of putative patterner ligands to SlAlba proteins. Figure S7. Overview of conserved motifs of Alba proteins from tomato, Arabidopsis and rice determined using MEME web tool. Figure S8. The top 30 enriched GO terms of co-expressed genes with 6 SlAlba genes. Table S1. List of the Alba amino acid sequences used for phylogenetic investigation. Table S2. The primer sequences used for subcellular localization analysis. Table S3. The primer sequences of SlAlba genes used for qRT-PCR analysis. Table S4. Sequence identity among 8 tomato Alba proteins. Table S5. List of cis-elements in the promoter regions tomato Alba genes. Table S6. Prediction of miRNA target sequences in tomato Alba genes. Table S7. Templates used for 3D structure modelling of SlAlba proteins. Table S8. Secondary structural components in SlAlba proteins. Table S9. Secondary structure prediction for SlAlba proteins by I-TASSER. Table S10. Parameters for 3D structure modelling of SlAlba proteins. Table S11. Subcellular localization of SlAlba proteins predicted by in silico analysis. Table S12. Gene Ontology (GO) annotation for SlAlba proteins. Table S13. Annotated pathways of co-expressed genes. Table S14. Information of samples used for RNA seq analysis. [file 12870_2021_3310_MOESM1_ESM.zip › Table S6, S11 & S13 (supplementary material).docx]

| **Table S6.** Prediction of miRNA target sequences in tomato *Alba* genes | | | | | | | | | | | |
| --- | --- | --- | --- | --- | --- | --- | --- | --- | --- | --- | --- |
| **miRNA_Acc.** | **Target_Acc.** | **Expectation** | **UPE$** | **miRNA_start** | **miRNA_end** | **Target_start** | **Target_end** | **miRNA_aligned_fragment** | **Target_aligned_fragment** | **Inhibition** | **Target_Desc.** |
| aly-miR859-5p | *SlAlba1* | 5 | -1 | 1 | 21 | 354 | 374 | UCUCUCCGUUGUAAAAUCAAA | GGUGGAUUUGCGACGGAGAAG | Cleavage | Nucleic acid binding protein |
| ata-miR171a-5p | *SlAlba1* | 4.5 | -1 | 1 | 20 | 482 | 501 | UGGUAUUGUUUCGGCUCAUG | GAUGACACCAAGCAAUACUA | Cleavage | Nucleic acid binding protein |
| ata-miR9863a-5p | *SlAlba1* | 5 | -1 | 1 | 21 | 366 | 386 | UGUUAUGAUCUGCUUCUCAUC | ACGGAGAAGAAGGUUUUAACA | Cleavage | Nucleic acid binding protein |
| ata-miR9863b-5p | *SlAlba1* | 5 | -1 | 1 | 21 | 366 | 386 | UGUUAUGAUCUGCUUCUCAUC | ACGGAGAAGAAGGUUUUAACA | Cleavage | Nucleic acid binding protein |
| ath-miR5640 | *SlAlba1* | 5 | -1 | 1 | 21 | 48 | 68 | UGAGAGAAGGAAUUAGAUUCA | UUAAUUGAAAUUUUUUUUUCA | Cleavage | Nucleic acid binding protein |
| ath-miR8182 | *SlAlba1* | 5 | -1 | 1 | 22 | 189 | 210 | UUGUGUUGCGUUUCUGUUGAUU | GCUCAGAAGAAAAGUAACAGAA | Translation | Nucleic acid binding protein |
| bdi-miR5057 | *SlAlba1* | 5 | -1 | 1 | 21 | 40 | 60 | AAAUUUCAAAUCAUUUUGACA | CCCCAAAUUUAAUUGAAAUUU | Translation | Nucleic acid binding protein |
| bdi-miR5185a-3p | *SlAlba1* | 4.5 | -1 | 1 | 21 | 11 | 31 | UUUGAGAAUUGAACUAGAAGC | UUUUUUAAUUUAAAUUUUAAA | Cleavage | Nucleic acid binding protein |
| bdi-miR5185b-3p | *SlAlba1* | 4.5 | -1 | 1 | 21 | 11 | 31 | UUUGAGAAUUGAACUAGAAGC | UUUUUUAAUUUAAAUUUUAAA | Cleavage | Nucleic acid binding protein |
| bdi-miR7711-5p.1 | *SlAlba1* | 4.5 | -1 | 1 | 24 | 721 | 744 | UACUUAGCCUCUUUGACAAUCUUG | GUUAAAAGUUAGAGAGGCUAAAUA | Cleavage | Nucleic acid binding protein |
| bra-miR5721 | *SlAlba1* | 5 | -1 | 1 | 21 | 676 | 696 | AAAAAUGGAGUGAGAAAUGGA | AUUAUUUCUUAUUAUAUUUGU | Cleavage | Nucleic acid binding protein |
| bra-miR9556-5p | *SlAlba1* | 5 | -1 | 1 | 21 | 544 | 563 | GUCAAUUGGUGAUAGUAGUUC | AGAAUACUAUUACUAAU-GAC | Cleavage | Nucleic acid binding protein |
| cre-miR1160.2 | *SlAlba1* | 5 | -1 | 1 | 21 | 232 | 252 | UGACAAGGAAGCAGAGCGGAU | AACCACUUUUCUUCUAUGUCA | Cleavage | Nucleic acid binding protein |
| csi-miR3949 | *SlAlba1* | 5 | -1 | 1 | 22 | 809 | 830 | UGAUGUUGAGGCAAAAAUGUAG | UUUUUUUUUUGCCUCCAUAUAA | Cleavage | Nucleic acid binding protein |
| csi-miR3950 | *SlAlba1* | 4.5 | -1 | 1 | 21 | 443 | 463 | UUUUUCGGCAACAUGAUUUCU | UGAGAUAGUGUUGACGAAGAG | Cleavage | Nucleic acid binding protein |
| gma-miR1533 | *SlAlba1* | 3.5 | -1 | 1 | 19 | 681 | 699 | AUAAUAAAAAUAAUAAUGA | UUCUUAUUAUAUUUGUUAU | Cleavage | Nucleic acid binding protein |
| gma-miR4351 | *SlAlba1* | 4.5 | -1 | 1 | 22 | 617 | 638 | AUUGGGAUUCAGUUGGAGUUGG | CUCAUUCUUACUGUAUCUCAGU | Cleavage | Nucleic acid binding protein |
| gma-miR5672 | *SlAlba1* | 4 | -1 | 1 | 21 | 701 | 721 | CAUGGUAGUGGAAGAAAUGGA | AUUAUUACUACUACUACUAUG | Cleavage | Nucleic acid binding protein |
| gma-miR5779 | *SlAlba1* | 5 | -1 | 1 | 24 | 288 | 311 | CAAGUCCAAAGUAGGAAUGUUGCA | GUUGAGCUUUCUGCUUUGGGCAUG | Cleavage | Nucleic acid binding protein |
| gma-miR9724 | *SlAlba1* | 5 | -1 | 1 | 22 | 664 | 685 | UAGAGAUAGUGUCAAAAUAGAA | UAUUAUUCUGCUAUUAUUUCUU | Cleavage | Nucleic acid binding protein |
| gma-miR9729 | *SlAlba1* | 5 | -1 | 1 | 24 | 583 | 606 | GUAAUGAGUAGAAACAUUUAGAAG | AGUAGAAAGGAUGCUACUUGUUAU | Cleavage | Nucleic acid binding protein |
| gma-miR9749 | *SlAlba1* | 4.5 | -1 | 1 | 21 | 721 | 741 | UUAGCUUCUUUCACCUUUCCC | GUUAAAAGUUAGAGAGGCUAA | Cleavage | Nucleic acid binding protein |
| gra-miR530a | *SlAlba1* | 4.5 | -1 | 1 | 21 | 150 | 170 | AGGUGCAGAUGCAGUUGCAGG | UCUACACCAGCAUCUGCACCA | Cleavage | Nucleic acid binding protein |
| gra-miR530a | *SlAlba1* | 5 | -1 | 1 | 21 | 105 | 125 | AGGUGCAGAUGCAGUUGCAGG | AUGGCACCUGUAGCUGCACCC | Cleavage | Nucleic acid binding protein |
| gra-miR530b | *SlAlba1* | 5 | -1 | 1 | 21 | 105 | 125 | AGGUGCAGGUGCAGGCGCAGC | AUGGCACCUGUAGCUGCACCC | Cleavage | Nucleic acid binding protein |
| gra-miR7492d | *SlAlba1* | 5 | -1 | 1 | 24 | 21 | 44 | UGGGCUUAGAUUUUUUGCGGCGUU | UAAAUUUUAAAAAGUUUAACCCCA | Cleavage | Nucleic acid binding protein |
| gra-miR7494c | *SlAlba1* | 5 | -1 | 1 | 24 | 803 | 826 | AUGGAGGAAAACAGAGGGAGAAGC | AGGAAUUUUUUUUUUUGCCUCCAU | Cleavage | Nucleic acid binding protein |
| gra-miR7504d | *SlAlba1* | 5 | -1 | 1 | 24 | 45 | 68 | AGGAAAAAAAAUCUGAUUUGUCAU | AAUUUAAUUGAAAUUUUUUUUUCA | Cleavage | Nucleic acid binding protein |
| gra-miR8691 | *SlAlba1* | 5 | -1 | 1 | 24 | 669 | 692 | AGAUGAUGAGAAAGGAAAGUCAAG | UUCUGCUAUUAUUUCUUAUUAUAU | Cleavage | Nucleic acid binding protein |
| gra-miR8756 | *SlAlba1* | 5 | -1 | 1 | 24 | 15 | 38 | UGGACUGUUAAAAUUUUAAUGGCA | UUAAUUUAAAUUUUAAAAAGUUUA | Translation | Nucleic acid binding protein |
| gra-miR8770 | *SlAlba1* | 5 | -1 | 1 | 24 | 685 | 708 | UUGAUGGUGGUAAGAAAUGUGCAU | UAUUAUAUUUGUUAUUAUUAUUAC | Cleavage | Nucleic acid binding protein |
| hme-miR-6305-3p | *SlAlba1* | 5 | -1 | 1 | 22 | 667 | 688 | UGUGAGAAAUUCUAGCAGAUGA | UAUUCUGCUAUUAUUUCUUAUU | Translation | Nucleic acid binding protein |
| hme-miR-6305-3p | *SlAlba1* | 5 | -1 | 1 | 22 | 827 | 848 | UGUGAGAAAUUCUAGCAGAUGA | AUAACUUAUAGAGUUUGUCACA | Cleavage | Nucleic acid binding protein |
| mtr-miR2590h | *SlAlba1* | 4.5 | -1 | 1 | 24 | 233 | 256 | AGAAUGACAUGGCAGAAUAAUCAC | ACCACUUUUCUUCUAUGUCAAUCU | Cleavage | Nucleic acid binding protein |
| mtr-miR2590i | *SlAlba1* | 4.5 | -1 | 1 | 24 | 233 | 256 | AGAAUGACAUGGCAGAAUAAUCAC | ACCACUUUUCUUCUAUGUCAAUCU | Cleavage | Nucleic acid binding protein |
| mtr-miR2592ap | *SlAlba1* | 4.5 | -1 | 1 | 21 | 142 | 162 | AGGCUGGUUUAGAUGAAGGUA | CACCUGUAUCUACACCAGCAU | Cleavage | Nucleic acid binding protein |
| mtr-miR2592aq | *SlAlba1* | 4.5 | -1 | 1 | 21 | 142 | 162 | AGGCUGGUUUAGAUGAAGGUA | CACCUGUAUCUACACCAGCAU | Cleavage | Nucleic acid binding protein |
| mtr-miR2661 | *SlAlba1* | 4.5 | -1 | 1 | 21 | 854 | 874 | UAGGUUUGAGAAAAUGGGCAG | CAGUUCCCUUUUUCAAACUUG | Cleavage | Nucleic acid binding protein |
| mtr-miR2671a | *SlAlba1* | 5 | -1 | 1 | 21 | 364 | 384 | UUAAAAGUUUCGUUUCGGUCC | CGACGGAGAAGAAGGUUUUAA | Cleavage | Nucleic acid binding protein |
| mtr-miR2671b | *SlAlba1* | 5 | -1 | 1 | 21 | 364 | 384 | UUAAAAGUUUCGUUUCGGUCC | CGACGGAGAAGAAGGUUUUAA | Cleavage | Nucleic acid binding protein |
| mtr-miR5257 | *SlAlba1* | 5 | -1 | 1 | 21 | 583 | 603 | ACAAGUAGAACCUUUUUUCUG | AGUAGAAAGGAUGCUACUUGU | Translation | Nucleic acid binding protein |
| mtr-miR5558-3p | *SlAlba1* | 4.5 | -1 | 1 | 21 | 9 | 29 | UAGAUUUAGAAUUAGAAAAGC | CUUUUUUUAAUUUAAAUUUUA | Cleavage | Nucleic acid binding protein |
| mtr-miR7696c-3p | *SlAlba1* | 5 | -1 | 1 | 21 | 413 | 433 | UUUUGAAUUAUGAGAACUUGA | CAAAGGCCGCAUGGUUCAGAA | Cleavage | Nucleic acid binding protein |
| mtr-miR7696d-3p | *SlAlba1* | 5 | -1 | 1 | 21 | 413 | 433 | UUUUGAAUUAUGAGAACUUGA | CAAAGGCCGCAUGGUUCAGAA | Cleavage | Nucleic acid binding protein |
| osa-miR2873a | *SlAlba1* | 5 | -1 | 1 | 24 | 26 | 49 | AAGUUUGGACUUAAAUUUGGUAAC | UUUAAAAAGUUUAACCCCAAAUUU | Translation | Nucleic acid binding protein |
| osa-miR5157a-3p | *SlAlba1* | 5 | -1 | 1 | 24 | 779 | 802 | AGAAGUUGUGGCUAUCAAAAAGUU | UGUUGUUUGUGAGUUGUGACUUUU | Cleavage | Nucleic acid binding protein |
| osa-miR5157b-3p | *SlAlba1* | 5 | -1 | 1 | 24 | 779 | 802 | AGAAGUUGUGGCUAUCAAAAAGUU | UGUUGUUUGUGAGUUGUGACUUUU | Cleavage | Nucleic acid binding protein |
| osa-miR5788 | *SlAlba1* | 4.5 | -1 | 1 | 21 | 832 | 852 | UGGAUGUGACAUACUCUAGUA | UUAUAGAGUUUGUCACAUUUU | Cleavage | Nucleic acid binding protein |
| osa-miR5824 | *SlAlba1* | 5 | -1 | 1 | 24 | 671 | 693 | AGUCUGAUAAGAAGUCAAUGGCGU | CUGCUAUU-AUUUCUUAUUAUAUU | Cleavage | Nucleic acid binding protein |
| osa-miR7693-5p | *SlAlba1* | 5 | -1 | 1 | 24 | 447 | 470 | GUUUCCGCUCUUCAUCGAUGGCUG | AUAGUGUUGACGAAGAGCGAGAAG | Cleavage | Nucleic acid binding protein |
| ppt-miR902k-5p | *SlAlba1* | 5 | -1 | 1 | 21 | 350 | 370 | UAUGUUGCAGAUUCUUCAUUU | GAAUGGUGGAUUUGCGACGGA | Cleavage | Nucleic acid binding protein |
| ptc-miR6460 | *SlAlba1* | 5 | -1 | 1 | 21 | 643 | 663 | UGAUAUGUGGCAUUCAAUCGA | AUAUGAGGAUGCUACAUAUCA | Cleavage | Nucleic acid binding protein |
| rgl-miR7797b | *SlAlba1* | 4 | -1 | 1 | 23 | 394 | 416 | UUUGAUUUCGUCUUACAUUUUUC | UUGGAAUGAAAGAUGAGGCCAAA | Cleavage | Nucleic acid binding protein |
| rgl-miR7972 | *SlAlba1* | 5 | -1 | 1 | 21 | 458 | 478 | UUGUCAGGCUUGUUAUUCUCC | GAAGAGCGAGAAGUUUGAUAA | Cleavage | Nucleic acid binding protein |
| sly-miR9477-3p | *SlAlba1* | 4.5 | -1 | 1 | 24 | 846 | 869 | UUGGGAAAGGGAACAACUGAUAGU | ACAUUUUGCAGUUCCCUUUUUCAA | Cleavage | Nucleic acid binding protein |
| smo-miR1081 | *SlAlba1* | 4.5 | -1 | 1 | 21 | 750 | 770 | UGAGGCUUGCCUUUGAUUCUC | UGACAGCAAAGGCAACCCUCA | Cleavage | Nucleic acid binding protein |
| ssl-miR948 | *SlAlba1* | 5 | -1 | 1 | 20 | 117 | 137 | UGUGGCU-GUGUGGGUUCCGG | GCUGCACCCACACUAGCCACA | Cleavage | Nucleic acid binding protein |
| tae-miR9668-5p | *SlAlba1* | 5 | -1 | 1 | 21 | 589 | 609 | CCAAUGACAAGUAUUUUCGGA | AAGGAUGCUACUUGUUAUUGU | Cleavage | Nucleic acid binding protein |
| vvi-miR3624-5p | *SlAlba1* | 4.5 | -1 | 1 | 21 | 265 | 285 | UAGUAUGCUGCUGUCUUUAGA | GGUACAUGCAGCAGCAUAAUG | Cleavage | Nucleic acid binding protein |
| zma-miR171h-5p | *SlAlba1* | 4.5 | -1 | 1 | 21 | 481 | 501 | UGGUAUUGUUUCGGCUCAUGU | UGAUGACACCAAGCAAUACUA | Cleavage | Nucleic acid binding protein |
| aly-miR774a-5p.2 | *SlAlba2* | 5 | -1 | 1 | 21 | 680 | 700 | AGAUAUGGGUGACUAAUACUU | GAGCAUCAGUUGCUUAUGUUU | Cleavage | Ribonuclease P protein subunit p25 |
| aly-miR823-5p | *SlAlba2* | 5 | -1 | 1 | 20 | 812 | 831 | CUUGUAUGAUCACUAACCAU | AUGACUUGUGAUCGUGCAAA | Cleavage | Ribonuclease P protein subunit p25 |
| aly-miR833-5p | *SlAlba2* | 5 | -1 | 1 | 23 | 50 | 72 | UGUUAGUCUGAACUCGGUCUAGU | AGGAGAAUGAGAUUAGAAUAACA | Cleavage | Ribonuclease P protein subunit p25 |
| aly-miR838-5p | *SlAlba2* | 5 | -1 | 1 | 21 | 714 | 734 | UGCAAGAAUGAGAAGCAAAGC | AAUUCACUUGUUGUUUUUGCA | Cleavage | Ribonuclease P protein subunit p25 |
| aly-miR847-5p | *SlAlba2* | 4 | -1 | 1 | 21 | 108 | 128 | UCUUGAUGAAGAGGAAUGGAA | UACUAAUCUUCUUCUUCAGGA | Cleavage | Ribonuclease P protein subunit p25 |
| aqc-miR408 | *SlAlba2* | 5 | -1 | 1 | 21 | 625 | 645 | UGCUCUGCCUCAUCCUUGUCU | GUACAGGGACGAGGUAGAGGU | Cleavage | Ribonuclease P protein subunit p25 |
| aqc-miR477d | *SlAlba2* | 5 | -1 | 1 | 20 | 269 | 288 | CUCUUCUUCAAAGGCUUCUA | GGGAACCUUUAGAAGAAGGA | Translation | Ribonuclease P protein subunit p25 |
| bdi-miR7719-5p | *SlAlba2* | 5 | -1 | 1 | 21 | 544 | 564 | UCCGCCAUACAUGUUUCUAUC | CGUGGUAACAACUAUGGUGGA | Translation | Ribonuclease P protein subunit p25 |
| bdi-miR7753-5p | *SlAlba2* | 5 | -1 | 1 | 21 | 418 | 438 | AUGUCUUCUUCCUUGCUCAUC | UAUGAUUAUGGAGGAGGGCGU | Cleavage | Ribonuclease P protein subunit p25 |
| bdi-miR7784a-3p | *SlAlba2* | 5 | -1 | 1 | 24 | 140 | 162 | CAUAGACUUAUGAGACGAGUACAU | AAGAAAUUG-CUCUUAAGUCUAUG | Translation | Ribonuclease P protein subunit p25 |
| bdi-miR7784b-3p | *SlAlba2* | 5 | -1 | 1 | 24 | 140 | 162 | CAUAGACUUAUGAGACGAGUACAU | AAGAAAUUG-CUCUUAAGUCUAUG | Translation | Ribonuclease P protein subunit p25 |
| bdi-miR9492 | *SlAlba2* | 5 | -1 | 1 | 21 | 551 | 571 | UAUCUACUCUGUCAUGGUAUC | ACAACUAUGGUGGAGUAGCUG | Cleavage | Ribonuclease P protein subunit p25 |
| bra-miR172d-5p | *SlAlba2* | 5 | -1 | 1 | 21 | 432 | 452 | GCAGCAUCAUUAAGAUUCACA | AGGGCGUUUUAAUGAUGGUGU | Cleavage | Ribonuclease P protein subunit p25 |
| cpa-miR8146 | *SlAlba2* | 5 | -1 | 1 | 24 | 100 | 123 | AGGAAGACGGUGAGUAGAAGCCAA | ACUUAUGCUACUAAUCUUCUUCUU | Cleavage | Ribonuclease P protein subunit p25 |
| cpa-miR8148 | *SlAlba2* | 4.5 | -1 | 1 | 24 | 380 | 403 | UGACUGGGUCUGCUGACGUGGCAU | CACCUAUUCCAGCAGAUCAAGUCA | Cleavage | Ribonuclease P protein subunit p25 |
| gma-miR5780d | *SlAlba2* | 5 | -1 | 1 | 22 | 760 | 781 | UGUUUUGAGUUUCUGAUAAAUU | AAUCUAACAGAAACUCAAGCCC | Cleavage | Ribonuclease P protein subunit p25 |
| gra-miR8659a | *SlAlba2* | 5 | -1 | 1 | 24 | 124 | 147 | AAUUUUUUAAGGUUGUUUGUGGAA | CAGGAGAAAGGAUCUAAAGAAAUU | Cleavage | Ribonuclease P protein subunit p25 |
| gra-miR8659b | *SlAlba2* | 5 | -1 | 1 | 24 | 124 | 147 | AAUUUUUUAAGGUUGUUUGUGGAA | CAGGAGAAAGGAUCUAAAGAAAUU | Cleavage | Ribonuclease P protein subunit p25 |
| hbr-miR6171 | *SlAlba2* | 4.5 | -1 | 1 | 21 | 218 | 238 | AUGUGGAUUGCUGAAGGCUUU | UUGGCCUUCAUCAGAUCACAU | Translation | Ribonuclease P protein subunit p25 |
| hme-miR-2765 | *SlAlba2* | 5 | -1 | 1 | 22 | 550 | 571 | UGGUAACUCCACCACCGUUGGC | AACAACUAUGGUGGAGUAGCUG | Cleavage | Ribonuclease P protein subunit p25 |
| hvu-miR6202 | *SlAlba2* | 5 | -1 | 1 | 20 | 739 | 758 | UGAAGAUUUUAAGCAUUGAA | GUCCAUGCUUGUGUUCUUCA | Cleavage | Ribonuclease P protein subunit p25 |
| mtr-miR2089-3p | *SlAlba2* | 5 | -1 | 1 | 22 | 96 | 117 | AGGAUUGGUGUAAUAGGUAAAA | CAUUACUUAUGCUACUAAUCUU | Translation | Ribonuclease P protein subunit p25 |
| mtr-miR2585a | *SlAlba2* | 5 | -1 | 1 | 22 | 25 | 46 | CAGGAUUAGCGAUUACAGGGAC | AAGCCAAUAAUUGAUAAUCCUA | Cleavage | Ribonuclease P protein subunit p25 |
| mtr-miR2585b | *SlAlba2* | 5 | -1 | 1 | 22 | 25 | 46 | CAGGAUUAGCGAUUACAGGGAC | AAGCCAAUAAUUGAUAAUCCUA | Cleavage | Ribonuclease P protein subunit p25 |
| mtr-miR2636 | *SlAlba2* | 5 | -1 | 1 | 21 | 397 | 417 | UUUGGUUAGUGUGCUGAAUAU | CAAGUCAGACCACUGACCGAA | Cleavage | Ribonuclease P protein subunit p25 |
| mtr-miR5217 | *SlAlba2* | 5 | -1 | 1 | 22 | 825 | 846 | AGGUCAUUUUGAACGGUCGGAU | GUGCAAAAGAUCAAAAUGAUCU | Cleavage | Ribonuclease P protein subunit p25 |
| mtr-miR5271a | *SlAlba2* | 5 | -1 | 1 | 24 | 315 | 338 | CGGAUAAUUGUGGUUACUAACGGU | UGUGUCAGUCAUAACAAUUAUCCU | Cleavage | Ribonuclease P protein subunit p25 |
| mtr-miR5271b | *SlAlba2* | 5 | -1 | 1 | 24 | 315 | 338 | CGGAUAAUUGUGGUUACUAACGGU | UGUGUCAGUCAUAACAAUUAUCCU | Cleavage | Ribonuclease P protein subunit p25 |
| mtr-miR5752a | *SlAlba2* | 3.5 | -1 | 1 | 21 | 653 | 673 | CAUUGUUUGGUUUAGUACAAA | AGGGUACUAAAUCAAAUGAUA | Cleavage | Ribonuclease P protein subunit p25 |
| mtr-miR5752b | *SlAlba2* | 3.5 | -1 | 1 | 21 | 653 | 673 | CAUUGUUUGGUUUAGUACAAA | AGGGUACUAAAUCAAAUGAUA | Cleavage | Ribonuclease P protein subunit p25 |
| osa-miR166k-5p | *SlAlba2* | 5 | -1 | 1 | 21 | 394 | 414 | GGUUUGUUGUCUGGCUCGAGG | GAUCAAGUCAGACCACUGACC | Cleavage | Ribonuclease P protein subunit p25 |
| osa-miR1853-5p | *SlAlba2* | 3.5 | -1 | 1 | 24 | 684 | 707 | AGCAUUCAAACAUUCCCAAUUACC | AUCAGUUGCUUAUGUUUGAAUGUU | Cleavage | Ribonuclease P protein subunit p25 |
| osa-miR2055 | *SlAlba2* | 5 | -1 | 1 | 21 | 110 | 130 | UUUCCUUGGGAAGGUGGUUUC | CUAAUCUUCUUCUUCAGGAGA | Cleavage | Ribonuclease P protein subunit p25 |
| osa-miR419 | *SlAlba2* | 3.5 | -1 | 1 | 21 | 210 | 230 | UGAUGAAUGCUGACGAUGUUG | GAAGAUCGUUGGCCUUCAUCA | Cleavage | Ribonuclease P protein subunit p25 |
| ppt-miR1039-5p | *SlAlba2* | 3.5 | -1 | 1 | 21 | 124 | 143 | UCUUUGGGUCUUUCUCUCCUG | CAGGAGA-AAGGAUCUAAAGA | Cleavage | Ribonuclease P protein subunit p25 |
| ppt-miR1048-5p | *SlAlba2* | 4.5 | -1 | 1 | 21 | 736 | 756 | UAGAACAUGAGUGUAGACGAC | UGUGUCCAUGCUUGUGUUCUU | Cleavage | Ribonuclease P protein subunit p25 |
| ppt-miR419 | *SlAlba2* | 5 | -1 | 1 | 21 | 210 | 230 | UGAUGAAUGAUGACGAUGUAU | GAAGAUCGUUGGCCUUCAUCA | Translation | Ribonuclease P protein subunit p25 |
| ptc-miR6466-5p | *SlAlba2* | 5 | -1 | 1 | 21 | 682 | 702 | UCUGGUAUGAGCAUUUGAUGA | GCAUCAGUUGCUUAUGUUUGA | Cleavage | Ribonuclease P protein subunit p25 |
| ptc-miR7814 | *SlAlba2* | 4.5 | -1 | 1 | 21 | 827 | 847 | UAGAUUGUUUUUAUGCUUUGA | GCAAAAGAUCAAAAUGAUCUA | Cleavage | Ribonuclease P protein subunit p25 |
| pti-miR5483 | *SlAlba2* | 5 | -1 | 1 | 23 | 124 | 146 | AUUUUUUUGGAUCGUGUGAUAUA | CAGGAGAAAGGAUCUAAAGAAAU | Cleavage | Ribonuclease P protein subunit p25 |
| sly-miR5303 | *SlAlba2* | 4 | -1 | 1 | 21 | 742 | 762 | UUUUUGAAGAGUUCGAGCAAC | CAUGCUUGUGUUCUUCAAAAU | Cleavage | Ribonuclease P protein subunit p25 |
| smo-miR1088-3p | *SlAlba2* | 4.5 | -1 | 1 | 21 | 472 | 492 | GCGUGCUCUUUUUCUUCUGUC | GGCGGAUGGAAAGGAGGACGU | Cleavage | Ribonuclease P protein subunit p25 |
| vvi-miR3633b-5p | *SlAlba2* | 2.5 | -1 | 1 | 21 | 369 | 389 | GGAAUGGGUGGCUGGGAUCUA | CGGGUAUCAGCCACCUAUUCC | Cleavage | Ribonuclease P protein subunit p25 |
| zma-miR164e-3p | *SlAlba2* | 5 | -1 | 1 | 21 | 463 | 483 | CAUGUGUCCGCCCUCUCCACC | GGUGGAGAGGGCGGAUGGAAA | Cleavage | Ribonuclease P protein subunit p25 |
| ahy-miR3513-5p | *SlAlba3* | 5 | -1 | 1 | 21 | 871 | 891 | UUAAUUUCUGAGUUUGUCAUC | CUUGAAGAAUACAGAGAUUAU | Translation | Ribonuclease P protein subunit p25 |
| aly-miR156h-5p | *SlAlba3* | 5 | -1 | 1 | 20 | 1017 | 1036 | UGACAGAAGAAAGAGAGCAC | AUGUUUUCUUUUCUUUGUUU | Cleavage | Ribonuclease P protein subunit p25 |
| aly-miR399f-5p | *SlAlba3* | 4.5 | -1 | 1 | 22 | 1140 | 1161 | GGGCAAGAUCACCAUUGGCAGA | AUGUUCAGUGUUGAUCUUGUUC | Cleavage | Ribonuclease P protein subunit p25 |
| aly-miR858-3p | *SlAlba3* | 4.5 | -1 | 1 | 21 | 1052 | 1072 | GGUCGAGCAGACAACGAAGAU | AUCUUUAUUCUCUGCUUGAUU | Cleavage | Ribonuclease P protein subunit p25 |
| aqc-miR477d | *SlAlba3* | 4.5 | -1 | 1 | 20 | 362 | 381 | CUCUUCUUCAAAGGCUUCUA | GGGAACCCCUUGAAGAAGGU | Cleavage | Ribonuclease P protein subunit p25 |
| aqu-miR-2021-3p | *SlAlba3* | 5 | -1 | 1 | 21 | 457 | 477 | UGGUGGUCGGUGUUUCGUGGA | GCUGUGGGGUACCAACCACCA | Cleavage | Ribonuclease P protein subunit p25 |
| ath-miR156h | *SlAlba3* | 5 | -1 | 1 | 20 | 1017 | 1036 | UGACAGAAGAAAGAGAGCAC | AUGUUUUCUUUUCUUUGUUU | Cleavage | Ribonuclease P protein subunit p25 |
| ath-miR413 | *SlAlba3* | 5 | -1 | 1 | 21 | 482 | 502 | AUAGUUUCUCUUGUUCUGCAC | CAGCAGACCAGGUGAAAGUGU | Cleavage | Ribonuclease P protein subunit p25 |
| ath-miR414 | *SlAlba3* | 3.5 | -1 | 1 | 21 | 618 | 638 | UCAUCUUCAUCAUCAUCGUCA | GUAUGAUGAUGGUGGGUAUGA | Cleavage | Ribonuclease P protein subunit p25 |
| ath-miR4228-5p | *SlAlba3* | 4.5 | -1 | 1 | 21 | 234 | 254 | AUAGCCUUGAACGCCGUCGUU | GGAGAUCGUGUUCAAGGCAAU | Cleavage | Ribonuclease P protein subunit p25 |
| ath-miR8184 | *SlAlba3* | 4.5 | -1 | 1 | 21 | 961 | 981 | UUUGGUCUGAUUACGAAUGUA | UCUAUCUGUAAUCAGAGUAGA | Cleavage | Ribonuclease P protein subunit p25 |
| ath-miR854a | *SlAlba3* | 4.5 | -1 | 1 | 21 | 71 | 91 | GAUGAGGAUAGGGAGGAGGAG | AAUCUUAUCCUUAUUCUUAUA | Cleavage | Ribonuclease P protein subunit p25 |
| ath-miR854b | *SlAlba3* | 4.5 | -1 | 1 | 21 | 71 | 91 | GAUGAGGAUAGGGAGGAGGAG | AAUCUUAUCCUUAUUCUUAUA | Cleavage | Ribonuclease P protein subunit p25 |
| ath-miR867 | *SlAlba3* | 5 | -1 | 1 | 21 | 228 | 248 | UUGAACAUGGUUUAUUAGGAA | AUCAGAGGAGAUCGUGUUCAA | Cleavage | Ribonuclease P protein subunit p25 |
| bdi-miR5198 | *SlAlba3* | 5 | -1 | 1 | 21 | 1044 | 1064 | GGGGAAAAGAGAUUGAGGGAG | UAUCUUCUAUCUUUAUUCUCU | Cleavage | Ribonuclease P protein subunit p25 |
| bdi-miR7741-5p.1 | *SlAlba3* | 4.5 | -1 | 1 | 21 | 741 | 761 | UUUUAAUUGUGGAAGCUCUUG | UGGUGGCUUCUACAAUCAAGA | Cleavage | Ribonuclease P protein subunit p25 |
| cca-miR6108f | *SlAlba3* | 5 | -1 | 1 | 21 | 71 | 91 | UAUGGUGAGAAGGGUAAGAAG | AAUCUUAUCCUUAUUCUUAUA | Cleavage | Ribonuclease P protein subunit p25 |
| cre-miR1158 | *SlAlba3* | 5 | -1 | 1 | 20 | 307 | 326 | ACUUGGAGGAGGCCACUGGC | AUUGUUGGUCUUCACCAAGU | Cleavage | Ribonuclease P protein subunit p25 |
| cre-miR1169-3p | *SlAlba3* | 4.5 | -1 | 1 | 21 | 316 | 336 | UGUGGAUGUUGCUUGCUGGAU | CUUCACCAAGUAACGUCUAUU | Cleavage | Ribonuclease P protein subunit p25 |
| csi-miR3951 | *SlAlba3* | 5 | -1 | 1 | 21 | 1114 | 1134 | UAGAUAAAGAUGAGAGAAAAA | UCUUUGAUUUGUCUUUGUUUG | Cleavage | Ribonuclease P protein subunit p25 |
| ghr-miR7492a | *SlAlba3* | 5 | -1 | 1 | 23 | 878 | 900 | CUAUAGAACAUGAUCUUUAGCGG | AAUACAGAGAUUAUGUGCUAUGA | Cleavage | Ribonuclease P protein subunit p25 |
| ghr-miR7492b | *SlAlba3* | 5 | -1 | 1 | 23 | 878 | 900 | CUAUAGAACAUGAUCUUUAGCGG | AAUACAGAGAUUAUGUGCUAUGA | Cleavage | Ribonuclease P protein subunit p25 |
| ghr-miR7493 | *SlAlba3* | 5 | -1 | 1 | 24 | 1121 | 1144 | AAUAUUUUAAUAAUUCAAUCGUCA | UUUGUCUUUGUUUGUUGAAAUGUU | Cleavage | Ribonuclease P protein subunit p25 |
| gma-miR4399 | *SlAlba3* | 5 | -1 | 1 | 22 | 1117 | 1138 | UUAACGAAAAAGGACUAACGAC | UUGAUUUGUCUUUGUUUGUUGA | Cleavage | Ribonuclease P protein subunit p25 |
| gma-miR4415a-5p | *SlAlba3* | 5 | -1 | 1 | 21 | 1029 | 1049 | AAGUUGUGAUGAGAAUCAAUG | CUUUGUUUUUCAUGAUAUCUU | Cleavage | Ribonuclease P protein subunit p25 |
| gma-miR9722 | *SlAlba3* | 4.5 | -1 | 1 | 21 | 1016 | 1036 | UAAUAGAGGGAAGAAGAUGAA | GAUGUUUUCUUUUCUUUGUUU | Cleavage | Ribonuclease P protein subunit p25 |
| gma-miR9730 | *SlAlba3* | 4 | -1 | 1 | 22 | 626 | 647 | CGAUUGCUGUCAUAACUGCUGC | AUGGUGGGUAUGAUCGCAAUCG | Cleavage | Ribonuclease P protein subunit p25 |
| gma-miR9736 | *SlAlba3* | 4 | -1 | 1 | 21 | 1020 | 1040 | UGAAAGACAAACAAAGGUGGG | UUUUCUUUUCUUUGUUUUUCA | Cleavage | Ribonuclease P protein subunit p25 |
| gra-miR7504n | *SlAlba3* | 5 | -1 | 1 | 24 | 58 | 81 | AGGAUAAAAUUACUGAUGUGGCAU | GAAGAAUAAUACUAAUCUUAUCCU | Cleavage | Ribonuclease P protein subunit p25 |
| gra-miR7506b | *SlAlba3* | 5 | -1 | 1 | 20 | 901 | 920 | CUGGGACAUGGCGUUGGCAA | GUGCCUGCUCCAUGUUCUAU | Cleavage | Ribonuclease P protein subunit p25 |
| gra-miR8712 | *SlAlba3* | 5 | -1 | 1 | 24 | 183 | 206 | AUAUCAUUGGUGAUGUAUCGUCUU | GCGAAGCUAUAUCACUUAUGCUAU | Cleavage | Ribonuclease P protein subunit p25 |
| gra-miR8750 | *SlAlba3* | 5 | -1 | 1 | 24 | 596 | 619 | UCUUAGUUGGCAUAUACUCAAGGA | GAAAUGGAUUUAUGCCGGCUGAGU | Cleavage | Ribonuclease P protein subunit p25 |
| hme-miR-307 | *SlAlba3* | 5 | -1 | 1 | 22 | 835 | 856 | CACAACCUCCUUGAGUGAGCGA | GGGCCGAUCCAAGGAGGUGGUG | Cleavage | Ribonuclease P protein subunit p25 |
| hvu-miR6190 | *SlAlba3* | 4.5 | -1 | 1 | 21 | 1013 | 1033 | CGAGGAAAGGAAGAAGCCAUG | GGUGAUGUUUUCUUUUCUUUG | Cleavage | Ribonuclease P protein subunit p25 |
| mdm-miR396a | *SlAlba3* | 5 | -1 | 1 | 21 | 445 | 465 | UUCCACAGCUUUCUUGAACAG | CUGGAUAAGAAUGCUGUGGGG | Translation | Ribonuclease P protein subunit p25 |
| mtr-miR2592a-3p | *SlAlba3* | 5 | -1 | 1 | 21 | 906 | 927 | GAAAAACAU-GAAUGUCGAGCG | UGCUCCAUGUUCUAUGUUUUUC | Cleavage | Ribonuclease P protein subunit p25 |
| mtr-miR5239 | *SlAlba3* | 5 | -1 | 1 | 21 | 1044 | 1064 | UGGGAGAAAAGAUAGAAUGUG | UAUCUUCUAUCUUUAUUCUCU | Cleavage | Ribonuclease P protein subunit p25 |
| mtr-miR5561-5p | *SlAlba3* | 5 | -1 | 1 | 21 | 1122 | 1142 | CAUUUGGAGAGACAUAGACAA | UUGUCUUUGUUUGUUGAAAUG | Cleavage | Ribonuclease P protein subunit p25 |
| osa-miR166g-5p | *SlAlba3* | 5 | -1 | 1 | 21 | 1112 | 1132 | AAUGGAGGCUGAUCCAAGAUC | AUUCUUUGAUUUGUCUUUGUU | Translation | Ribonuclease P protein subunit p25 |
| osa-miR1868 | *SlAlba3* | 5 | -1 | 1 | 24 | 1020 | 1043 | UCACGGAAAACGAGGGAGCAGCCA | UUUUCUUUUCUUUGUUUUUCAUGA | Cleavage | Ribonuclease P protein subunit p25 |
| osa-miR413 | *SlAlba3* | 4.5 | -1 | 1 | 21 | 482 | 502 | CUAGUUUCACUUGUUCUGCAC | CAGCAGACCAGGUGAAAGUGU | Cleavage | Ribonuclease P protein subunit p25 |
| osa-miR414 | *SlAlba3* | 3 | -1 | 1 | 21 | 618 | 638 | UCAUCCUCAUCAUCAUCGUCC | GUAUGAUGAUGGUGGGUAUGA | Cleavage | Ribonuclease P protein subunit p25 |
| osa-miR5339 | *SlAlba3* | 5 | -1 | 1 | 21 | 948 | 968 | CAGAUAGAGAAUCUUCUCAGA | UAACAGUAGGUUAUCUAUCUG | Cleavage | Ribonuclease P protein subunit p25 |
| osa-miR5500 | *SlAlba3* | 5 | -1 | 1 | 22 | 1084 | 1105 | AUCACUGAUGAAAUCUUGCGGC | CAAGUGUAAUUUCGUUAGUGAG | Cleavage | Ribonuclease P protein subunit p25 |
| osa-miR5508 | *SlAlba3* | 5 | -1 | 1 | 21 | 948 | 968 | UAGAUGGCUGAUCUGGUGUGG | UAACAGUAGGUUAUCUAUCUG | Cleavage | Ribonuclease P protein subunit p25 |
| osa-miR5544 | *SlAlba3* | 5 | -1 | 1 | 22 | 897 | 918 | AGAACACGGAGUAGAAGUUGGU | AUGAGUGCCUGCUCCAUGUUCU | Cleavage | Ribonuclease P protein subunit p25 |
| ppt-miR1039-5p | *SlAlba3* | 4 | -1 | 1 | 21 | 567 | 587 | UCUUUGGGUCUUUCUCUCCUG | AGGAAGGGGAAGGUCCAGAGG | Cleavage | Ribonuclease P protein subunit p25 |
| ppt-miR1046-3p | *SlAlba3* | 5 | -1 | 1 | 21 | 911 | 931 | UGGUGAAAAAUAUGAAAAAUC | CAUGUUCUAUGUUUUUCAUUC | Cleavage | Ribonuclease P protein subunit p25 |
| ppt-miR1222d | *SlAlba3* | 4 | -1 | 1 | 21 | 355 | 375 | UUUAAGGGGUUCACUGGUAUA | GAUACAUGGGAACCCCUUGAA | Cleavage | Ribonuclease P protein subunit p25 |
| ppt-miR414 | *SlAlba3* | 5 | -1 | 1 | 21 | 612 | 632 | UCAUCCUCAUCAUCCUCGUCC | GGCUGAGUAUGAUGAUGGUGG | Cleavage | Ribonuclease P protein subunit p25 |
| ptc-miR6475 | *SlAlba3* | 5 | -1 | 1 | 21 | 1023 | 1043 | UCUUGAGAAGUAAAGAACGAC | UCUUUUCUUUGUUUUUCAUGA | Cleavage | Ribonuclease P protein subunit p25 |
| ptc-miR7840 | *SlAlba3* | 5 | -1 | 1 | 21 | 926 | 946 | CAAGGAGUAAUUAGUGACAUC | UCAUUCGUUAGUUACUCCGUG | Cleavage | Ribonuclease P protein subunit p25 |
| rgl-miR7807b-3p | *SlAlba3* | 5 | -1 | 1 | 22 | 484 | 505 | UUGAGAUUUUCAUAUAGUUACU | GCAGACCAGGUGAAAGUGUCAA | Cleavage | Ribonuclease P protein subunit p25 |
| sbi-miR5566 | *SlAlba3* | 5 | -1 | 1 | 21 | 840 | 860 | UCAGCAUCACCUCCCUGUUGU | GAUCCAAGGAGGUGGUGCUUA | Cleavage | Ribonuclease P protein subunit p25 |
| vvi-miR156e | *SlAlba3* | 5 | -1 | 1 | 20 | 1017 | 1036 | UGACAGAGGAGAGUGAGCAC | AUGUUUUCUUUUCUUUGUUU | Cleavage | Ribonuclease P protein subunit p25 |
| zma-miR169m-3p | *SlAlba3* | 5 | -1 | 1 | 20 | 166 | 185 | GGCAUCCAUUCUUGGCUAAG | ACUAGUCAGGGAAGGAUGCG | Cleavage | Ribonuclease P protein subunit p25 |
| zma-miR2275d-5p | *SlAlba3* | 4.5 | -1 | 1 | 21 | 1018 | 1038 | AGAGUUGGAGGAAAGAAAACU | UGUUUUCUUUUCUUUGUUUUU | Cleavage | Ribonuclease P protein subunit p25 |
| zma-miR408b-5p | *SlAlba3* | 5 | -1 | 1 | 21 | 1110 | 1130 | CAGGGACGAGGCAGAGCAUGG | ACAUUCUUUGAUUUGUCUUUG | Translation | Ribonuclease P protein subunit p25 |
| ata-miR171a-5p | *SlAlba4* | 5 | -1 | 1 | 20 | 388 | 407 | UGGUAUUGUUUCGGCUCAUG | CAGGAGCCCGACCGAUACCA | Cleavage | Nucleic acid binding protein |
| ata-miR6201-5p | *SlAlba4* | 4.5 | -1 | 1 | 22 | 149 | 170 | UGACCCUGAGGCACUCAUACCG | CAAGAUGAGUGUCUCAGAGACA | Cleavage | Nucleic acid binding protein |
| ata-miR9863a-5p | *SlAlba4* | 3.5 | -1 | 1 | 21 | 342 | 362 | UGUUAUGAUCUGCUUCUCAUC | GUUGAGAAGAAGAUCAGGACA | Cleavage | Nucleic acid binding protein |
| ata-miR9863b-5p | *SlAlba4* | 3.5 | -1 | 1 | 21 | 342 | 362 | UGUUAUGAUCUGCUUCUCAUC | GUUGAGAAGAAGAUCAGGACA | Cleavage | Nucleic acid binding protein |
| ath-miR167c-3p | *SlAlba4* | 5 | -1 | 1 | 23 | 621 | 643 | UAGGUCAUGCUGGUAGUUUCACC | UAAAAAAAAAGCAGCAUGGUUUA | Cleavage | Nucleic acid binding protein |
| ath-miR5017-3p | *SlAlba4* | 5 | -1 | 1 | 21 | 18 | 38 | UUAUACCAAAUUAAUAGCAAA | UCUGUUACUGUUUUAGUAUAA | Translation | Nucleic acid binding protein |
| ath-miR5020b | *SlAlba4* | 4.5 | -1 | 1 | 21 | 208 | 229 | AUGGCAUGAAAGAAG-GUGAGA | AAUCACUCUUCUUUUAUGUUAA | Cleavage | Nucleic acid binding protein |
| ath-miR5020c | *SlAlba4* | 5 | -1 | 1 | 21 | 207 | 228 | UGGCAUGGAAGAAG-GUGAGAC | AAAUCACUCUUCUUUUAUGUUA | Cleavage | Nucleic acid binding protein |
| ath-miR5648-5p | *SlAlba4* | 5 | -1 | 1 | 22 | 499 | 520 | UUUGGAAAUAUUUGGCUUGACU | AGCAGAACUGAUUAUUUCCAAA | Translation | Nucleic acid binding protein |
| bdi-miR5185l-3p | *SlAlba4* | 4 | -1 | 1 | 21 | 217 | 237 | UUUGGAGAUUGACUUAGAAGC | UCUUUUAUGUUAAUCUCUCAA | Cleavage | Nucleic acid binding protein |
| bra-miR9559-5p | *SlAlba4* | 5 | -1 | 1 | 21 | 172 | 192 | UUUGGAUUUUGGUCAUUGUUG | AGAAGAAGAACAGAAUCCAAG | Cleavage | Nucleic acid binding protein |
| csi-miR479 | *SlAlba4* | 5 | -1 | 1 | 22 | 388 | 409 | UGUGAUAUUGGUUCGGCUCAUC | CAGGAGCCCGACCGAUACCAAA | Cleavage | Nucleic acid binding protein |
| ddi-miR-7100 | *SlAlba4* | 3.5 | -1 | 1 | 21 | 18 | 38 | UUAUAAUGAAACGGUAAUGAU | UCUGUUACUGUUUUAGUAUAA | Cleavage | Nucleic acid binding protein |
| gma-miR1534 | *SlAlba4* | 5 | -1 | 1 | 20 | 91 | 110 | UAUUUUGGGUAAAUAGUCAU | AUUAGGGUUUACCUGAAGUA | Cleavage | Nucleic acid binding protein |
| gma-miR4344 | *SlAlba4* | 5 | -1 | 1 | 24 | 271 | 294 | AAGUAGACAUUCUAAGACGUUGCU | UCUCUGCUCUUGGGAUGGCUAUUU | Cleavage | Nucleic acid binding protein |
| gma-miR5773 | *SlAlba4* | 3.5 | -1 | 1 | 22 | 590 | 611 | UUUUUAAAAGGUUCAGUUAGGU | CUUUAAUUUGAUCUUUUGGAAA | Cleavage | Nucleic acid binding protein |
| gma-miR9742 | *SlAlba4* | 5 | -1 | 1 | 21 | 618 | 638 | UGUGUUGUUUGUUUUGUAGCA | UUUUAAAAAAAAAGCAGCAUG | Translation | Nucleic acid binding protein |
| gma-miR9752 | *SlAlba4* | 4.5 | -1 | 1 | 21 | 479 | 499 | UGCUUCUUCUUUUCCCUGUUU | AAACGGGGACAAUGAGGAGCA | Cleavage | Nucleic acid binding protein |
| gra-miR7505b | *SlAlba4* | 5 | -1 | 1 | 21 | 505 | 525 | ACAGCUUUAGAAAUCAUCCCU | ACUGAUUAUUUCCAAAUCUGU | Cleavage | Nucleic acid binding protein |
| hvu-miR397a | *SlAlba4* | 5 | -1 | 1 | 21 | 553 | 573 | CCGUUGAGUGCAGCGUUGAUG | AAUCUAUACUGCAUUCAGCUG | Cleavage | Nucleic acid binding protein |
| hvu-miR6201 | *SlAlba4* | 4.5 | -1 | 1 | 22 | 149 | 170 | UGACCCUGAGGCACUCAUACCG | CAAGAUGAGUGUCUCAGAGACA | Cleavage | Nucleic acid binding protein |
| mdm-miR7127a | *SlAlba4* | 4.5 | -1 | 1 | 21 | 438 | 458 | AUACUCAUCGAAUUUGUCAUA | ACUGAGAAGUUUGAUGAGUUA | Cleavage | Nucleic acid binding protein |
| mdm-miR7127b | *SlAlba4* | 4.5 | -1 | 1 | 21 | 438 | 458 | AUACUCAUCGAAUUUGUCAUA | ACUGAGAAGUUUGAUGAGUUA | Cleavage | Nucleic acid binding protein |
| mtr-miR2609a | *SlAlba4* | 4.5 | -1 | 1 | 21 | 498 | 518 | UGGAAGUAAUAGGUUCUCACU | CAGCAGAACUGAUUAUUUCCA | Translation | Nucleic acid binding protein |
| mtr-miR2609b | *SlAlba4* | 4.5 | -1 | 1 | 21 | 498 | 518 | UGGAAGUAAUAGGUUCUCACU | CAGCAGAACUGAUUAUUUCCA | Translation | Nucleic acid binding protein |
| mtr-miR5222 | *SlAlba4* | 5 | -1 | 1 | 22 | 670 | 691 | UUACAGGAGAAGAAUGUAUGGC | UGUAUGCCUUUUUUUUUUUUAA | Cleavage | Nucleic acid binding protein |
| mtr-miR5237 | *SlAlba4* | 4.5 | -1 | 1 | 21 | 589 | 609 | UUCAAAAGAUUUAGUUGGGAU | CCUUUAAUUUGAUCUUUUGGA | Cleavage | Nucleic acid binding protein |
| mtr-miR5238 | *SlAlba4* | 5 | -1 | 1 | 21 | 673 | 693 | UGUAGAAAAAACAAAGGGCAA | AUGCCUUUUUUUUUUUUAACC | Cleavage | Nucleic acid binding protein |
| mtr-miR5248 | *SlAlba4* | 5 | -1 | 1 | 21 | 764 | 784 | UUUUUAGUUGGCAUGCAUUCA | UUUAUGCCUGGUAGUUAAAAA | Translation | Nucleic acid binding protein |
| mtr-miR5290 | *SlAlba4* | 4.5 | -1 | 1 | 24 | 28 | 51 | AAUUUGGAGAGAGAUAGACACAUA | UUUUAGUAUAAUUUUCCCCAAAUU | Cleavage | Nucleic acid binding protein |
| nta-miR6145d | *SlAlba4* | 5 | -1 | 1 | 21 | 235 | 255 | AUUGUUACAUGUAACACUGGC | CAAAGAGGUACAUGCAACAGU | Cleavage | Nucleic acid binding protein |
| nta-miR6145e | *SlAlba4* | 5 | -1 | 1 | 21 | 235 | 255 | AUUGUUACAUGUAGCACUGGC | CAAAGAGGUACAUGCAACAGU | Cleavage | Nucleic acid binding protein |
| osa-miR5486 | *SlAlba4* | 5 | -1 | 1 | 21 | 660 | 680 | AGGGGCUUGCAUAUUCUACCA | UUGUUGGAUGUGUAUGCCUUU | Cleavage | Nucleic acid binding protein |
| osa-miR5492 | *SlAlba4* | 3.5 | -1 | 1 | 21 | 514 | 534 | AGAAGGAGAAUAGAUAUGGUU | UUCCAAAUCUGUUCUCUUCCU | Cleavage | Nucleic acid binding protein |
| ptc-miR169u-3p | *SlAlba4* | 4.5 | -1 | 1 | 21 | 68 | 88 | GGCAGUCUCCUUUGGCUAUCC | CAAAAGGGAAAGGAGGCUGCG | Cleavage | Nucleic acid binding protein |
| ptc-miR171l-5p | *SlAlba4* | 4.5 | -1 | 1 | 22 | 388 | 409 | UGUGAUAUUGGUCCGGCUCAUC | CAGGAGCCCGACCGAUACCAAA | Cleavage | Nucleic acid binding protein |
| ptc-miR475a-5p | *SlAlba4* | 3.5 | -1 | 1 | 21 | 273 | 293 | AAUGGCCAUUGUAAGAGUAGA | UCUGCUCUUGGGAUGGCUAUU | Translation | Nucleic acid binding protein |
| ptc-miR475b-5p | *SlAlba4* | 3.5 | -1 | 1 | 21 | 273 | 293 | AAUGGCCAUUGUAAGAGUAGA | UCUGCUCUUGGGAUGGCUAUU | Translation | Nucleic acid binding protein |
| ptc-miR480 | *SlAlba4* | 5 | -1 | 1 | 24 | 738 | 761 | ACUACUACAUCAUUGACGUUGAAC | CUUCACUGUAAGUGAUGUGGUAAU | Cleavage | Nucleic acid binding protein |
| ptc-miR6456 | *SlAlba4* | 5 | -1 | 1 | 21 | 117 | 136 | UUGAGUCCUUCCAUUAGAUCC | AAAUC-GAUGGAAGGAAUCAC | Cleavage | Nucleic acid binding protein |
| ptc-miR6457b | *SlAlba4* | 4 | -1 | 1 | 21 | 75 | 95 | UUAGUUUGGCAGCCUCUUCUC | GAAAGGAGGCUGCGAAAUUAG | Cleavage | Nucleic acid binding protein |
| rgl-miR7972 | *SlAlba4* | 5 | -1 | 1 | 21 | 434 | 454 | UUGUCAGGCUUGUUAUUCUCC | CAAGACUGAGAAGUUUGAUGA | Cleavage | Nucleic acid binding protein |
| smo-miR1103-3p | *SlAlba4* | 5 | -1 | 1 | 24 | 667 | 690 | UGGAAAAAGGAGGUGCAUUCUUGU | AUGUGUAUGCCUUUUUUUUUUUUA | Cleavage | Nucleic acid binding protein |
| stu-miR8029 | *SlAlba4* | 5 | -1 | 1 | 24 | 618 | 641 | AGCCAUUUUUCUUUGUUUUGGAGC | UUUUAAAAAAAAAGCAGCAUGGUU | Translation | Nucleic acid binding protein |
| tae-miR6201 | *SlAlba4* | 4.5 | -1 | 1 | 22 | 149 | 170 | UGACCCUGAGGCACUCAUACCG | CAAGAUGAGUGUCUCAGAGACA | Cleavage | Nucleic acid binding protein |
| vvi-miR479 | *SlAlba4* | 3.5 | -1 | 1 | 22 | 388 | 409 | UGUGGUAUUGGUUCGGCUCAUC | CAGGAGCCCGACCGAUACCAAA | Cleavage | Nucleic acid binding protein |
| zma-miR171h-5p | *SlAlba4* | 5 | -1 | 1 | 21 | 387 | 407 | UGGUAUUGUUUCGGCUCAUGU | CCAGGAGCCCGACCGAUACCA | Cleavage | Nucleic acid binding protein |
| zma-miR171k-5p | *SlAlba4* | 5 | -1 | 1 | 21 | 387 | 407 | UGGUAUUGUUUCGGCUCAUGU | CCAGGAGCCCGACCGAUACCA | Cleavage | Nucleic acid binding protein |
| ahy-miR156a | *SlAlba5* | 5 | -1 | 1 | 20 | 659 | 678 | UGACAGAAGAGAGAGAGCAC | CUUCUCCCUCUCUUUUGCCU | Cleavage | Nucleic acid binding protein |
| aly-miR156a-5p | *SlAlba5* | 5 | -1 | 1 | 20 | 659 | 678 | UGACAGAAGAGAGUGAGCAC | CUUCUCCCUCUCUUUUGCCU | Cleavage | Nucleic acid binding protein |
| aly-miR3446-3p | *SlAlba5* | 5 | -1 | 1 | 24 | 132 | 155 | UGCCACUCUUUUAGCUUCCGAAUC | UUGCAGAGAGUUAAAAGAGUUGAA | Cleavage | Nucleic acid binding protein |
| aqc-miR529 | *SlAlba5* | 4 | -1 | 1 | 21 | 654 | 674 | AGAAGAGAGAGAGCACAACCC | AAGUUCUUCUCCCUCUCUUUU | Translation | Nucleic acid binding protein |
| ata-miR156a-5p | *SlAlba5* | 5 | -1 | 1 | 20 | 659 | 678 | UGACAGAAGAGAGUGAGCAC | CUUCUCCCUCUCUUUUGCCU | Cleavage | Nucleic acid binding protein |
| ata-miR156b-5p | *SlAlba5* | 5 | -1 | 1 | 20 | 659 | 678 | UGACAGAAGAGAGUGAGCAC | CUUCUCCCUCUCUUUUGCCU | Cleavage | Nucleic acid binding protein |
| ata-miR156d-3p | *SlAlba5* | 4.5 | -1 | 1 | 22 | 163 | 184 | GCUCACUCCUCUUUCUGUCAGC | AGAGUUAGCAGAAGGAGUGAGC | Translation | Nucleic acid binding protein |
| ata-miR156d-5p | *SlAlba5* | 5 | -1 | 1 | 20 | 659 | 678 | UGACAGAAGAGAGUGAGCAC | CUUCUCCCUCUCUUUUGCCU | Cleavage | Nucleic acid binding protein |
| ata-miR156e-5p | *SlAlba5* | 5 | -1 | 1 | 20 | 659 | 678 | UGACAGAAGAGAGUGAGCAC | CUUCUCCCUCUCUUUUGCCU | Cleavage | Nucleic acid binding protein |
| ath-miR5017-5p | *SlAlba5* | 5 | -1 | 1 | 21 | 578 | 598 | AUUUGUUACUAAUUUGGAAUG | CGUUGUAAUUUAGAAGCAAAU | Cleavage | Nucleic acid binding protein |
| ath-miR5657 | *SlAlba5* | 5 | -1 | 1 | 21 | 715 | 735 | UGGACAAGGUUAGAUUUGGUG | AACUGAAUUUUACAUUGUCUA | Translation | Nucleic acid binding protein |
| ath-miR844-5p | *SlAlba5* | 4.5 | -1 | 1 | 21 | 584 | 604 | UGGUAAGAUUGCUUAUAAGCU | AAUUUAGAAGCAAAUUUACCU | Cleavage | Nucleic acid binding protein |
| ath-miR869.1 | *SlAlba5* | 5 | -1 | 1 | 21 | 527 | 547 | AUUGGUUCAAUUCUGGUGUUG | CAGGAACAGAGUUGAAUAAAU | Cleavage | Nucleic acid binding protein |
| bdi-miR156a | *SlAlba5* | 5 | -1 | 1 | 21 | 658 | 678 | UGACAGAAGAGAGAGAGCACA | UCUUCUCCCUCUCUUUUGCCU | Cleavage | Nucleic acid binding protein |
| bdi-miR156b-5p | *SlAlba5* | 5 | -1 | 1 | 20 | 659 | 678 | UGACAGAAGAGAGUGAGCAC | CUUCUCCCUCUCUUUUGCCU | Cleavage | Nucleic acid binding protein |
| bdi-miR156c | *SlAlba5* | 5 | -1 | 1 | 20 | 659 | 678 | UGACAGAAGAGAGUGAGCAC | CUUCUCCCUCUCUUUUGCCU | Cleavage | Nucleic acid binding protein |
| bdi-miR5176-3p | *SlAlba5* | 5 | -1 | 1 | 21 | 730 | 750 | UGUGAUGAUGUGGCAUAGAAU | UGUCUAUUUCGAGUCGUUACA | Translation | Nucleic acid binding protein |
| bdi-miR529-5p | *SlAlba5* | 4.5 | -1 | 1 | 21 | 654 | 674 | AGAAGAGAGAGAGUACAGCCU | AAGUUCUUCUCCCUCUCUUUU | Translation | Nucleic acid binding protein |
| bdi-miR7717a-3p | *SlAlba5* | 5 | -1 | 1 | 24 | 323 | 346 | GAUGGAUACGAUUGUCGACUGAGA | GCGAUUUCAACAGUUGUAUCAAUU | Cleavage | Nucleic acid binding protein |
| bdi-miR7717c-3p | *SlAlba5* | 4.5 | -1 | 1 | 24 | 97 | 120 | UUAGUUGACUGAGAAAUAGACGGU | UCCCUCCAUUGCUCAGUUGACGAA | Cleavage | Nucleic acid binding protein |
| bdi-miR7725b-5p.1 | *SlAlba5* | 5 | -1 | 1 | 21 | 4 | 24 | ACUAGGGAGAUGGUUUUCGCU | UACAAAUUCUGUUUUCCUAGU | Cleavage | Nucleic acid binding protein |
| bdi-miR7748b-3p | *SlAlba5* | 4.5 | -1 | 1 | 24 | 631 | 654 | UUGGUCAAAGAAAAUCUAAUACGC | UCUCUACAGGUUUUCUUUGUCUAA | Cleavage | Nucleic acid binding protein |
| bdi-miR7767-5p | *SlAlba5* | 3.5 | -1 | 1 | 21 | 298 | 318 | CCCCAAGCUGAGAGCUCUCCC | AGUGGAGCUUUCAGCUUUGGG | Cleavage | Nucleic acid binding protein |
| bna-miR156a | *SlAlba5* | 5 | -1 | 1 | 21 | 658 | 678 | UGACAGAAGAGAGUGAGCACA | UCUUCUCCCUCUCUUUUGCCU | Cleavage | Nucleic acid binding protein |
| bna-miR156d | *SlAlba5* | 5 | -1 | 1 | 20 | 659 | 678 | UGACAGAAGAGAGUGAGCAC | CUUCUCCCUCUCUUUUGCCU | Cleavage | Nucleic acid binding protein |
| bna-miR156e | *SlAlba5* | 5 | -1 | 1 | 20 | 659 | 678 | UGACAGAAGAGAGUGAGCAC | CUUCUCCCUCUCUUUUGCCU | Cleavage | Nucleic acid binding protein |
| bra-miR6032-3p | *SlAlba5* | 5 | -1 | 1 | 22 | 153 | 174 | UCUGCUGGUCGUUCCAUGUUAA | GAAAUAUGGAAGAGUUAGCAGA | Translation | Nucleic acid binding protein |
| cca-miR156a | *SlAlba5* | 5 | -1 | 1 | 20 | 659 | 678 | UGACAGAAGAGAGUGAGCAC | CUUCUCCCUCUCUUUUGCCU | Cleavage | Nucleic acid binding protein |
| cca-miR156b | *SlAlba5* | 5 | -1 | 1 | 21 | 658 | 678 | UGACAGAAGAGAGUGAGCAUA | UCUUCUCCCUCUCUUUUGCCU | Cleavage | Nucleic acid binding protein |
| cpa-miR8136 | *SlAlba5* | 5 | -1 | 1 | 19 | 1 | 19 | UAAAGUGGAAUUGGGAUAA | UUAUACAAAUUCUGUUUUC | Cleavage | Nucleic acid binding protein |
| cre-miR1167 | *SlAlba5* | 4.5 | -1 | 1 | 20 | 735 | 754 | GGGGUGUGAUGAUUUGAAAC | AUUUCGAGUCGUUACACCAG | Cleavage | Nucleic acid binding protein |
| csi-miR156 | *SlAlba5* | 5 | -1 | 1 | 20 | 659 | 678 | UGACAGAAGAGAGUGAGCAC | CUUCUCCCUCUCUUUUGCCU | Cleavage | Nucleic acid binding protein |
| csi-miR3946 | *SlAlba5* | 4.5 | -1 | 1 | 24 | 616 | 639 | UUGUAGAGAAAGAGAAGAGAGCAC | AUUAUCCUUUUUUGUUCUCUACAG | Translation | Nucleic acid binding protein |
| ctr-miR156 | *SlAlba5* | 5 | -1 | 1 | 20 | 659 | 678 | UGACAGAAGAGAGUGAGCAC | CUUCUCCCUCUCUUUUGCCU | Cleavage | Nucleic acid binding protein |
| dpr-miR156a | *SlAlba5* | 4 | -1 | 1 | 20 | 659 | 678 | UGACAGAAGAGAGGGAGCAC | CUUCUCCCUCUCUUUUGCCU | Cleavage | Nucleic acid binding protein |
| dpr-miR156b | *SlAlba5* | 5 | -1 | 1 | 20 | 659 | 678 | UGACAGAAGAGAGUGAGCAC | CUUCUCCCUCUCUUUUGCCU | Cleavage | Nucleic acid binding protein |
| esi-miR3455-3p | *SlAlba5* | 4.5 | -1 | 1 | 21 | 558 | 578 | CGGUGGAAUCUGAGGAUAGUG | UAAUGUUCUCAGAUUCUGUCC | Cleavage | Nucleic acid binding protein |
| far-miR1119 | *SlAlba5* | 5 | -1 | 1 | 24 | 57 | 80 | UGGCACGGCGUGAUGCUUAGUCAG | ACAGUUAAUUAUCCCGCCGUUUCA | Translation | Nucleic acid binding protein |
| far-miR156a | *SlAlba5* | 5 | -1 | 1 | 21 | 658 | 678 | UGACAGAAGAGAGAGAGCACA | UCUUCUCCCUCUCUUUUGCCU | Cleavage | Nucleic acid binding protein |
| far-miR529 | *SlAlba5* | 4.5 | -1 | 1 | 21 | 654 | 674 | AGAAGAGAGAGAGCACAGCUU | AAGUUCUUCUCCCUCUCUUUU | Translation | Nucleic acid binding protein |
| ghr-miR156a | *SlAlba5* | 5 | -1 | 1 | 20 | 659 | 678 | UGACAGAAGAGAGUGAGCAC | CUUCUCCCUCUCUUUUGCCU | Cleavage | Nucleic acid binding protein |
| ghr-miR156b | *SlAlba5* | 5 | -1 | 1 | 20 | 659 | 678 | UGACAGAAGAGAGUGAGCAC | CUUCUCCCUCUCUUUUGCCU | Cleavage | Nucleic acid binding protein |
| gma-miR1510a-3p | *SlAlba5* | 5 | -1 | 1 | 23 | 457 | 479 | UUGUUGUUUUACCUAUUCCACCC | AAUCGUACUGGGUAAGACGGUGA | Cleavage | Nucleic acid binding protein |
| gma-miR156a | *SlAlba5* | 5 | -1 | 1 | 20 | 659 | 678 | UGACAGAAGAGAGUGAGCAC | CUUCUCCCUCUCUUUUGCCU | Cleavage | Nucleic acid binding protein |
| gma-miR156b | *SlAlba5* | 5 | -1 | 1 | 21 | 658 | 678 | UGACAGAAGAGAGAGAGCACA | UCUUCUCCCUCUCUUUUGCCU | Cleavage | Nucleic acid binding protein |
| gma-miR5035-3p | *SlAlba5* | 5 | -1 | 1 | 24 | 696 | 718 | UGUUUAGAAGCUCAUAGAAUAGAU | CUCU-UUUUUUGGGUGUCUAAACU | Cleavage | Nucleic acid binding protein |
| gra-miR8657a | *SlAlba5* | 4.5 | -1 | 1 | 24 | 389 | 412 | UGUAGUAAUUGUAGAAGUUCAGGG | AUCAUGACUUCUACGGUUGAUGUA | Cleavage | Nucleic acid binding protein |
| gra-miR8657b | *SlAlba5* | 4.5 | -1 | 1 | 24 | 389 | 412 | UGUAGUAAUUGUAGAAGUUCAGGG | AUCAUGACUUCUACGGUUGAUGUA | Cleavage | Nucleic acid binding protein |
| gra-miR8691 | *SlAlba5* | 5 | -1 | 1 | 24 | 612 | 635 | AGAUGAUGAGAAAGGAAAGUCAAG | CUAUAUUAUCCUUUUUUGUUCUCU | Cleavage | Nucleic acid binding protein |
| gra-miR8698 | *SlAlba5* | 4.5 | -1 | 1 | 24 | 49 | 72 | AGGGACAAUUAACUUUAACGGUCA | CAAAAGUAACAGUUAAUUAUCCCG | Cleavage | Nucleic acid binding protein |
| gra-miR8746 | *SlAlba5* | 5 | -1 | 1 | 21 | 143 | 162 | UCCAUAUUUCACUAUCUCUUA | UAAAAGA-GUUGAAAUAUGGA | Cleavage | Nucleic acid binding protein |
| gra-miR8751a | *SlAlba5* | 4.5 | -1 | 1 | 24 | 621 | 645 | UGAAAUUUGUAGAG-ACAAAACGCU | CCUUUUUUGUUCUCUACAGGUUUUC | Cleavage | Nucleic acid binding protein |
| gra-miR8751b | *SlAlba5* | 5 | -1 | 1 | 24 | 622 | 645 | UGAAAUUUGUAGAGAGAUAACGCU | CUUUUUUGUUCUCUACAGGUUUUC | Cleavage | Nucleic acid binding protein |
| han-miR156a | *SlAlba5* | 5 | -1 | 1 | 20 | 659 | 678 | UGACAGAAGAGAGUGAGCAC | CUUCUCCCUCUCUUUUGCCU | Cleavage | Nucleic acid binding protein |
| han-miR156b | *SlAlba5* | 5 | -1 | 1 | 20 | 659 | 678 | UGACAGAAGAGAGUGAGCAC | CUUCUCCCUCUCUUUUGCCU | Cleavage | Nucleic acid binding protein |
| hvu-miR6192 | *SlAlba5* | 4.5 | -1 | 1 | 22 | 654 | 675 | UAGGAGAGGGGGGAAGGGAUCU | AAGUUCUUCUCCCUCUCUUUUG | Cleavage | Nucleic acid binding protein |
| hvu-miR6205 | *SlAlba5* | 5 | -1 | 1 | 23 | 211 | 233 | AGGAUGUUUGGAUACGUUUUAGU | GAAGAAUCGGAUUCAAGUAUCUA | Cleavage | Nucleic acid binding protein |
| lja-miR7533a | *SlAlba5* | 5 | -1 | 1 | 21 | 653 | 673 | GAGGGGAUGGAGAGAAGCUGG | AAAGUUCUUCUCCCUCUCUUU | Cleavage | Nucleic acid binding protein |
| lja-miR7533b | *SlAlba5* | 5 | -1 | 1 | 21 | 653 | 673 | GAGGGGAUGGAGAGAAGCUGG | AAAGUUCUUCUCCCUCUCUUU | Cleavage | Nucleic acid binding protein |
| mdm-miR156a | *SlAlba5* | 5 | -1 | 1 | 20 | 659 | 678 | UGACAGAAGAGAGUGAGCAC | CUUCUCCCUCUCUUUUGCCU | Cleavage | Nucleic acid binding protein |
| mdm-miR156b | *SlAlba5* | 5 | -1 | 1 | 20 | 659 | 678 | UGACAGAAGAGAGUGAGCAC | CUUCUCCCUCUCUUUUGCCU | Cleavage | Nucleic acid binding protein |
| mdm-miR5225a | *SlAlba5* | 5 | -1 | 1 | 22 | 70 | 91 | UCUGUCGAAGGUGAGAUGGUGC | CCGCCGUUUCAUUUUCAGCGGG | Cleavage | Nucleic acid binding protein |
| mdm-miR5225b | *SlAlba5* | 5 | -1 | 1 | 22 | 70 | 91 | UCUGUCGAAGGUGAGAUGGUGC | CCGCCGUUUCAUUUUCAGCGGG | Cleavage | Nucleic acid binding protein |
| mes-miR156a | *SlAlba5* | 5 | -1 | 1 | 20 | 659 | 678 | UGACAGAAGAGAGUGAGCAC | CUUCUCCCUCUCUUUUGCCU | Cleavage | Nucleic acid binding protein |
| mes-miR156b | *SlAlba5* | 5 | -1 | 1 | 20 | 659 | 678 | UGACAGAAGAGAGUGAGCAC | CUUCUCCCUCUCUUUUGCCU | Cleavage | Nucleic acid binding protein |
| mtr-miR1507-5p | *SlAlba5* | 5 | -1 | 1 | 22 | 622 | 643 | AGAGUUGUAUGGAACGAAAGAU | CUUUUUUGUUCUCUACAGGUUU | Translation | Nucleic acid binding protein |
| mtr-miR156a | *SlAlba5* | 5 | -1 | 1 | 21 | 658 | 678 | UGACAGAAGAGAGAGAGCACA | UCUUCUCCCUCUCUUUUGCCU | Cleavage | Nucleic acid binding protein |
| mtr-miR156b-5p | *SlAlba5* | 5 | -1 | 1 | 20 | 659 | 678 | UGACAGAAGAGAGUGAGCAC | CUUCUCCCUCUCUUUUGCCU | Cleavage | Nucleic acid binding protein |
| mtr-miR156i-3p | *SlAlba5* | 5 | -1 | 1 | 23 | 163 | 185 | UGCUCACUUCUCUUUCUGUCAUC | AGAGUUAGCAGAAGGAGUGAGCA | Cleavage | Nucleic acid binding protein |
| mtr-miR156i-5p | *SlAlba5* | 5 | -1 | 1 | 20 | 659 | 678 | UGACAGAAGAGAGUGAGCAC | CUUCUCCCUCUCUUUUGCCU | Cleavage | Nucleic acid binding protein |
| mtr-miR2590h | *SlAlba5* | 4.5 | -1 | 1 | 24 | 61 | 84 | AGAAUGACAUGGCAGAAUAAUCAC | UUAAUUAUCCCGCCGUUUCAUUUU | Cleavage | Nucleic acid binding protein |
| mtr-miR2592a.2-3p | *SlAlba5* | 5 | -1 | 1 | 21 | 212 | 232 | AAAUGCUUGAGUCCUGUUGUU | AAGAAUCGGAUUCAAGUAUCU | Cleavage | Nucleic acid binding protein |
| mtr-miR2618a | *SlAlba5* | 5 | -1 | 1 | 22 | 705 | 726 | GUGAAUUCAGUUUACGUACGUU | UGGGUGUCUAAACUGAAUUUUA | Cleavage | Nucleic acid binding protein |
| mtr-miR2618b | *SlAlba5* | 5 | -1 | 1 | 22 | 705 | 726 | GUGAAUUCAGUUUACGUACGUU | UGGGUGUCUAAACUGAAUUUUA | Cleavage | Nucleic acid binding protein |
| mtr-miR5225a | *SlAlba5* | 5 | -1 | 1 | 22 | 387 | 408 | UCAGUCGCAGGAGAGAUGACAC | AAAUCAUGACUUCUACGGUUGA | Cleavage | Nucleic acid binding protein |
| mtr-miR5250 | *SlAlba5* | 5 | -1 | 1 | 21 | 548 | 568 | UGAGAAUGUUAGAUACGGAAC | AUAUUGUAGUUAAUGUUCUCA | Cleavage | Nucleic acid binding protein |
| mtr-miR5256 | *SlAlba5* | 4 | -1 | 1 | 21 | 718 | 738 | UAAUGGAUUAUGUAAGAUUAA | UGAAUUUUACAUUGUCUAUUU | Cleavage | Nucleic acid binding protein |
| mtr-miR5272f | *SlAlba5* | 5 | -1 | 1 | 24 | 181 | 204 | GAAUUGAUUAUGUUUGGAUACACU | GAGCAAUUUGAACCUGGUUGAUUC | Translation | Nucleic acid binding protein |
| mtr-miR7698-5p | *SlAlba5* | 5 | -1 | 1 | 21 | 472 | 492 | UUUUCAUCAAAGUUUUCUGGA | GACGGUGAACUUUGAUGAGUU | Cleavage | Nucleic acid binding protein |
| nta-miR156a | *SlAlba5* | 5 | -1 | 1 | 20 | 659 | 678 | UGACAGAAGAGAGUGAGCAC | CUUCUCCCUCUCUUUUGCCU | Cleavage | Nucleic acid binding protein |
| osa-miR1440b | *SlAlba5* | 4.5 | -1 | 1 | 21 | 4 | 24 | UUUAGGAGAGUGGUAUUUGAG | UACAAAUUCUGUUUUCCUAGU | Cleavage | Nucleic acid binding protein |
| osa-miR156a | *SlAlba5* | 5 | -1 | 1 | 20 | 659 | 678 | UGACAGAAGAGAGUGAGCAC | CUUCUCCCUCUCUUUUGCCU | Cleavage | Nucleic acid binding protein |
| osa-miR156f-3p | *SlAlba5* | 5 | -1 | 1 | 22 | 163 | 184 | GCUCACUUCUCUUUCUGUCAGC | AGAGUUAGCAGAAGGAGUGAGC | Translation | Nucleic acid binding protein |
| osa-miR156i | *SlAlba5* | 5 | -1 | 1 | 20 | 659 | 678 | UGACAGAAGAGAGUGAGCAC | CUUCUCCCUCUCUUUUGCCU | Cleavage | Nucleic acid binding protein |
| osa-miR156j-3p | *SlAlba5* | 5 | -1 | 1 | 22 | 163 | 184 | GCUCGCUCCUCUUUCUGUCAGC | AGAGUUAGCAGAAGGAGUGAGC | Translation | Nucleic acid binding protein |
| osa-miR156j-5p | *SlAlba5* | 5 | -1 | 1 | 20 | 659 | 678 | UGACAGAAGAGAGUGAGCAC | CUUCUCCCUCUCUUUUGCCU | Cleavage | Nucleic acid binding protein |
| osa-miR156k | *SlAlba5* | 5 | -1 | 1 | 21 | 658 | 678 | UGACAGAAGAGAGAGAGCACA | UCUUCUCCCUCUCUUUUGCCU | Cleavage | Nucleic acid binding protein |
| osa-miR156l-3p | *SlAlba5* | 5 | -1 | 1 | 22 | 163 | 184 | GCUCACUUCUCUUUCUGUCAGC | AGAGUUAGCAGAAGGAGUGAGC | Translation | Nucleic acid binding protein |
| osa-miR156l-5p | *SlAlba5* | 5 | -1 | 1 | 21 | 658 | 678 | CGACAGAAGAGAGUGAGCAUA | UCUUCUCCCUCUCUUUUGCCU | Cleavage | Nucleic acid binding protein |
| osa-miR529b | *SlAlba5* | 4.5 | -1 | 1 | 21 | 654 | 674 | AGAAGAGAGAGAGUACAGCUU | AAGUUCUUCUCCCUCUCUUUU | Translation | Nucleic acid binding protein |
| osa-miR5821 | *SlAlba5* | 5 | -1 | 1 | 21 | 96 | 116 | UGGACGGAGCGAUGGUGGGCG | GUCCCUCCAUUGCUCAGUUGA | Cleavage | Nucleic acid binding protein |
| ppt-miR156a | *SlAlba5* | 5 | -1 | 1 | 20 | 659 | 678 | UGACAGAAGAGAGUGAGCAC | CUUCUCCCUCUCUUUUGCCU | Cleavage | Nucleic acid binding protein |
| ppt-miR529d | *SlAlba5* | 4.5 | -1 | 1 | 21 | 654 | 674 | AGAAGAGAGAGAGCACAGCCC | AAGUUCUUCUCCCUCUCUUUU | Translation | Nucleic acid binding protein |
| ptc-miR156a | *SlAlba5* | 5 | -1 | 1 | 20 | 659 | 678 | UGACAGAAGAGAGUGAGCAC | CUUCUCCCUCUCUUUUGCCU | Cleavage | Nucleic acid binding protein |
| ptc-miR7814 | *SlAlba5* | 4.5 | -1 | 1 | 21 | 506 | 526 | UAGAUUGUUUUUAUGCUUUGA | CAAAAGGAUGGAAACAAUAUU | Cleavage | Nucleic acid binding protein |
| rco-miR156a | *SlAlba5* | 5 | -1 | 1 | 21 | 658 | 678 | UGACAGAAGAGAGUGAGCACA | UCUUCUCCCUCUCUUUUGCCU | Cleavage | Nucleic acid binding protein |
| rgl-miR5576 | *SlAlba5* | 5 | -1 | 1 | 23 | 309 | 331 | AGAAGUUGGCAUUUGCAAACACU | CAGCUUUGGGAAUGGCGAUUUCA | Cleavage | Nucleic acid binding protein |
| sbi-miR156a | *SlAlba5* | 5 | -1 | 1 | 20 | 659 | 678 | UGACAGAAGAGAGUGAGCAC | CUUCUCCCUCUCUUUUGCCU | Cleavage | Nucleic acid binding protein |
| sly-miR156d-5p | *SlAlba5* | 5 | -1 | 1 | 20 | 659 | 678 | UGACAGAAGAGAGUGAGCAC | CUUCUCCCUCUCUUUUGCCU | Cleavage | Nucleic acid binding protein |
| sly-miR9471a-3p | *SlAlba5* | 5 | -1 | 1 | 21 | 489 | 509 | UUGGCUGAGUGAGCAUCACGG | AGUUGAUGCUCGCUGGGCAAA | Cleavage | Nucleic acid binding protein |
| sly-miR9471b-3p | *SlAlba5* | 5 | -1 | 1 | 21 | 489 | 509 | UUGGCUGAGUGAGCAUCACUG | AGUUGAUGCUCGCUGGGCAAA | Cleavage | Nucleic acid binding protein |
| sly-miR9474-5p | *SlAlba5* | 4.5 | -1 | 1 | 22 | 382 | 403 | UGUAGAAGUCAUGAAUAAAAUG | AAAAAAAAUCAUGACUUCUACG | Cleavage | Nucleic acid binding protein |
| smo-miR156a | *SlAlba5* | 5 | -1 | 1 | 20 | 659 | 678 | CGACAGAAGAGAGUGAGCAC | CUUCUCCCUCUCUUUUGCCU | Cleavage | Nucleic acid binding protein |
| stu-miR4376-3p | *SlAlba5* | 4 | -1 | 1 | 20 | 278 | 297 | GCAUCAUACUCCUGCAUAUU | UAUAUGCAGCAGUAUAAUGA | Translation | Nucleic acid binding protein |
| stu-miR7122-5p | *SlAlba5* | 5 | -1 | 1 | 22 | 634 | 654 | UUAUACAGAGAAACCGCUGUCG | CUACAG-GUUUUCUUUGUCUAA | Cleavage | Nucleic acid binding protein |
| stu-miR7991a | *SlAlba5* | 5 | -1 | 1 | 21 | 1 | 21 | AGGAGGUCGGAAUUUUUAAUG | UUAUACAAAUUCUGUUUUCCU | Cleavage | Nucleic acid binding protein |
| stu-miR8031 | *SlAlba5* | 4 | -1 | 1 | 23 | 693 | 715 | UUAGACACCUCAACUAAGACUUG | GCCCUCUUUUUUUGGGUGUCUAA | Translation | Nucleic acid binding protein |
| stu-miR8038a-3p | *SlAlba5* | 4 | -1 | 1 | 21 | 173 | 193 | GUUCAACUUGCUCACUUGGAG | GAAGGAGUGAGCAAUUUGAAC | Cleavage | Nucleic acid binding protein |
| stu-miR8038b-3p | *SlAlba5* | 4 | -1 | 1 | 21 | 173 | 193 | GUUCAACUUGCUCACUUGGAG | GAAGGAGUGAGCAAUUUGAAC | Cleavage | Nucleic acid binding protein |
| tae-miR156 | *SlAlba5* | 5 | -1 | 1 | 21 | 658 | 678 | UGACAGAAGAGAGUGAGCACA | UCUUCUCCCUCUCUUUUGCCU | Cleavage | Nucleic acid binding protein |
| vvi-miR3624-5p | *SlAlba5* | 5 | -1 | 1 | 21 | 276 | 296 | UAGUAUGCUGCUGUCUUUAGA | GGUAUAUGCAGCAGUAUAAUG | Cleavage | Nucleic acid binding protein |
| zma-miR156a-5p | *SlAlba5* | 5 | -1 | 1 | 20 | 659 | 678 | UGACAGAAGAGAGUGAGCAC | CUUCUCCCUCUCUUUUGCCU | Cleavage | Nucleic acid binding protein |
| zma-miR156d-3p | *SlAlba5* | 5 | -1 | 1 | 22 | 163 | 184 | GCUCACUUCUCUUUCUGUCAGC | AGAGUUAGCAGAAGGAGUGAGC | Translation | Nucleic acid binding protein |
| zma-miR156d-5p | *SlAlba5* | 5 | -1 | 1 | 20 | 659 | 678 | UGACAGAAGAGAGUGAGCAC | CUUCUCCCUCUCUUUUGCCU | Cleavage | Nucleic acid binding protein |
| zma-miR156f-3p | *SlAlba5* | 5 | -1 | 1 | 22 | 163 | 184 | GCUCACUUCUCUUUCUGUCAGC | AGAGUUAGCAGAAGGAGUGAGC | Translation | Nucleic acid binding protein |
| zma-miR156f-5p | *SlAlba5* | 5 | -1 | 1 | 20 | 659 | 678 | UGACAGAAGAGAGUGAGCAC | CUUCUCCCUCUCUUUUGCCU | Cleavage | Nucleic acid binding protein |
| zma-miR156g-3p | *SlAlba5* | 5 | -1 | 1 | 22 | 163 | 184 | GCUCACUUCUCUUUCUGUCAGC | AGAGUUAGCAGAAGGAGUGAGC | Translation | Nucleic acid binding protein |
| zma-miR156g-5p | *SlAlba5* | 5 | -1 | 1 | 20 | 659 | 678 | UGACAGAAGAGAGUGAGCAC | CUUCUCCCUCUCUUUUGCCU | Cleavage | Nucleic acid binding protein |
| zma-miR529-5p | *SlAlba5* | 4.5 | -1 | 1 | 21 | 654 | 674 | AGAAGAGAGAGAGUACAGCCU | AAGUUCUUCUCCCUCUCUUUU | Translation | Nucleic acid binding protein |
| aly-miR3449-5p | *SlAlba6* | 5 | -1 | 1 | 21 | 1023 | 1043 | UCUAGAUUGCUUUCGGUCUGU | CAAGUUCAAGUGCAAUUUAGA | Translation | Ribonuclease P protein subunit p25 |
| aly-miR400-3p | *SlAlba6* | 4 | -1 | 1 | 21 | 953 | 973 | GACUUAUAAUAAUCUCAUGAA | AGCAUGAAAUUAUUAUGGCUU | Cleavage | Ribonuclease P protein subunit p25 |
| aly-miR4235 | *SlAlba6* | 5 | -1 | 1 | 21 | 48 | 68 | ACUGAAAACACUACAACCCCU | GAGGGUUUUAGGGUUUUUGUU | Translation | Ribonuclease P protein subunit p25 |
| aly-miR472-3p | *SlAlba6* | 4 | -1 | 1 | 22 | 198 | 219 | UUUUUCCUACUCCGCCCAUACC | UUCAGGAGAGGAGUGGGAAAGA | Cleavage | Ribonuclease P protein subunit p25 |
| aly-miR834-5p | *SlAlba6* | 5 | -1 | 1 | 21 | 785 | 805 | ACCACAGCUUCUGCUACGAAC | GGAGGUGGCAGAGGUUAUGGU | Cleavage | Ribonuclease P protein subunit p25 |
| aly-miR837-5p | *SlAlba6* | 5 | -1 | 1 | 21 | 946 | 966 | CAUUGUUUCUUGUUUUUUUCA | UAAAAAAAGCAUGAAAUUAUU | Translation | Ribonuclease P protein subunit p25 |
| aly-miR838-3p | *SlAlba6* | 3.5 | -1 | 1 | 21 | 29 | 49 | UUUUCUUCUUCUUCUUGCACA | CUCUGAAGAAGAAGAAGAAGA | Cleavage | Ribonuclease P protein subunit p25 |
| aly-miR862-3p | *SlAlba6* | 5 | -1 | 1 | 21 | 373 | 393 | ACAUGCUGGAUCUACUUGAAG | UGUGGAGCAGACUCGGCAUGU | Translation | Ribonuclease P protein subunit p25 |
| aqc-miR482a | *SlAlba6* | 5 | -1 | 1 | 22 | 198 | 219 | UCUUGCCGACUCCUCCCAUACC | UUCAGGAGAGGAGUGGGAAAGA | Cleavage | Ribonuclease P protein subunit p25 |
| ath-miR838 | *SlAlba6* | 5 | -1 | 1 | 21 | 29 | 49 | UUUUCUUCUACUUCUUGCACA | CUCUGAAGAAGAAGAAGAAGA | Translation | Ribonuclease P protein subunit p25 |
| bdi-miR166h-5p | *SlAlba6* | 5 | -1 | 1 | 21 | 234 | 254 | GGUUUGUUGUCUGGCUCGGGG | CAAUGGGUCAGGCAAUAAGCA | Cleavage | Ribonuclease P protein subunit p25 |
| bdi-miR5163a-3p | *SlAlba6* | 5 | -1 | 1 | 21 | 1055 | 1075 | UUAGGUAUUUCAGGUUAGGUG | UGUUUAUCUUAAAAUGUCUAA | Translation | Ribonuclease P protein subunit p25 |
| bdi-miR5182 | *SlAlba6* | 5 | -1 | 1 | 21 | 991 | 1011 | UGAUGAUCUUGGAACACGUGC | UCUUGUUUUCCAGCAUCAUUA | Cleavage | Ribonuclease P protein subunit p25 |
| bdi-miR5199 | *SlAlba6* | 5 | -1 | 1 | 21 | 312 | 332 | UGUUCAUACGGUUGAUAGCAC | CUGCUAUCAGCUCAGUGAGCA | Cleavage | Ribonuclease P protein subunit p25 |
| bdi-miR7710-3p | *SlAlba6* | 5 | -1 | 1 | 24 | 961 | 984 | AUUGAUGUCACAAACUAUAGUAGC | AUUAUUAUGGCUUGUUGCAUCGAC | Cleavage | Ribonuclease P protein subunit p25 |
| bdi-miR7717c-5p | *SlAlba6* | 4.5 | -1 | 1 | 24 | 829 | 852 | UUGCUAUUUCUUGGGCGACUGAGA | GGGCAACCGUCCAAGGGGUGGCAA | Cleavage | Ribonuclease P protein subunit p25 |
| bdi-miR827-5p | *SlAlba6* | 5 | -1 | 1 | 22 | 678 | 699 | UUUUGUUGGUUGUCAUCUAACC | AUGGAUAUGAUAACUAUCAAGA | Cleavage | Ribonuclease P protein subunit p25 |
| bra-miR400-3p | *SlAlba6* | 4.5 | -1 | 1 | 21 | 953 | 973 | GACUUAUAAUGAUCUCAUGAA | AGCAUGAAAUUAUUAUGGCUU | Cleavage | Ribonuclease P protein subunit p25 |
| bra-miR5716 | *SlAlba6* | 5 | -1 | 1 | 21 | 1055 | 1075 | UUGGAUAAUUGAAGAUAUAAA | UGUUUAUCUUAAAAUGUCUAA | Translation | Ribonuclease P protein subunit p25 |
| bra-miR9561-3p | *SlAlba6* | 5 | -1 | 1 | 22 | 45 | 66 | GAGAGACUCUGAAAGACUCACC | GAAGAGGGUUUUAGGGUUUUUG | Cleavage | Ribonuclease P protein subunit p25 |
| csi-miR472 | *SlAlba6* | 5 | -1 | 1 | 21 | 198 | 218 | UUUUCCCACACCUCCCAUCCC | UUCAGGAGAGGAGUGGGAAAG | Translation | Ribonuclease P protein subunit p25 |
| csi-miR482b | *SlAlba6* | 5 | -1 | 1 | 22 | 198 | 219 | UCUUGCCCACCCCUCCCAUUCC | UUCAGGAGAGGAGUGGGAAAGA | Translation | Ribonuclease P protein subunit p25 |
| dpr-miR160 | *SlAlba6* | 5 | -1 | 1 | 21 | 227 | 247 | UGCCUGGCUCCUUGUAUGCCA | UUGAAAGCAAUGGGUCAGGCA | Translation | Ribonuclease P protein subunit p25 |
| ghr-miR482a | *SlAlba6* | 2.5 | -1 | 1 | 22 | 198 | 219 | UCUUUCCUACUCCUCCCAUACC | UUCAGGAGAGGAGUGGGAAAGA | Cleavage | Ribonuclease P protein subunit p25 |
| gma-miR1510a-3p | *SlAlba6* | 4.5 | -1 | 1 | 23 | 229 | 251 | UUGUUGUUUUACCUAUUCCACCC | GAAAGCAAUGGGUCAGGCAAUAA | Translation | Ribonuclease P protein subunit p25 |
| gma-miR1510a-5p | *SlAlba6* | 5 | -1 | 1 | 24 | 1042 | 1065 | AGGGAUAGGUAAAACAAUGACUGC | GACCAUAUUCUUUUGUUUAUCUUA | Cleavage | Ribonuclease P protein subunit p25 |
| gma-miR1512a-5p | *SlAlba6* | 5 | -1 | 1 | 22 | 4 | 25 | UAACUGAAAAUUCUUAAAGUAU | UUCUGUUAAGGGUUUACAGUUG | Cleavage | Ribonuclease P protein subunit p25 |
| gma-miR1512b | *SlAlba6* | 5 | -1 | 1 | 22 | 4 | 25 | UAACUGGAAAUUCUUAAAGCAU | UUCUGUUAAGGGUUUACAGUUG | Cleavage | Ribonuclease P protein subunit p25 |
| gma-miR1513c | *SlAlba6* | 5 | -1 | 1 | 19 | 916 | 934 | UAUGAGAGAAAGCCAUGAC | AUAAUGGUUUUCUUUUGUC | Cleavage | Ribonuclease P protein subunit p25 |
| gma-miR1530 | *SlAlba6* | 5 | -1 | 1 | 21 | 58 | 78 | UUUUCACAUAAAUUAAAAUAU | GGGUUUUUGUUUAUCAGAAAA | Cleavage | Ribonuclease P protein subunit p25 |
| gma-miR171h | *SlAlba6* | 4 | -1 | 1 | 21 | 379 | 399 | AUUGAGACGAGCCGAAUCAAU | GCAGACUCGGCAUGUCUCAAU | Translation | Ribonuclease P protein subunit p25 |
| gma-miR393b | *SlAlba6* | 4 | -1 | 1 | 21 | 746 | 766 | UUUGGGAUCAUGCUAUCCCUU | UGGGGAUAUCGUGAUUCUGGA | Cleavage | Ribonuclease P protein subunit p25 |
| gma-miR4396 | *SlAlba6* | 5 | -1 | 1 | 24 | 153 | 176 | UGUAGUUUCUAAGACGAUGCUGAC | CACAGGGACUUGUUAGAAACUACA | Cleavage | Ribonuclease P protein subunit p25 |
| gma-miR4416c | *SlAlba6* | 4.5 | -1 | 1 | 21 | 917 | 937 | CUGGGUGAGAGAAACACGUAU | UAAUGGUUUUCUUUUGUCCAG | Cleavage | Ribonuclease P protein subunit p25 |
| gma-miR5033 | *SlAlba6* | 4 | -1 | 1 | 21 | 919 | 939 | GGCUGUACAAAAGGAAACUAC | AUGGUUUUCUUUUGUCCAGUG | Cleavage | Ribonuclease P protein subunit p25 |
| gma-miR5780a | *SlAlba6* | 4.5 | -1 | 1 | 23 | 308 | 330 | AUCACUUAGCUGACGGUAGGGAC | GAUGCUGCUAUCAGCUCAGUGAG | Cleavage | Ribonuclease P protein subunit p25 |
| gma-miR9760 | *SlAlba6* | 4.5 | -1 | 1 | 21 | 163 | 183 | UGGAUGAUGUAGUUUUGAUUG | UGUUAGAAACUACAUCAGCUA | Cleavage | Ribonuclease P protein subunit p25 |
| gra-miR482 | *SlAlba6* | 4 | -1 | 1 | 22 | 198 | 219 | UCUUUCCAAUUCCUCCCAUUCC | UUCAGGAGAGGAGUGGGAAAGA | Cleavage | Ribonuclease P protein subunit p25 |
| gra-miR8742a | *SlAlba6* | 4 | -1 | 1 | 21 | 669 | 689 | UAUCUUAUUCAUCUUGGACUG | AUUACCAAGAUGGAUAUGAUA | Cleavage | Ribonuclease P protein subunit p25 |
| gra-miR8742b | *SlAlba6* | 4 | -1 | 1 | 21 | 669 | 689 | UAUCUUAUUCAUCUUGGACUG | AUUACCAAGAUGGAUAUGAUA | Cleavage | Ribonuclease P protein subunit p25 |
| gra-miR8782 | *SlAlba6* | 5 | -1 | 1 | 21 | 288 | 308 | UUUGGUGUUGAAGGGGAAUAA | GAAUUCCUCGUCUGCAUCAAG | Cleavage | Ribonuclease P protein subunit p25 |
| hci-miR156a | *SlAlba6* | 5 | -1 | 1 | 20 | 916 | 935 | UGACAGAAGAGAGUGAGUAC | AUAAUGGUUUUCUUUUGUCC | Cleavage | Ribonuclease P protein subunit p25 |
| hme-miR-1b | *SlAlba6* | 5 | -1 | 1 | 22 | 981 | 1002 | UGGGAAGUAAGGAAGCACGGAA | CGACGGGUUGUCUUGUUUUCCA | Cleavage | Ribonuclease P protein subunit p25 |
| mdm-miR482b | *SlAlba6* | 4.5 | -1 | 1 | 22 | 198 | 219 | UCUUUCCUAUCCCUCCCAUUCC | UUCAGGAGAGGAGUGGGAAAGA | Translation | Ribonuclease P protein subunit p25 |
| mtr-miR1510a-5p | *SlAlba6* | 5 | -1 | 1 | 21 | 228 | 248 | UUGUCUUACCCAUUCCUCCCA | UGAAAGCAAUGGGUCAGGCAA | Cleavage | Ribonuclease P protein subunit p25 |
| mtr-miR2597 | *SlAlba6* | 5 | -1 | 1 | 21 | 76 | 95 | UUUGGUACUUCGUCGAUUUGA | AAAAAUGGAU-AGGUACCAAA | Translation | Ribonuclease P protein subunit p25 |
| mtr-miR2620 | *SlAlba6* | 4.5 | -1 | 1 | 22 | 55 | 76 | UUCUGAUAGACACCGGCUCUGC | UUAGGGUUUUUGUUUAUCAGAA | Cleavage | Ribonuclease P protein subunit p25 |
| mtr-miR2636 | *SlAlba6* | 5 | -1 | 1 | 21 | 465 | 485 | UUUGGUUAGUGUGCUGAAUAU | AUAUUCAGCAGACUAAUUACU | Translation | Ribonuclease P protein subunit p25 |
| mtr-miR2673a | *SlAlba6* | 2.5 | -1 | 1 | 22 | 30 | 51 | CCUCUUCCUCUUCCUCUUCCAC | UCUGAAGAAGAAGAAGAAGAGG | Cleavage | Ribonuclease P protein subunit p25 |
| mtr-miR2673b | *SlAlba6* | 2.5 | -1 | 1 | 22 | 30 | 51 | CCUCUUCCUCUUCCUCUUCCAC | UCUGAAGAAGAAGAAGAAGAGG | Cleavage | Ribonuclease P protein subunit p25 |
| mtr-miR393b-3p | *SlAlba6* | 4 | -1 | 1 | 21 | 746 | 766 | UUUGGGAUCAUGCUAUCCCUU | UGGGGAUAUCGUGAUUCUGGA | Cleavage | Ribonuclease P protein subunit p25 |
| mtr-miR5744 | *SlAlba6* | 4 | -1 | 1 | 22 | 1053 | 1074 | UAGGUAUUUUAAGGAGCACGUU | UUUGUUUAUCUUAAAAUGUCUA | Cleavage | Ribonuclease P protein subunit p25 |
| mtr-miR5757 | *SlAlba6* | 4.5 | -1 | 1 | 21 | 866 | 886 | UAGAGAUUUGUUUAACAGCCA | UAGUUGUUGGUUGGAUCUCUA | Translation | Ribonuclease P protein subunit p25 |
| nta-miR6156 | *SlAlba6* | 5 | -1 | 1 | 21 | 211 | 231 | UUGAAGAUGUUCUAUUUCUGU | UGGGAAAGAGAUCAUUUUGAA | Translation | Ribonuclease P protein subunit p25 |
| osa-miR1438 | *SlAlba6* | 5 | -1 | 1 | 22 | 947 | 968 | AGGGUAAUUUUAUCAUUUUUAA | AAAAAAAGCAUGAAAUUAUUAU | Cleavage | Ribonuclease P protein subunit p25 |
| osa-miR166k-5p | *SlAlba6* | 5 | -1 | 1 | 21 | 234 | 254 | GGUUUGUUGUCUGGCUCGAGG | CAAUGGGUCAGGCAAUAAGCA | Cleavage | Ribonuclease P protein subunit p25 |
| osa-miR1872 | *SlAlba6* | 4.5 | -1 | 1 | 24 | 2 | 25 | GAACUGUAAGUCUGUGACGGGUAA | GUUUCUGUUAAGGGUUUACAGUUG | Cleavage | Ribonuclease P protein subunit p25 |
| osa-miR1882a | *SlAlba6* | 4.5 | -1 | 1 | 24 | 216 | 239 | AGAUUGCUUUCAAGGUCAUUUCUU | AAGAGAUCAUUUUGAAAGCAAUGG | Cleavage | Ribonuclease P protein subunit p25 |
| osa-miR5822 | *SlAlba6* | 5 | -1 | 1 | 21 | 458 | 478 | UGUCUGCUCGAUGUCAGGUUG | CCUUCUGAUAUUCAGCAGACU | Cleavage | Ribonuclease P protein subunit p25 |
| osa-miR5830 | *SlAlba6* | 5 | -1 | 1 | 24 | 281 | 304 | AUGUAGAUGAGGUGAUGUUACACA | AAGAGAAGAAUUCCUCGUCUGCAU | Cleavage | Ribonuclease P protein subunit p25 |
| osa-miR815a | *SlAlba6* | 4.5 | -1 | 1 | 21 | 394 | 414 | AAGGGGAUUGAGGAGAUUGGG | CUCAAUGAUUUCAAUCACCUU | Cleavage | Ribonuclease P protein subunit p25 |
| pab-miR482a | *SlAlba6* | 4 | -1 | 1 | 22 | 198 | 219 | UCUUCCCUACUCCUCCCAUUCC | UUCAGGAGAGGAGUGGGAAAGA | Cleavage | Ribonuclease P protein subunit p25 |
| pde-miR396 | *SlAlba6* | 5 | -1 | 1 | 21 | 195 | 215 | UCCCACGGCUUUCUUGAACUU | UGCUUCAGGAGAGGAGUGGGA | Cleavage | Ribonuclease P protein subunit p25 |
| pde-miR482a | *SlAlba6* | 2.5 | -1 | 1 | 22 | 198 | 219 | UCUUUCCUACUCCUCCCAUUCC | UUCAGGAGAGGAGUGGGAAAGA | Cleavage | Ribonuclease P protein subunit p25 |
| pde-miR482b | *SlAlba6* | 4.5 | -1 | 1 | 22 | 198 | 219 | UCUUCCCUAUUCCUCCCAUUCC | UUCAGGAGAGGAGUGGGAAAGA | Cleavage | Ribonuclease P protein subunit p25 |
| ppt-miR1069-3p | *SlAlba6* | 5 | -1 | 1 | 21 | 53 | 73 | UGAUAAAUCAAAGUGCUCACU | UUUUAGGGUUUUUGUUUAUCA | Cleavage | Ribonuclease P protein subunit p25 |
| ppt-miR1075 | *SlAlba6* | 4.5 | -1 | 1 | 21 | 633 | 653 | UGUUUCAGUCAUGGUUUCUAC | GCAGGGGACGUGGUUGGAACA | Cleavage | Ribonuclease P protein subunit p25 |
| ppt-miR160g | *SlAlba6* | 5 | -1 | 1 | 21 | 227 | 247 | UGCCUGGCUCCUUGUAUGCCA | UUGAAAGCAAUGGGUCAGGCA | Translation | Ribonuclease P protein subunit p25 |
| pta-miR482a | *SlAlba6* | 4 | -1 | 1 | 22 | 198 | 219 | UCUUCCCUACUCCUCCCAUUCC | UUCAGGAGAGGAGUGGGAAAGA | Cleavage | Ribonuclease P protein subunit p25 |
| pta-miR482b | *SlAlba6* | 4 | -1 | 1 | 22 | 198 | 219 | UCUUCCCUACUCCUCCCAUUCC | UUCAGGAGAGGAGUGGGAAAGA | Cleavage | Ribonuclease P protein subunit p25 |
| pta-miR482c | *SlAlba6* | 4.5 | -1 | 1 | 20 | 200 | 219 | UCUUCCCUAUUCCUCCCAUU | CAGGAGAGGAGUGGGAAAGA | Cleavage | Ribonuclease P protein subunit p25 |
| ptc-miR472b | *SlAlba6* | 5 | -1 | 1 | 22 | 776 | 797 | UUUUCCCAACUCCACCCAUCCC | GUCAGGGGUGGAGGUGGCAGAG | Cleavage | Ribonuclease P protein subunit p25 |
| ptc-miR482a.2 | *SlAlba6* | 4 | -1 | 1 | 20 | 200 | 219 | UCUUGCCUACUCCUCCCAUU | CAGGAGAGGAGUGGGAAAGA | Cleavage | Ribonuclease P protein subunit p25 |
| ptc-miR482c-3p | *SlAlba6* | 5 | -1 | 1 | 22 | 198 | 219 | UCUUUCCGAGUCCUCCCAUACC | UUCAGGAGAGGAGUGGGAAAGA | Translation | Ribonuclease P protein subunit p25 |
| sbi-miR5568d-5p | *SlAlba6* | 5 | -1 | 1 | 21 | 759 | 779 | UGGCUUUUCUAGAUACAUAGC | AUUCUGGAUAUGGAAGAGUCA | Cleavage | Ribonuclease P protein subunit p25 |
| sbi-miR5568g-5p | *SlAlba6* | 4.5 | -1 | 1 | 21 | 1062 | 1082 | CAAAUUAUAAGAUGUUUUGGC | CUUAAAAUGUCUAAUCAUUUG | Cleavage | Ribonuclease P protein subunit p25 |
| sly-miR482e-3p | *SlAlba6* | 2.5 | -1 | 1 | 22 | 198 | 219 | UCUUUCCUACUCCUCCCAUACC | UUCAGGAGAGGAGUGGGAAAGA | Cleavage | Ribonuclease P protein subunit p25 |
| sly-miR9471b-5p | *SlAlba6* | 4.5 | -1 | 1 | 21 | 316 | 336 | GAGGUGCUCACUCAGCUAAUA | UAUCAGCUCAGUGAGCAUCAC | Cleavage | Ribonuclease P protein subunit p25 |
| smo-miR1103-3p | *SlAlba6* | 5 | -1 | 1 | 24 | 913 | 936 | UGGAAAAAGGAGGUGCAUUCUUGU | GGCAUAAUGGUUUUCUUUUGUCCA | Cleavage | Ribonuclease P protein subunit p25 |
| stu-miR7122-5p | *SlAlba6* | 4.5 | -1 | 1 | 22 | 17 | 38 | UUAUACAGAGAAACCGCUGUCG | UUACAGUUGUUUCUCUGAAGAA | Cleavage | Ribonuclease P protein subunit p25 |
| vvi-miR482 | *SlAlba6* | 2.5 | -1 | 1 | 22 | 198 | 219 | UCUUUCCUACUCCUCCCAUUCC | UUCAGGAGAGGAGUGGGAAAGA | Cleavage | Ribonuclease P protein subunit p25 |
| aly-miR166g-5p | *SlAlba7* | 5 | -1 | 1 | 21 | 449 | 469 | GGAAUGUUGUUUGGCUCGAGG | ACAAGAGCUGAACAACAAUUC | Cleavage | Ribonuclease P protein subunit p25 |
| aly-miR2112-3p | *SlAlba7* | 4.5 | -1 | 1 | 21 | 1116 | 1136 | CUUUAUAUCCGCAUUUGCGCA | GUCGUGACUCUGGAUAUGAAG | Cleavage | Ribonuclease P protein subunit p25 |
| aly-miR4250 | *SlAlba7* | 5 | -1 | 1 | 21 | 386 | 406 | UCCAAAGGCACAAGAACAUCA | AGAGGGUCUUUUGCCUGUGGA | Translation | Ribonuclease P protein subunit p25 |
| aly-miR828-5p | *SlAlba7* | 5 | -1 | 1 | 22 | 586 | 607 | UCUUGCUUAAAUGAGUAUUCCA | AUGAAGAUUCAUAUGGGCGAGG | Translation | Ribonuclease P protein subunit p25 |
| aly-miR837-5p | *SlAlba7* | 5 | -1 | 1 | 21 | 1282 | 1302 | CAUUGUUUCUUGUUUUUUUCA | AAAGAAAAGCAUGAAAUUGUG | Translation | Ribonuclease P protein subunit p25 |
| aly-miR847-5p | *SlAlba7* | 4.5 | -1 | 1 | 21 | 212 | 232 | UCUUGAUGAAGAGGAAUGGAA | UGCUAAUACUCUUCUUCAGGA | Cleavage | Ribonuclease P protein subunit p25 |
| aly-miR859-5p | *SlAlba7* | 3.5 | -1 | 1 | 21 | 96 | 116 | UCUCUCCGUUGUAAAAUCAAA | UAGGGUUUUACAAUGGAUAGA | Cleavage | Ribonuclease P protein subunit p25 |
| ath-miR2112-3p | *SlAlba7* | 4.5 | -1 | 1 | 21 | 1116 | 1136 | CUUUAUAUCCGCAUUUGCGCA | GUCGUGACUCUGGAUAUGAAG | Cleavage | Ribonuclease P protein subunit p25 |
| ath-miR5020c | *SlAlba7* | 4.5 | -1 | 1 | 21 | 24 | 44 | UGGCAUGGAAGAAGGUGAGAC | UGGUUACCUUCUUCCAUCCUU | Cleavage | Ribonuclease P protein subunit p25 |
| ath-miR5641 | *SlAlba7* | 4 | -1 | 1 | 21 | 19 | 39 | UGGAAGAAGAUGAUAGAAUUA | UUAUUUGGUUACCUUCUUCCA | Translation | Ribonuclease P protein subunit p25 |
| ath-miR828 | *SlAlba7* | 5 | -1 | 1 | 22 | 586 | 607 | UCUUGCUUAAAUGAGUAUUCCA | AUGAAGAUUCAUAUGGGCGAGG | Translation | Ribonuclease P protein subunit p25 |
| ath-miR837-5p | *SlAlba7* | 4.5 | -1 | 1 | 21 | 657 | 677 | AUCAGUUUCUUGUUCGUUUCA | GGAAACUAUCAAGAAAAUGGU | Cleavage | Ribonuclease P protein subunit p25 |
| ath-miR859 | *SlAlba7* | 5 | -1 | 1 | 21 | 96 | 116 | UCUCUCUGUUGUGAAGUCAAA | UAGGGUUUUACAAUGGAUAGA | Cleavage | Ribonuclease P protein subunit p25 |
| bdi-miR164a-3p | *SlAlba7* | 5 | -1 | 1 | 21 | 637 | 657 | CAUGUGCCCUUCUUCUCCACC | GUUGGAGCAGAGGUGGAUAUG | Cleavage | Ribonuclease P protein subunit p25 |
| bdi-miR5198 | *SlAlba7* | 4.5 | -1 | 1 | 21 | 1439 | 1459 | GGGGAAAAGAGAUUGAGGGAG | AUAUCUUAAUUUUUUUCCCCC | Cleavage | Ribonuclease P protein subunit p25 |
| bdi-miR7753-5p | *SlAlba7* | 5 | -1 | 1 | 21 | 597 | 617 | AUGUCUUCUUCCUUGCUCAUC | UAUGGGCGAGGACGAGGUCGU | Cleavage | Ribonuclease P protein subunit p25 |
| bdi-miR7786-5p | *SlAlba7* | 5 | -1 | 1 | 24 | 1143 | 1166 | GUCUAUGUCUAUGUCUGUGCACGC | GAGGUUAUGGACGCGGACGUGGAC | Translation | Ribonuclease P protein subunit p25 |
| bdi-miR9493 | *SlAlba7* | 5 | -1 | 1 | 24 | 1237 | 1260 | AAGAAUUAUGAAACGAAGGGAGUA | AGAUGCCUUGGUUUAGUAGUUAUU | Translation | Ribonuclease P protein subunit p25 |
| cme-miR828 | *SlAlba7* | 4.5 | -1 | 1 | 22 | 586 | 607 | UCUUGCUCAAAUGAGUAUUCCA | AUGAAGAUUCAUAUGGGCGAGG | Translation | Ribonuclease P protein subunit p25 |
| csi-miR482a-3p | *SlAlba7* | 4 | -1 | 1 | 22 | 226 | 247 | UCUUCCCUAUGCCUCCCAUUCC | UUCAGGAGAGGCAUGGGAAAGA | Cleavage | Ribonuclease P protein subunit p25 |
| dpr-miR160 | *SlAlba7* | 5 | -1 | 1 | 21 | 255 | 275 | UGCCUGGCUCCUUGUAUGCCA | UUGAAAGCAAUGGGUCAGGCA | Translation | Ribonuclease P protein subunit p25 |
| ghr-miR7507 | *SlAlba7* | 4.5 | -1 | 1 | 24 | 10 | 33 | AAGGUAGUGAAGUAGGCAAUUGGG | GGGGUUAGCUUAUUUGGUUACCUU | Cleavage | Ribonuclease P protein subunit p25 |
| gma-miR166h-5p | *SlAlba7* | 5 | -1 | 1 | 21 | 449 | 469 | GGAAUGUUGUUUGGCUCGAGG | ACAAGAGCUGAACAACAAUUC | Cleavage | Ribonuclease P protein subunit p25 |
| gma-miR166j-5p | *SlAlba7* | 5 | -1 | 1 | 21 | 449 | 469 | GGAAUGUUGUUUGGCUCGAGG | ACAAGAGCUGAACAACAAUUC | Cleavage | Ribonuclease P protein subunit p25 |
| gma-miR4372b | *SlAlba7* | 4.5 | -1 | 1 | 22 | 1309 | 1330 | UAAUAAAAUCGUGACAUGUAAC | UUCAUAUGCAGUGAUUUUAUUG | Cleavage | Ribonuclease P protein subunit p25 |
| gma-miR4395 | *SlAlba7* | 5 | -1 | 1 | 22 | 208 | 229 | UGGAUAGGAGUAUGGGCUUGAG | ACUAUGCUAAUACUCUUCUUCA | Cleavage | Ribonuclease P protein subunit p25 |
| gma-miR4995 | *SlAlba7* | 4.5 | -1 | 1 | 21 | 325 | 345 | AGGCAGUGGCUUGGUUAAGGG | UACUUCAUCAAGACACUGCUA | Cleavage | Ribonuclease P protein subunit p25 |
| gma-miR828a | *SlAlba7* | 4.5 | -1 | 1 | 22 | 586 | 607 | UCUUGCUCAAAUGAGUAUUCCA | AUGAAGAUUCAUAUGGGCGAGG | Translation | Ribonuclease P protein subunit p25 |
| gma-miR828b | *SlAlba7* | 4.5 | -1 | 1 | 22 | 586 | 607 | UCUUGCUCAAAUGAGUAUUCCA | AUGAAGAUUCAUAUGGGCGAGG | Translation | Ribonuclease P protein subunit p25 |
| gma-miR9726 | *SlAlba7* | 4 | -1 | 1 | 22 | 305 | 326 | UAUAGGCAUUAUUUUUUUCUUC | CAUCAAGAGAAGAGUGCCUAUA | Translation | Ribonuclease P protein subunit p25 |
| gma-miR9732 | *SlAlba7* | 5 | -1 | 1 | 21 | 1212 | 1233 | CAAGGGUAUGAUGUG-CAAUCU | GGAUUGGCACAUUGUGUUUUUG | Cleavage | Ribonuclease P protein subunit p25 |
| gma-miR9737 | *SlAlba7* | 4.5 | -1 | 1 | 22 | 420 | 441 | UUGUGGCUGAAAUCACUGUUGC | GUUUCAGUGAUUUCAAUCACGU | Cleavage | Ribonuclease P protein subunit p25 |
| gma-miR9760 | *SlAlba7* | 3.5 | -1 | 1 | 21 | 191 | 211 | UGGAUGAUGUAGUUUUGAUUG | UGUUCGAAACUACAUCAACUA | Cleavage | Ribonuclease P protein subunit p25 |
| gra-miR482 | *SlAlba7* | 5 | -1 | 1 | 22 | 226 | 247 | UCUUUCCAAUUCCUCCCAUUCC | UUCAGGAGAGGCAUGGGAAAGA | Translation | Ribonuclease P protein subunit p25 |
| gra-miR7504n | *SlAlba7* | 5 | -1 | 1 | 24 | 1307 | 1331 | AGGAUAAAAUUACUG-AUGUGGCAU | GUUUCAUAUGCAGUGAUUUUAUUGU | Cleavage | Ribonuclease P protein subunit p25 |
| gra-miR8661 | *SlAlba7* | 5 | -1 | 1 | 21 | 1101 | 1121 | CAUUACUUUUUCAUUCAUUAA | GAGGUGGAUGGGAAGGUCGUG | Cleavage | Ribonuclease P protein subunit p25 |
| gra-miR8722 | *SlAlba7* | 5 | -1 | 1 | 22 | 1100 | 1121 | CAUGUUUUUCCUGUUCAUCUUC | CGAGGUGGAUGGGAAGGUCGUG | Cleavage | Ribonuclease P protein subunit p25 |
| gra-miR8737 | *SlAlba7* | 4.5 | -1 | 1 | 24 | 216 | 239 | GUGUAUCUCCUGAAAACGACGACA | AAUACUCUUCUUCAGGAGAGGCAU | Cleavage | Ribonuclease P protein subunit p25 |
| gra-miR8772 | *SlAlba7* | 4 | -1 | 1 | 21 | 500 | 520 | UUGGACUGUGGCUACAUAUAG | ACAGAUGAAGCCACAGUAUAA | Cleavage | Ribonuclease P protein subunit p25 |
| hvu-miR6180 | *SlAlba7* | 5 | -1 | 1 | 20 | 24 | 43 | AGGGUGGAAGAAAGAGGGCG | UGGUUACCUUCUUCCAUCCU | Cleavage | Ribonuclease P protein subunit p25 |
| hvu-miR6192 | *SlAlba7* | 5 | -1 | 1 | 22 | 44 | 65 | UAGGAGAGGGGGGAAGGGAUCU | UCCUUCCUUCCAUCUUCUCUCA | Translation | Ribonuclease P protein subunit p25 |
| lja-miR7533a | *SlAlba7* | 5 | -1 | 1 | 21 | 42 | 62 | GAGGGGAUGGAGAGAAGCUGG | CUUCCUUCCUUCCAUCUUCUC | Cleavage | Ribonuclease P protein subunit p25 |
| lja-miR7533b | *SlAlba7* | 5 | -1 | 1 | 21 | 42 | 62 | GAGGGGAUGGAGAGAAGCUGG | CUUCCUUCCUUCCAUCUUCUC | Cleavage | Ribonuclease P protein subunit p25 |
| mdm-miR482b | *SlAlba7* | 4 | -1 | 1 | 22 | 226 | 247 | UCUUUCCUAUCCCUCCCAUUCC | UUCAGGAGAGGCAUGGGAAAGA | Translation | Ribonuclease P protein subunit p25 |
| mdm-miR7121a | *SlAlba7* | 5 | -1 | 1 | 21 | 294 | 315 | UCCUCUUGGUGAUCGCC-CUGU | AUAGCGGAGAUCAUCAAGAGAA | Cleavage | Ribonuclease P protein subunit p25 |
| mdm-miR828a | *SlAlba7* | 4.5 | -1 | 1 | 22 | 586 | 607 | UCUUGCUCAAAUGAGUAUUCCA | AUGAAGAUUCAUAUGGGCGAGG | Translation | Ribonuclease P protein subunit p25 |
| mtr-miR1510a-5p | *SlAlba7* | 5 | -1 | 1 | 21 | 256 | 276 | UUGUCUUACCCAUUCCUCCCA | UGAAAGCAAUGGGUCAGGCAA | Cleavage | Ribonuclease P protein subunit p25 |
| mtr-miR2629h | *SlAlba7* | 3.5 | -1 | 1 | 24 | 376 | 399 | GCAGAAGAUCCUCGGCAGUUAACU | AGCCAAUUGAAGAGGGUCUUUUGC | Cleavage | Ribonuclease P protein subunit p25 |
| mtr-miR2675 | *SlAlba7* | 5 | -1 | 1 | 21 | 1226 | 1246 | CGAGGCAUAUUUGCAGGGAUU | UGUUUUUGCACAGAUGCCUUG | Translation | Ribonuclease P protein subunit p25 |
| mtr-miR5212-5p | *SlAlba7* | 4 | -1 | 1 | 22 | 151 | 172 | UGGAUUUCGUAUUUCUUUGGUA | CCAUAAAUGAGUACGAGAUUCG | Cleavage | Ribonuclease P protein subunit p25 |
| mtr-miR5239 | *SlAlba7* | 5 | -1 | 1 | 21 | 47 | 67 | UGGGAGAAAAGAUAGAAUGUG | UUCCUUCCAUCUUCUCUCACA | Cleavage | Ribonuclease P protein subunit p25 |
| mtr-miR5296 | *SlAlba7* | 5 | -1 | 1 | 24 | 56 | 79 | AUUUUGUGUGGGUGUAAGAGGUGU | UCUUCUCUCACACUCGCACACAGA | Cleavage | Ribonuclease P protein subunit p25 |
| mtr-miR5759 | *SlAlba7* | 4 | -1 | 1 | 21 | 1442 | 1462 | AAGGGGGUGAAAAGAUUCAAA | UCUUAAUUUUUUUCCCCCUUU | Cleavage | Ribonuclease P protein subunit p25 |
| nta-miR6154a | *SlAlba7* | 5 | -1 | 1 | 21 | 218 | 238 | UGGGUCUCCUGGAGAAAGGUC | UACUCUUCUUCAGGAGAGGCA | Cleavage | Ribonuclease P protein subunit p25 |
| osa-miR1846a-3p | *SlAlba7* | 5 | -1 | 1 | 21 | 931 | 951 | UGACCCCGUUCUCCUCGCCGG | GGGGCGGAGAAAAUGGGGCCA | Translation | Ribonuclease P protein subunit p25 |
| osa-miR1846b-3p | *SlAlba7* | 5 | -1 | 1 | 21 | 931 | 951 | UGACCCCGUUCUCCUCGCCGG | GGGGCGGAGAAAAUGGGGCCA | Translation | Ribonuclease P protein subunit p25 |
| osa-miR1882a | *SlAlba7* | 4.5 | -1 | 1 | 24 | 244 | 267 | AGAUUGCUUUCAAGGUCAUUUCUU | AAGAGAUUGUCUUGAAAGCAAUGG | Cleavage | Ribonuclease P protein subunit p25 |
| osa-miR2864.1 | *SlAlba7* | 5 | -1 | 1 | 21 | 922 | 942 | UUUUGCUGCCCUUGUUUUGCA | ACUCAAACAGGGGCGGAGAAA | Cleavage | Ribonuclease P protein subunit p25 |
| osa-miR2864.1 | *SlAlba7* | 5 | -1 | 1 | 21 | 1056 | 1076 | UUUUGCUGCCCUUGUUUUGCA | ACUCAAACAGGGGCAGAGGAA | Cleavage | Ribonuclease P protein subunit p25 |
| osa-miR529a | *SlAlba7* | 5 | -1 | 1 | 20 | 1133 | 1152 | CUGUACCCUCUCUCUUCUUC | GAAGGAGGCAGAGGUUAUGG | Cleavage | Ribonuclease P protein subunit p25 |
| osa-miR5340 | *SlAlba7* | 4.5 | -1 | 1 | 23 | 424 | 446 | UGAUGACGUGGAUGAAUUUCAAA | CAGUGAUUUCAAUCACGUUGUCA | Cleavage | Ribonuclease P protein subunit p25 |
| pde-miR396 | *SlAlba7* | 5 | -1 | 1 | 21 | 223 | 243 | UCCCACGGCUUUCUUGAACUU | UUCUUCAGGAGAGGCAUGGGA | Cleavage | Ribonuclease P protein subunit p25 |
| ppt-miR1068 | *SlAlba7* | 5 | -1 | 1 | 21 | 659 | 679 | UAGCCAUUUGCUUGAAGGUCA | AAACUAUCAAGAAAAUGGUGA | Translation | Ribonuclease P protein subunit p25 |
| ppt-miR1075 | *SlAlba7* | 5 | -1 | 1 | 21 | 625 | 645 | UGUUUCAGUCAUGGUUUCUAC | GGAGGGGACGUGGUUGGAGCA | Cleavage | Ribonuclease P protein subunit p25 |
| ppt-miR160g | *SlAlba7* | 5 | -1 | 1 | 21 | 255 | 275 | UGCCUGGCUCCUUGUAUGCCA | UUGAAAGCAAUGGGUCAGGCA | Translation | Ribonuclease P protein subunit p25 |
| ptc-miR156l | *SlAlba7* | 5 | -1 | 1 | 21 | 46 | 66 | UUGACAGAAGAUGGAGAGCAC | CUUCCUUCCAUCUUCUCUCAC | Cleavage | Ribonuclease P protein subunit p25 |
| ptc-miR6449 | *SlAlba7* | 4.5 | -1 | 1 | 21 | 1325 | 1345 | CAUGAUUCUGAAUAACGGUUU | UUAUUGUUUUUCAGCAUCGUG | Cleavage | Ribonuclease P protein subunit p25 |
| ptc-miR828a | *SlAlba7* | 4.5 | -1 | 1 | 22 | 586 | 607 | UCUUGCUCAAAUGAGUAUUCCA | AUGAAGAUUCAUAUGGGCGAGG | Translation | Ribonuclease P protein subunit p25 |
| ptc-miR828b-5p | *SlAlba7* | 4.5 | -1 | 1 | 22 | 586 | 607 | UCUUGCUCAAAUGAGUAUUCCA | AUGAAGAUUCAUAUGGGCGAGG | Translation | Ribonuclease P protein subunit p25 |
| sbi-miR529 | *SlAlba7* | 5 | -1 | 1 | 20 | 1133 | 1152 | CUGUACCCUCUCUCUUCUUC | GAAGGAGGCAGAGGUUAUGG | Cleavage | Ribonuclease P protein subunit p25 |
| sbi-miR6223-5p | *SlAlba7* | 5 | -1 | 1 | 21 | 476 | 496 | UUCUUGGGAGGAGCAUGCUAG | GUAUCAAGCUCCUUCUGAGGU | Cleavage | Ribonuclease P protein subunit p25 |
| sly-miR9473-3p | *SlAlba7* | 5 | -1 | 1 | 21 | 1434 | 1454 | AAACGAGUUCAGAUUUACAGC | UAUGUAUAUCUUAAUUUUUUU | Translation | Ribonuclease P protein subunit p25 |
| ssl-miR828 | *SlAlba7* | 4.5 | -1 | 1 | 22 | 586 | 607 | UCUUGCUCAAAUGAGUAUUCCA | AUGAAGAUUCAUAUGGGCGAGG | Translation | Ribonuclease P protein subunit p25 |
| ssp-miR1128 | *SlAlba7* | 4.5 | -1 | 1 | 21 | 868 | 888 | UACUACUCCCUCCGUCCCAAA | ACUGGGGCGGAGGAAAUGGUG | Cleavage | Ribonuclease P protein subunit p25 |
| stu-miR166c-5p | *SlAlba7* | 5 | -1 | 1 | 21 | 449 | 469 | GGAAUGUUGUUUGGCUCGAGG | ACAAGAGCUGAACAACAAUUC | Cleavage | Ribonuclease P protein subunit p25 |
| stu-miR8036-3p | *SlAlba7* | 4 | -1 | 1 | 22 | 230 | 251 | UAUGUCUUUCCGAUGCCUCCCA | GGAGAGGCAUGGGAAAGAGAUU | Cleavage | Ribonuclease P protein subunit p25 |
| vvi-miR828a | *SlAlba7* | 4.5 | -1 | 1 | 22 | 586 | 607 | UCUUGCUCAAAUGAGUAUUCCA | AUGAAGAUUCAUAUGGGCGAGG | Translation | Ribonuclease P protein subunit p25 |
| zma-miR164c-3p | *SlAlba7* | 5 | -1 | 1 | 21 | 637 | 657 | CAUGUGCCCUUCUUCUCCAUC | GUUGGAGCAGAGGUGGAUAUG | Cleavage | Ribonuclease P protein subunit p25 |
| zma-miR166k-5p | *SlAlba7* | 5 | -1 | 1 | 21 | 449 | 469 | GGAUUGUUGUCUGGCUCGGGG | ACAAGAGCUGAACAACAAUUC | Translation | Ribonuclease P protein subunit p25 |
| zma-miR166n-5p | *SlAlba7* | 5 | -1 | 1 | 21 | 449 | 469 | GGAUUGUUGUCUGGCUCGGUG | ACAAGAGCUGAACAACAAUUC | Translation | Ribonuclease P protein subunit p25 |
| aly-miR169a-3p | *SlAlba8* | 5 | -1 | 1 | 21 | 865 | 885 | GGCAAGUUGUCCUUGGCUACA | UAUAGGCAAGAAGAAUUUGCU | Translation | Ribonuclease P protein subunit p25 |
| aly-miR3434-5p | *SlAlba8* | 4 | -1 | 1 | 21 | 395 | 415 | GCUGAUUCUCUGAUUUUGAAC | AGUCAGGAGCAGAGAAUCAAC | Cleavage | Ribonuclease P protein subunit p25 |
| aly-miR3437-5p | *SlAlba8* | 3.5 | -1 | 1 | 21 | 999 | 1019 | AAAAAACAAGGAUCCACGGAU | UUUGGUUGGUUCUUGUUUUUU | Cleavage | Ribonuclease P protein subunit p25 |
| aly-miR3440-5p | *SlAlba8* | 4.5 | -1 | 1 | 22 | 530 | 551 | UGGUUUCCCUGGCCAAUCCACU | GCAGGGGUGGUUAUGGAAACUA | Cleavage | Ribonuclease P protein subunit p25 |
| aly-miR3446-3p | *SlAlba8* | 4.5 | -1 | 1 | 24 | 779 | 802 | UGCCACUCUUUUAGCUUCCGAAUC | GAGGCGGAGGCUGGGGUGGUGGCA | Cleavage | Ribonuclease P protein subunit p25 |
| aly-miR834-5p | *SlAlba8* | 5 | -1 | 1 | 21 | 730 | 750 | ACCACAGCUUCUGCUACGAAC | GGUCGAGGCAGAGGUUGGGGA | Cleavage | Ribonuclease P protein subunit p25 |
| aly-miR834-5p | *SlAlba8* | 5 | -1 | 1 | 21 | 775 | 795 | ACCACAGCUUCUGCUACGAAC | GGCCGAGGCGGAGGCUGGGGU | Cleavage | Ribonuclease P protein subunit p25 |
| aly-miR838-5p | *SlAlba8* | 5 | -1 | 1 | 21 | 1001 | 1021 | UGCAAGAAUGAGAAGCAAAGC | UGGUUGGUUCUUGUUUUUUCA | Cleavage | Ribonuclease P protein subunit p25 |
| aly-miR847-5p | *SlAlba8* | 5 | -1 | 1 | 21 | 105 | 125 | UCUUGAUGAAGAGGAAUGGAA | UGCCACCACUCUUCUUCAGGA | Cleavage | Ribonuclease P protein subunit p25 |
| aly-miR861-3p | *SlAlba8* | 5 | -1 | 1 | 21 | 220 | 240 | GAUGGAUAUAUCUUCAAGAAC | UUGCAUCAAGAUACAUCUAUC | Cleavage | Ribonuclease P protein subunit p25 |
| ata-miR169d-3p | *SlAlba8* | 5 | -1 | 1 | 21 | 865 | 885 | GGCAAGUUGUCCUUGGCUACA | UAUAGGCAAGAAGAAUUUGCU | Translation | Ribonuclease P protein subunit p25 |
| ath-miR5024-3p | *SlAlba8* | 5 | -1 | 1 | 21 | 479 | 499 | CCGUAUCUUGGCCUUGUCAUU | GUGGUCGAGGCCGAGGUAGGG | Cleavage | Ribonuclease P protein subunit p25 |
| ath-miR774a | *SlAlba8* | 5 | -1 | 1 | 21 | 820 | 840 | UUGGUUACCCAUAUGGCCAUC | CGAGGCCGGAUGGGUGGUCGA | Cleavage | Ribonuclease P protein subunit p25 |
| ath-miR869.1 | *SlAlba8* | 5 | -1 | 1 | 21 | 338 | 358 | AUUGGUUCAAUUCUGGUGUUG | CAACUGCUGAGUUGAAUCAAA | Cleavage | Ribonuclease P protein subunit p25 |
| ath-miR870-5p | *SlAlba8* | 4.5 | -1 | 1 | 21 | 959 | 979 | AAGAACAUCAAAUUAGAAUGU | GCAGUUUUGUUUGAUGUUAUU | Cleavage | Ribonuclease P protein subunit p25 |
| bcy-miR529 | *SlAlba8* | 4.5 | -1 | 1 | 20 | 102 | 121 | GAAGAAGAGAGAUGGUAGAG | CUAUGCCACCACUCUUCUUC | Translation | Ribonuclease P protein subunit p25 |
| bdi-miR169a-3p | *SlAlba8* | 4 | -1 | 1 | 21 | 865 | 885 | GGCGAGUUGUUCUUGGCUACA | UAUAGGCAAGAAGAAUUUGCU | Cleavage | Ribonuclease P protein subunit p25 |
| bdi-miR437 | *SlAlba8* | 5 | -1 | 1 | 21 | 930 | 950 | GAACUUAGAGAAGUUUGACUU | GUGUGAAACUUUUUUGAGUGA | Cleavage | Ribonuclease P protein subunit p25 |
| bdi-miR5200c | *SlAlba8* | 5 | -1 | 1 | 21 | 270 | 290 | UGUAGAUACUCUCUAAGGCUU | GCCCCUUGAAGAGGGUCUACA | Cleavage | Ribonuclease P protein subunit p25 |
| bdi-miR7735-5p | *SlAlba8* | 5 | -1 | 1 | 24 | 411 | 435 | UUGUUUUCCUU-CUGCACUCCCGGC | UCAACAACUGCAGCAAGGAAAACAA | Translation | Ribonuclease P protein subunit p25 |
| bdi-miR7748b-3p | *SlAlba8* | 5 | -1 | 1 | 24 | 963 | 986 | UUGGUCAAAGAAAAUCUAAUACGC | UUUUGUUUGAUGUUAUUUGGCUAA | Translation | Ribonuclease P protein subunit p25 |
| bdi-miR7753-5p | *SlAlba8* | 5 | -1 | 1 | 21 | 832 | 852 | AUGUCUUCUUCCUUGCUCAUC | GGUGGUCGAGGAGGAAGGGGU | Cleavage | Ribonuclease P protein subunit p25 |
| bdi-miR9494 | *SlAlba8* | 5 | -1 | 1 | 21 | 781 | 801 | UUCAUCACCUUCGUCUCCGUC | GGCGGAGGCUGGGGUGGUGGC | Cleavage | Ribonuclease P protein subunit p25 |
| bgy-miR529 | *SlAlba8* | 4.5 | -1 | 1 | 20 | 102 | 121 | GAAGAAGAGAGAUGGUAGAG | CUAUGCCACCACUCUUCUUC | Translation | Ribonuclease P protein subunit p25 |
| cca-miR6118-3p | *SlAlba8* | 5 | -1 | 1 | 22 | 526 | 547 | UUCCGAGGCCACCCAUUCCAAC | CGCGGCAGGGGUGGUUAUGGAA | Cleavage | Ribonuclease P protein subunit p25 |
| esi-miR3453-5p | *SlAlba8* | 3 | -1 | 1 | 21 | 826 | 846 | UUCUCCUCGAUCGCCCGCCUG | CGGAUGGGUGGUCGAGGAGGA | Cleavage | Ribonuclease P protein subunit p25 |
| ghr-miR7496a | *SlAlba8* | 5 | -1 | 1 | 24 | 984 | 1007 | AUGACCAAAUUGAUAGAAUGUGUA | UAAAGGUUCUGUUACUUUGGUUGG | Translation | Ribonuclease P protein subunit p25 |
| ghr-miR7496b | *SlAlba8* | 5 | -1 | 1 | 24 | 984 | 1007 | AUGACCAAAUUGAUAGAAUGUGUA | UAAAGGUUCUGUUACUUUGGUUGG | Translation | Ribonuclease P protein subunit p25 |
| ghr-miR7505 | *SlAlba8* | 5 | -1 | 1 | 21 | 527 | 547 | UUCAGAAACCAUCCCUUCCUU | GCGGCAGGGGUGGUUAUGGAA | Cleavage | Ribonuclease P protein subunit p25 |
| gma-miR1520a | *SlAlba8* | 5 | -1 | 1 | 23 | 1041 | 1063 | UAGAACAUGAUACAUGACAGUCA | UGUCUGUCAUGUAAUAAGUUUUC | Translation | Ribonuclease P protein subunit p25 |
| gma-miR169n-3p | *SlAlba8* | 4 | -1 | 1 | 21 | 869 | 889 | UGCCGGCAAGUUUCUCUUGGC | GGCAAGAAGAAUUUGCUGUCA | Cleavage | Ribonuclease P protein subunit p25 |
| gma-miR396a-3p | *SlAlba8* | 5 | -1 | 1 | 20 | 953 | 972 | UUCAAUAAAGCUGUGGGAAG | UUUCAUGCAGUUUUGUUUGA | Cleavage | Ribonuclease P protein subunit p25 |
| gma-miR396d | *SlAlba8* | 5 | -1 | 1 | 24 | 946 | 969 | AAGAAAGCUGUGGGAGAAUAUGGC | AGUGAAUUUUCAUGCAGUUUUGUU | Cleavage | Ribonuclease P protein subunit p25 |
| gma-miR4386 | *SlAlba8* | 4.5 | -1 | 1 | 24 | 109 | 132 | UCGAAGGUUCUGGAGAGGACUGCA | ACCACUCUUCUUCAGGACCAACGA | Cleavage | Ribonuclease P protein subunit p25 |
| gma-miR9760 | *SlAlba8* | 4.5 | -1 | 1 | 21 | 84 | 104 | UGGAUGAUGUAGUUUUGAUUG | UAUUCGAAAUUACAUUAGCUA | Cleavage | Ribonuclease P protein subunit p25 |
| gra-miR8642 | *SlAlba8* | 5 | -1 | 1 | 24 | 372 | 395 | UGAUCAAAACAGGAACGAAUUCAA | CCAAGCUCCGUCUCGUUUUGAUCA | Translation | Ribonuclease P protein subunit p25 |
| gra-miR8665 | *SlAlba8* | 5 | -1 | 1 | 24 | 649 | 672 | UUAAUUAUUAUAUAGAUCAAGGAU | GAUAAUGAUGGAUAUUAUAAUCAA | Cleavage | Ribonuclease P protein subunit p25 |
| gra-miR8743a | *SlAlba8* | 4.5 | -1 | 1 | 24 | 1043 | 1066 | UAUGAAAAGUUAUAAAAUGGUCAU | UCUGUCAUGUAAUAAGUUUUCAUU | Cleavage | Ribonuclease P protein subunit p25 |
| gra-miR8778 | *SlAlba8* | 5 | -1 | 1 | 24 | 882 | 905 | UUUCCAUAUUAGGGUUUGAACUUU | UGCUGUCAAAGCUUGAUAGGAAAA | Cleavage | Ribonuclease P protein subunit p25 |
| gra-miR8782 | *SlAlba8* | 5 | -1 | 1 | 21 | 110 | 130 | UUUGGUGUUGAAGGGGAAUAA | CCACUCUUCUUCAGGACCAAC | Cleavage | Ribonuclease P protein subunit p25 |
| lja-miR7526a | *SlAlba8* | 5 | -1 | 1 | 21 | 984 | 1004 | AUCAAGGUAGCUGUAACUUCC | UAAAGGUUCUGUUACUUUGGU | Cleavage | Ribonuclease P protein subunit p25 |
| lja-miR7526b | *SlAlba8* | 5 | -1 | 1 | 21 | 984 | 1004 | AUCAAGGUAGCUGUAACUUCC | UAAAGGUUCUGUUACUUUGGU | Cleavage | Ribonuclease P protein subunit p25 |
| lja-miR7531 | *SlAlba8* | 4 | -1 | 1 | 21 | 509 | 529 | CGUGUUUUCUUUCAUUCCCCA | GAGGGCGUGGAAGGGGACGCG | Cleavage | Ribonuclease P protein subunit p25 |
| mtr-miR2612 | *SlAlba8* | 5 | -1 | 1 | 21 | 693 | 713 | UGAUAGUGUCAACUAGUACAG | AGAUAAUGGUGGAUAUUAUCA | Translation | Ribonuclease P protein subunit p25 |
| mtr-miR2670a | *SlAlba8* | 4.5 | -1 | 1 | 21 | 925 | 945 | CAAGAAGGUUGCUCACUAUUU | GCAUGGUGUGAAACUUUUUUG | Translation | Ribonuclease P protein subunit p25 |
| mtr-miR2673a | *SlAlba8* | 4.5 | -1 | 1 | 22 | 836 | 857 | CCUCUUCCUCUUCCUCUUCCAC | GUCGAGGAGGAAGGGGUGGUGG | Cleavage | Ribonuclease P protein subunit p25 |
| mtr-miR2673a | *SlAlba8* | 4.5 | -1 | 1 | 22 | 491 | 512 | CCUCUUCCUCUUCCUCUUCCAC | GAGGUAGGGGCAGAGGCAGAGG | Cleavage | Ribonuclease P protein subunit p25 |
| mtr-miR2673b | *SlAlba8* | 4.5 | -1 | 1 | 22 | 836 | 857 | CCUCUUCCUCUUCCUCUUCCAC | GUCGAGGAGGAAGGGGUGGUGG | Cleavage | Ribonuclease P protein subunit p25 |
| mtr-miR2673b | *SlAlba8* | 4.5 | -1 | 1 | 22 | 491 | 512 | CCUCUUCCUCUUCCUCUUCCAC | GAGGUAGGGGCAGAGGCAGAGG | Cleavage | Ribonuclease P protein subunit p25 |
| mtr-miR2676a | *SlAlba8* | 5 | -1 | 1 | 21 | 545 | 565 | CAUUGUUUGGAUAAUAAUUUG | GAAACUACUAUCAAGAUGAUG | Cleavage | Ribonuclease P protein subunit p25 |
| mtr-miR5289a | *SlAlba8* | 4 | -1 | 1 | 24 | 1051 | 1074 | CGAGGAAAACUGAAAACUUCGGCA | GUAAUAAGUUUUCAUUUUUUUUAG | Translation | Ribonuclease P protein subunit p25 |
| mtr-miR5289b | *SlAlba8* | 4 | -1 | 1 | 24 | 1051 | 1074 | CGAGGAAAACUGAAAACUUCGGCA | GUAAUAAGUUUUCAUUUUUUUUAG | Translation | Ribonuclease P protein subunit p25 |
| osa-miR1426 | *SlAlba8* | 3.5 | -1 | 1 | 21 | 448 | 468 | AGAAUCUUGAUGAUGAUUAAA | UCUGAUUUUAAUCAAGAUUCU | Cleavage | Ribonuclease P protein subunit p25 |
| osa-miR1862d | *SlAlba8* | 5 | -1 | 1 | 24 | 419 | 442 | ACUAGGUUUGUUUAUUUUGGGACG | UGCAGCAAGGAAAACAAGCCUAUG | Cleavage | Ribonuclease P protein subunit p25 |
| osa-miR1871 | *SlAlba8* | 4.5 | -1 | 1 | 24 | 48 | 71 | AUGGCUCUGAUAUCAUGUUGGUUU | UAAGGAGAAUGAGAUCAGAGUCAC | Cleavage | Ribonuclease P protein subunit p25 |
| osa-miR2919 | *SlAlba8* | 4 | -1 | 1 | 19 | 1086 | 1104 | AAGGGGGGGGGGGGAAAGA | UCUUUACUUCACCUCCCUU | Cleavage | Ribonuclease P protein subunit p25 |
| osa-miR395f | *SlAlba8* | 5 | -1 | 1 | 21 | 1011 | 1031 | GUGAAUUGUUUGGGGGAACUC | UUGUUUUUUCAUAUGAUUCAU | Translation | Ribonuclease P protein subunit p25 |
| osa-miR5080 | *SlAlba8* | 5 | -1 | 1 | 21 | 922 | 942 | AAAAGGAUCAUACCGUGACAG | UUGGCAUGGUGUGAAACUUUU | Cleavage | Ribonuclease P protein subunit p25 |
| osa-miR5532 | *SlAlba8* | 5 | -1 | 1 | 22 | 1010 | 1031 | AUGGAAUAUAUGACAAAGGUGG | CUUGUUUUUUCAUAUGAUUCAU | Cleavage | Ribonuclease P protein subunit p25 |
| osa-miR6255 | *SlAlba8* | 5 | -1 | 1 | 22 | 1052 | 1073 | UGGGAAAAAUGGGCAGUUGAGU | UAAUAAGUUUUCAUUUUUUUUA | Cleavage | Ribonuclease P protein subunit p25 |
| pab-miR3702 | *SlAlba8* | 5 | -1 | 1 | 21 | 1 | 21 | AAUCUCUUGGUGCUUAUUCGC | AUGGAUAGGUACCAGAAGGUG | Cleavage | Ribonuclease P protein subunit p25 |
| ppt-miR1046-3p | *SlAlba8* | 5 | -1 | 1 | 21 | 1013 | 1033 | UGGUGAAAAAUAUGAAAAAUC | GUUUUUUCAUAUGAUUCAUUU | Cleavage | Ribonuclease P protein subunit p25 |
| ppt-miR1222d | *SlAlba8* | 4.5 | -1 | 1 | 21 | 259 | 279 | UUUAAGGGGUUCACUGGUAUA | GAUGCAUAUGAGCCCCUUGAA | Cleavage | Ribonuclease P protein subunit p25 |
| ptc-miR169b-3p | *SlAlba8* | 4 | -1 | 1 | 20 | 866 | 885 | GGCAGGUUGUUCUUGGCUAC | AUAGGCAAGAAGAAUUUGCU | Cleavage | Ribonuclease P protein subunit p25 |
| ptc-miR475c | *SlAlba8* | 4.5 | -1 | 1 | 21 | 649 | 668 | UUACAAUGUCCAUUGAUUAAG | GAUAAU-GAUGGAUAUUAUAA | Cleavage | Ribonuclease P protein subunit p25 |
| ptc-miR482d-5p | *SlAlba8* | 5 | -1 | 1 | 22 | 424 | 445 | GGACAUGGGUUGGUUUGCAAGA | CAAGGAAAACAAGCCUAUGUUC | Cleavage | Ribonuclease P protein subunit p25 |
| ptc-miR7836 | *SlAlba8* | 4 | -1 | 1 | 21 | 1087 | 1107 | UGGGUGGGAGGUGUGGUAGCU | CUUUACUUCACCUCCCUUCCA | Cleavage | Ribonuclease P protein subunit p25 |
| ptc-miR7837 | *SlAlba8* | 4 | -1 | 1 | 21 | 1087 | 1107 | UGGGUGGGAGGUGUGGUAGCU | CUUUACUUCACCUCCCUUCCA | Cleavage | Ribonuclease P protein subunit p25 |
| rgl-miR7807a-5p | *SlAlba8* | 5 | -1 | 1 | 21 | 941 | 964 | AACUAUAUGAAAAU---CUCAAUU | UUUUGAGUGAAUUUUCAUGCAGUU | Cleavage | Ribonuclease P protein subunit p25 |
| sbi-miR6231-3p | *SlAlba8* | 5 | -1 | 1 | 21 | 677 | 697 | UAUUUGUGGACUCAUGGACAU | GAGGCGGUGGGUUUGCAGAUA | Cleavage | Ribonuclease P protein subunit p25 |
| sbi-miR6232a-5p | *SlAlba8* | 4 | -1 | 1 | 24 | 706 | 729 | GUCGCUUUGACUUUUUUGGUACAU | UAUUAUCAACAAGGUCAAGGUGGU | Cleavage | Ribonuclease P protein subunit p25 |
| sbi-miR821e | *SlAlba8* | 4.5 | -1 | 1 | 21 | 1026 | 1046 | AAGUCAUCAAAAUAAAAGUUG | AUUCAUUUAUUUUGAUGUCUG | Cleavage | Ribonuclease P protein subunit p25 |
| smo-miR1080 | *SlAlba8* | 5 | -1 | 1 | 22 | 681 | 702 | UUCACUAUCUGCAAACACCUCU | CGGUGGGUUUGCAGAUAAUGGU | Cleavage | Ribonuclease P protein subunit p25 |
| stu-miR8004 | *SlAlba8* | 5 | -1 | 1 | 24 | 900 | 922 | AGGGGUUGUGUAUGUGUUUGGCCU | GGAAAAAAUAU-UGCACAACCCUU | Cleavage | Ribonuclease P protein subunit p25 |
| stu-miR8011a-5p | *SlAlba8* | 5 | -1 | 1 | 24 | 894 | 917 | UUGUGUGAGGUUUCUUUUUGUUUC | UUGAUAGGAAAAAAUAUUGCACAA | Cleavage | Ribonuclease P protein subunit p25 |
| stu-miR8023 | *SlAlba8* | 4 | -1 | 1 | 24 | 129 | 152 | UUUGGCACAAUUUCAUUGGCAACC | ACGAACAAAUGAAAUUGUUUUGAA | Cleavage | Ribonuclease P protein subunit p25 |
| vvi-miR3630-3p | *SlAlba8* | 5 | -1 | 1 | 22 | 183 | 204 | UUUGGGAAUCUCUCUGAUGCAC | UGCCAUUGCAGAGAUUAUUAAG | Cleavage | Ribonuclease P protein subunit p25 |
| vvi-miR3633a-3p | *SlAlba8* | 5 | -1 | 1 | 22 | 823 | 844 | UUCCUAUACCACCCAUUCCCUA | GGCCGGAUGGGUGGUCGAGGAG | Cleavage | Ribonuclease P protein subunit p25 |
| zma-miR169a-3p | *SlAlba8* | 3.5 | -1 | 1 | 21 | 865 | 885 | GGCAAGUUGUUCUUGGCUACA | UAUAGGCAAGAAGAAUUUGCU | Cleavage | Ribonuclease P protein subunit p25 |
| zma-miR169b-3p | *SlAlba8* | 3.5 | -1 | 1 | 21 | 865 | 885 | GGCAAGUUGUUCUUGGCUACA | UAUAGGCAAGAAGAAUUUGCU | Cleavage | Ribonuclease P protein subunit p25 |
| zma-miR169r-3p | *SlAlba8* | 5 | -1 | 1 | 21 | 865 | 885 | GGCAAGUUGUCCUUGGCUACA | UAUAGGCAAGAAGAAUUUGCU | Translation | Ribonuclease P protein subunit p25 |
| zma-miR2118f | *SlAlba8* | 5 | -1 | 1 | 22 | 142 | 163 | UUCCCAAUGCCUUCCAUGCCUA | AUUGUUUUGAAGGCAAUGGGAC | Cleavage | Ribonuclease P protein subunit p25 |

| **Table S11.** Subcellular localization of SlAlba proteins predicted by in silico analysis | | | | | | | | |  |  |  |  |  |  |  |  |  |
| --- | --- | --- | --- | --- | --- | --- | --- | --- | --- | --- | --- | --- | --- | --- | --- | --- | --- |
| **SlAlba1 WoLFPSORT prediction chlo: 10, nucl: 1.5, cysk_nucl: 1.5, cyto: 1, mito: 1** | | | | | | | |  |  |  |  |  |  |  |  |  |  |
| PSORT features and traditional PSORTII prediction | | | | |  |  |  |  |  |  |  |  |  |  |  |  |  |
| 14 Nearest Neighbors | | |  |  |  |  |  |  |  |  |  |  |  |  |  |  |  |
| **No.** | **id** | **site** | **distance** | **identity** | **comments** | |  |  |  |  |  |  |  |  |  |  |  |
| 1 | RCA_SPIOL | chlo | 277.447 | 11.02% | [Uniprot] SWISS-PROT45:Chloroplast stroma. | | | | |  |  |  |  |  |  |  |  |
| 2 | At5g63470.1 | nucl | 278.096 | 12% | [Arath] |  |  |  |  |  |  |  |  |  |  |  |  |
| 3 | RCAA_HORVU | chlo | 278.303 | 10.78% | [Uniprot] SWISS-PROT45:Chloroplast stroma. | | | | |  |  |  |  |  |  |  |  |
| 4 | RCA_LYCPN | chlo | 281.683 | 10.24% | [Uniprot] SWISS-PROT45:Chloroplast stroma. | | | | |  |  |  |  |  |  |  |  |
| 5 | RCA_PHAAU | chlo | 283.085 | 12.07% | [Uniprot] SWISS-PROT45:Chloroplast stroma. | | | | |  |  |  |  |  |  |  |  |
| 6 | ADT1_ARATH | mito | 286.208 | 12.07% | [Uniprot] SWISS-PROT45:Integral membrane protein. Mitochondrial inner membrane. | | | | | | | | |  |  |  |  |
| 7 | CB23_TOBAC | chlo | 288.166 | 12.08% | [Uniprot] SWISS-PROT45:Chloroplast thylakoid membrane. | | | | | |  |  |  |  |  |  |  |
| 8 | CB21_MAIZE | chlo | 296.369 | 12.21% | [Uniprot] SWISS-PROT45:Chloroplast thylakoid membrane. | | | | | |  |  |  |  |  |  |  |
| 9 | RBS1_ORYSA | chlo | 296.729 | 12.21% | [Uniprot] SWISS-PROT45:Chloroplast. | | | |  |  |  |  |  |  |  |  |  |
| 10 | CB21_PETSP | chlo | 300.541 | 12.78% | [Uniprot] SWISS-PROT45:Chloroplast thylakoid membrane. | | | | | |  |  |  |  |  |  |  |
| 11 | At3g51260.1 | cysk_nucl | 303.499 | 12% | [Arath] |  |  |  |  |  |  |  |  |  |  |  |  |
| 12 | RCA_MAIZE | chlo | 305.426 | 12.24% | [Uniprot] SWISS-PROT45:Chloroplast stroma. | | | | |  |  |  |  |  |  |  |  |
| 13 | TRB1_ARATH | cyto | 309.96 | 12.61% | [Uniprot] SWISS-PROT45:Cytoplasmic. | | | |  |  |  |  |  |  |  |  |  |
| 14 | TPIC_SPIOL | chlo | 316.729 | 13.35% | [Uniprot] SWISS-PROT45:Chloroplast. | | | |  |  |  |  |  |  |  |  |  |
|  |  |  |  |  |  |  |  |  |  |  |  |  |  |  |  |  |  |
| **SlAlba2 WoLFPSORT prediction cyto: 9, cyto_nucl: 8.5, nucl: 4, extr: 1** | | | | | | |  |  |  |  |  |  |  |  |  |  |  |
| PSORT features and traditional PSORTII prediction | | | | |  |  |  |  |  |  |  |  |  |  |  |  |  |
| 14 Nearest Neighbors | | |  |  |  |  |  |  |  |  |  |  |  |  |  |  |  |
| **No.** | **id** | **site** | **distance** | **identity** | **comments** | |  |  |  |  |  |  |  |  |  |  |  |
| 1 | RPBY_ARATH | nucl | 187.127 | 12.02% | [Uniprot] SWISS-PROT45:Nuclear. | | | |  |  |  |  |  |  |  |  |  |
| 2 | At1g67990.1 | cyto | 216.781 | 13.87% | [Arath] |  |  |  |  |  |  |  |  |  |  |  |  |
| 3 | At4g27950.1 | nucl | 240.644 | 12.87% | [Arath] |  |  |  |  |  |  |  |  |  |  |  |  |
| 4 | GPD5_ARATH | cyto | 241.906 | 11.63% | [Uniprot] SWISS-PROT45:Cytoplasmic. | | | |  |  |  |  |  |  |  |  |  |
| 5 | UBIQ_ARATH | cyto_nucl | 244.819 | 11.59% | [Uniprot] SWISS-PROT45:Nuclear and cytoplasmic. | | | | |  |  |  |  |  |  |  |  |
| 6 | UBIQ_CHLRE | cyto_nucl | 244.819 | 11.16% | [Uniprot] SWISS-PROT45:Nuclear and cytoplasmic. | | | | |  |  |  |  |  |  |  |  |
| 7 | UBIQ_SOYBN | cyto_nucl | 244.819 | 11.59% | [Uniprot] SWISS-PROT45:Nuclear and cytoplasmic. | | | | |  |  |  |  |  |  |  |  |
| 8 | GPD6_ARATH | cyto | 244.997 | 12.43% | [Uniprot] SWISS-PROT45:Cytoplasmic. | | | |  |  |  |  |  |  |  |  |  |
| 9 | G6PD_SOLTU | cyto | 260.774 | 11.55% | [Uniprot] SWISS-PROT45:Cytoplasmic. | | | |  |  |  |  |  |  |  |  |  |
| 10 | UBIQ_ACECL | cyto_nucl | 262.689 | 11.16% | [Uniprot] SWISS-PROT45:Nuclear and cytoplasmic. | | | | |  |  |  |  |  |  |  |  |
| 11 | ERFC_ARATH | cyto | 280.529 | 14.48% | [Uniprot] SWISS-PROT45:Cytoplasmic. | | | |  |  |  |  |  |  |  |  |  |
| 12 | ERFB_ARATH | cyto | 281.25 | 12.67% | [Uniprot] SWISS-PROT45:Cytoplasmic. | | | |  |  |  |  |  |  |  |  |  |
| 13 | RS3A_CATRO | cyto | 285.156 | 11.92% | [Uniprot] SWISS-PROT45:Cytoplasmic. | | | |  |  |  |  |  |  |  |  |  |
| 14 | E13K_TOBAC | extr | 291.986 | 12.39% | [Uniprot] SWISS-PROT45:Extracellular. | | | |  |  |  |  |  |  |  |  |  |
|  |  |  |  |  |  |  |  |  |  |  |  |  |  |  |  |  |  |
| **SlAlba3 WoLFPSORT prediction cyto_nucl: 7.5, cyto: 7, nucl: 6, pero: 1** | | | | | | |  |  |  |  |  |  |  |  |  |  |  |
| PSORT features and traditional PSORTII prediction | | | | |  |  |  |  |  |  |  |  |  |  |  |  |  |
| 14 Nearest Neighbors | | |  |  |  |  |  |  |  |  |  |  |  |  |  |  |  |
| **No.** | **id** | **site** | **distance** | **identity** | **comments** | |  |  |  |  |  |  |  |  |  |  |  |
| 1 | At4g27950.1 | nucl | 214.747 | 11.98% | [Arath] |  |  |  |  |  |  |  |  |  |  |  |  |
| 2 | RPBY_ARATH | nucl | 243.377 | 11.81% | [Uniprot] SWISS-PROT45:Nuclear. | | | |  |  |  |  |  |  |  |  |  |
| 3 | ERFB_ARATH | cyto | 244.422 | 14.29% | [Uniprot] SWISS-PROT45:Cytoplasmic. | | | |  |  |  |  |  |  |  |  |  |
| 4 | ERFC_ARATH | cyto | 253.8 | 14.94% | [Uniprot] SWISS-PROT45:Cytoplasmic. | | | |  |  |  |  |  |  |  |  |  |
| 5 | G6PD_SOLTU | cyto | 268.771 | 13.11% | [Uniprot] SWISS-PROT45:Cytoplasmic. | | | |  |  |  |  |  |  |  |  |  |
| 6 | At5g53290.1 | nucl | 275.176 | 12.99% | [Arath] |  |  |  |  |  |  |  |  |  |  |  |  |
| 7 | At1g67990.1 | cyto | 277.91 | 13.28% | [Arath] |  |  |  |  |  |  |  |  |  |  |  |  |
| 8 | At2g40340.1 | nucl | 284.302 | 13.74% | [Arath] |  |  |  |  |  |  |  |  |  |  |  |  |
| 9 | GPD6_ARATH | cyto | 285.548 | 13.98% | [Uniprot] SWISS-PROT45:Cytoplasmic. | | | |  |  |  |  |  |  |  |  |  |
| 10 | At1g54830.1 | nucl | 286.108 | 14.51% | [Arath] |  |  |  |  |  |  |  |  |  |  |  |  |
| 11 | GPD5_ARATH | cyto | 287.486 | 11.82% | [Uniprot] SWISS-PROT45:Cytoplasmic. | | | |  |  |  |  |  |  |  |  |  |
| 12 | CAT2_GOSHI | pero | 293.638 | 10.77% | [Uniprot] SWISS-PROT45:Peroxisomal. | | | |  |  |  |  |  |  |  |  |  |
| 13 | UBIQ_ARATH | cyto_nucl | 299.435 | 9.45% | [Uniprot] SWISS-PROT45:Nuclear and cytoplasmic. | | | | |  |  |  |  |  |  |  |  |
| 14 | UBIQ_CHLRE | cyto_nucl | 299.435 | 9.45% | [Uniprot] SWISS-PROT45:Nuclear and cytoplasmic. | | | | |  |  |  |  |  |  |  |  |
|  |  |  |  |  |  |  |  |  |  |  |  |  |  |  |  |  |  |
| **SlAlba4 WoLFPSORT prediction cyto: 6.5, cyto_nucl: 4.83333, chlo: 3, nucl: 2, extr: 2, cysk_nucl: 1.83333** | | | | | | | | | |  |  |  |  |  |  |  |  |
| PSORT features and traditional PSORTII prediction | | | | |  |  |  |  |  |  |  |  |  |  |  |  |  |
| 14 Nearest Neighbors | | |  |  |  |  |  |  |  |  |  |  |  |  |  |  |  |
| **No.** | **id** | **site** | **distance** | **identity** | **comments** | |  |  |  |  |  |  |  |  |  |  |  |
| 1 | RPBY_ARATH | nucl | 131.591 | 13.28% | [Uniprot] SWISS-PROT45:Nuclear. | | | |  |  |  |  |  |  |  |  |  |
| 2 | IPYR_MAIZE | cyto | 256.681 | 12.15% | [Uniprot] SWISS-PROT45:Cytoplasmic. | | | |  |  |  |  |  |  |  |  |  |
| 3 | At1g67990.1 | cyto | 257.674 | 12.45% | [Arath] |  |  |  |  |  |  |  |  |  |  |  |  |
| 4 | KADC_MAIZE | chlo | 261.107 | 15.77% | [Uniprot] SWISS-PROT45:Chloroplast. | | | |  |  |  |  |  |  |  |  |  |
| 5 | ENO_ALNGL | cyto | 262.04 | 8.86% | [Uniprot] SWISS-PROT45:Cytoplasmic. | | | |  |  |  |  |  |  |  |  |  |
| 6 | At1g75950.1 | cysk_nucl | 282.776 | 16.88% | [Arath] |  |  |  |  |  |  |  |  |  |  |  |  |
| 7 | UBIQ_ACECL | cyto_nucl | 284.321 | 9.38% | [Uniprot] SWISS-PROT45:Nuclear and cytoplasmic. | | | | |  |  |  |  |  |  |  |  |
| 8 | RBS1_SPIOL | chlo | 285.177 | 10.94% | [Uniprot] SWISS-PROT45:Chloroplast. | | | |  |  |  |  |  |  |  |  |  |
| 9 | FER2_EQUTE | chlo | 287.332 | 10.94% | [Uniprot] SWISS-PROT45:Chloroplast. | | | |  |  |  |  |  |  |  |  |  |
| 10 | ICI1_PHAAN | extr | 288.678 | 13.28% | [Uniprot] SWISS-PROT45:Secreted. | | | |  |  |  |  |  |  |  |  |  |
| 11 | IPYR_HORVD | cyto | 289.301 | 10.23% | [Uniprot] SWISS-PROT45:Cytoplasmic. | | | |  |  |  |  |  |  |  |  |  |
| 12 | GPD6_ARATH | cyto | 296.598 | 9.51% | [Uniprot] SWISS-PROT45:Cytoplasmic. | | | |  |  |  |  |  |  |  |  |  |
| 13 | MPL3_LOLPR | extr | 297.027 | 17.97% | [Uniprot] SWISS-PROT45:Secreted. | | | |  |  |  |  |  |  |  |  |  |
| 14 | RS3A_CATRO | cyto | 300.46 | 13.90% | [Uniprot] SWISS-PROT45:Cytoplasmic. | | | |  |  |  |  |  |  |  |  |  |
|  |  |  |  |  |  |  |  |  |  |  |  |  |  |  |  |  |  |
| **SlAlba5 WoLFPSORT prediction chlo: 6, cyto: 3.5, cyto_nucl: 3.5, nucl: 2.5, extr: 1, E.R.: 1** | | | | | | | | |  |  |  |  |  |  |  |  |  |
| PSORT features and traditional PSORTII prediction | | | | |  |  |  |  |  |  |  |  |  |  |  |  |  |
| 14 Nearest Neighbors | | |  |  |  |  |  |  |  |  |  |  |  |  |  |  |  |
| **No.** | **id** | **site** | **distance** | **identity** | **comments** | |  |  |  |  |  |  |  |  |  |  |  |
| 1 | At5g39830.1 | chlo | 209.95 | 9.82% | [Arath] Subclass:thylakoid | | |  |  |  |  |  |  |  |  |  |  |
| 2 | IPYR_HORVD | cyto | 237.126 | 11.16% | [Uniprot] SWISS-PROT45:Cytoplasmic. | | | |  |  |  |  |  |  |  |  |  |
| 3 | UDPG_PYRPY | cyto | 241.693 | 8.28% | [Uniprot] SWISS-PROT45:Cytoplasmic. | | | |  |  |  |  |  |  |  |  |  |
| 4 | FER2_EQUAR | chlo | 256.311 | 11.81% | [Uniprot] SWISS-PROT45:Chloroplast. | | | |  |  |  |  |  |  |  |  |  |
| 5 | KADC_MAIZE | chlo | 256.57 | 14.86% | [Uniprot] SWISS-PROT45:Chloroplast. | | | |  |  |  |  |  |  |  |  |  |
| 6 | RBS1_SPIOL | chlo | 260.707 | 10.16% | [Uniprot] SWISS-PROT45:Chloroplast. | | | |  |  |  |  |  |  |  |  |  |
| 7 | FER2_EQUTE | chlo | 267.417 | 11.81% | [Uniprot] SWISS-PROT45:Chloroplast. | | | |  |  |  |  |  |  |  |  |  |
| 8 | UBIQ_ACECL | cyto_nucl | 270.479 | 13.39% | [Uniprot] SWISS-PROT45:Nuclear and cytoplasmic. | | | | |  |  |  |  |  |  |  |  |
| 9 | ICI1_PHAAN | extr | 285.996 | 18.11% | [Uniprot] SWISS-PROT45:Secreted. | | | |  |  |  |  |  |  |  |  |  |
| 10 | LIPC_SOLDE | chlo | 290.347 | 9.51% | [Uniprot] SWISS-PROT45:Chloroplast thylakoid membrane. GO:0009535; C:thylakoid membrane (sensu Viridiplantae); Evidence:ISS | | | | | | | | | | | | |
| 11 | At3g27460.1 | nucl | 291.931 | 11.48% | [Arath] |  |  |  |  |  |  |  |  |  |  |  |  |
| 12 | RPBY_ARATH | nucl | 295.679 | 11.02% | [Uniprot] SWISS-PROT45:Nuclear. | | | |  |  |  |  |  |  |  |  |  |
| 13 | ALF1_PEA | cyto | 296.801 | 9.80% | [Uniprot] SWISS-PROT45:Cytoplasmic. | | | |  |  |  |  |  |  |  |  |  |
| 14 | At1g20575.1 | E.R. | 297.684 | 13.82% | [Arath] |  |  |  |  |  |  |  |  |  |  |  |  |
|  |  |  |  |  |  |  |  |  |  |  |  |  |  |  |  |  |  |
| **SlAlba6 WoLFPSORT prediction cyto: 6.5, cyto_nucl: 6.5, nucl: 5.5, extr: 1, pero: 1** | | | | | | | |  |  |  |  |  |  |  |  |  |  |
| PSORT features and traditional PSORTII prediction | | | | |  |  |  |  |  |  |  |  |  |  |  |  |  |
| 14 Nearest Neighbors | | |  |  |  |  |  |  |  |  |  |  |  |  |  |  |  |
| **No.** | id | site | distance | identity | comments | |  |  |  |  |  |  |  |  |  |  |  |
| 1 | At4g27950.1 | nucl | 224.353 | 12.28% | [Arath] |  |  |  |  |  |  |  |  |  |  |  |  |
| 2 | RPBY_ARATH | nucl | 243.128 | 11.07% | [Uniprot] SWISS-PROT45:Nuclear. | | | |  |  |  |  |  |  |  |  |  |
| 3 | G6PD_SOLTU | cyto | 253.392 | 11.74% | [Uniprot] SWISS-PROT45:Cytoplasmic. | | | |  |  |  |  |  |  |  |  |  |
| 4 | ERFB_ARATH | cyto | 255.246 | 12.90% | [Uniprot] SWISS-PROT45:Cytoplasmic. | | | |  |  |  |  |  |  |  |  |  |
| 5 | ERFC_ARATH | cyto | 273.28 | 13.33% | [Uniprot] SWISS-PROT45:Cytoplasmic. | | | |  |  |  |  |  |  |  |  |  |
| 6 | GPD5_ARATH | cyto | 278.948 | 12.79% | [Uniprot] SWISS-PROT45:Cytoplasmic. | | | |  |  |  |  |  |  |  |  |  |
| 7 | CAT2_GOSHI | pero | 283.563 | 14.23% | [Uniprot] SWISS-PROT45:Peroxisomal. | | | |  |  |  |  |  |  |  |  |  |
| 8 | At2g40340.1 | nucl | 289.067 | 11.73% | [Arath] |  |  |  |  |  |  |  |  |  |  |  |  |
| 9 | GPD6_ARATH | cyto | 290.697 | 12.04% | [Uniprot] SWISS-PROT45:Cytoplasmic. | | | |  |  |  |  |  |  |  |  |  |
| 10 | At5g53290.1 | nucl | 291.954 | 10.73% | [Arath] |  |  |  |  |  |  |  |  |  |  |  |  |
| 11 | At1g67990.1 | cyto | 296.178 | 14.45% | [Arath] |  |  |  |  |  |  |  |  |  |  |  |  |
| 12 | ICI1_PHAAN | extr | 296.416 | 7.63% | [Uniprot] SWISS-PROT45:Secreted. | | | |  |  |  |  |  |  |  |  |  |
| 13 | At1g54830.1 | nucl | 297.506 | 13.36% | [Arath] |  |  |  |  |  |  |  |  |  |  |  |  |
| 14 | UBIQ_ARATH | cyto_nucl | 307.504 | 9.54% | [Uniprot] SWISS-PROT45:Nuclear and cytoplasmic. | | | | |  |  |  |  |  |  |  |  |
|  |  |  |  |  |  |  |  |  |  |  |  |  |  |  |  |  |  |
| **SlAlba7 WoLFPSORT prediction cyto: 5, nucl: 4, chlo: 3, plas: 1, extr: 1** | | | | | | |  |  |  |  |  |  |  |  |  |  |  |
| PSORT features and traditional PSORTII prediction | | | | |  |  |  |  |  |  |  |  |  |  |  |  |  |
| 14 Nearest Neighbors | | |  |  |  |  |  |  |  |  |  |  |  |  |  |  |  |
| **No.** | id | site | distance | identity | comments | |  |  |  |  |  |  |  |  |  |  |  |
| 1 | ICI1_PHAAN | extr | 241.366 | 8.10% | [Uniprot] SWISS-PROT45:Secreted. | | | |  |  |  |  |  |  |  |  |  |
| 2 | ERFB_ARATH | cyto | 252.456 | 13.13% | [Uniprot] SWISS-PROT45:Cytoplasmic. | | | |  |  |  |  |  |  |  |  |  |
| 3 | At5g54110.1 | plas | 253.935 | 13.16% | [Arath] |  |  |  |  |  |  |  |  |  |  |  |  |
| 4 | At4g27950.1 | nucl | 260.181 | 8.35% | [Arath] |  |  |  |  |  |  |  |  |  |  |  |  |
| 5 | FERB_ALOMA | chlo | 266.692 | 8.10% | [Uniprot] SWISS-PROT45:Chloroplast. | | | |  |  |  |  |  |  |  |  |  |
| 6 | At1g03120.1 | nucl | 268.53 | 11.65% | [Arath] |  |  |  |  |  |  |  |  |  |  |  |  |
| 7 | G6PD_SOLTU | cyto | 272.756 | 13.50% | [Uniprot] SWISS-PROT45:Cytoplasmic. | | | |  |  |  |  |  |  |  |  |  |
| 8 | At1g51120.1 | nucl | 273.927 | 11.14% | [Arath] |  |  |  |  |  |  |  |  |  |  |  |  |
| 9 | FERA_ALOMA | chlo | 275.303 | 7.34% | [Uniprot] SWISS-PROT45:Chloroplast. | | | |  |  |  |  |  |  |  |  |  |
| 10 | FER_COLES | chlo | 276.622 | 8.35% | [Uniprot] SWISS-PROT45:Chloroplast. | | | |  |  |  |  |  |  |  |  |  |
| 11 | At2g40340.1 | nucl | 282.196 | 12.15% | [Arath] |  |  |  |  |  |  |  |  |  |  |  |  |
| 12 | ENO_ALNGL | cyto | 285.967 | 10.91% | [Uniprot] SWISS-PROT45:Cytoplasmic. | | | |  |  |  |  |  |  |  |  |  |
| 13 | IPYR_MAIZE | cyto | 288.028 | 11.65% | [Uniprot] SWISS-PROT45:Cytoplasmic. | | | |  |  |  |  |  |  |  |  |  |
| 14 | HS12_ORYSA | cyto | 289.206 | 9.37% | [Uniprot] SWISS-PROT45:Cytoplasmic. | | | |  |  |  |  |  |  |  |  |  |
|  |  |  |  |  |  |  |  |  |  |  |  |  |  |  |  |  |  |
| **SlAlba8 WoLFPSORT prediction nucl: 9, cyto: 4, extr: 1** | | | | | |  |  |  |  |  |  |  |  |  |  |  |  |
| PSORT features and traditional PSORTII prediction | | | | |  |  |  |  |  |  |  |  |  |  |  |  |  |
| 14 Nearest Neighbors | | |  |  |  |  |  |  |  |  |  |  |  |  |  |  |  |
| **No.** | **id** | **site** | **distance** | **identity** | **comments** | |  |  |  |  |  |  |  |  |  |  |  |
| 1 | At1g54830.1 | nucl | 258.737 | 14.38% | [Arath] |  |  |  |  |  |  |  |  |  |  |  |  |
| 2 | RPBY_ARATH | nucl | 271.161 | 11.41% | [Uniprot] SWISS-PROT45:Nuclear. | | | |  |  |  |  |  |  |  |  |  |
| 3 | At4g27950.1 | nucl | 271.661 | 7.19% | [Arath] |  |  |  |  |  |  |  |  |  |  |  |  |
| 4 | ERFB_ARATH | cyto | 277.782 | 15.67% | [Uniprot] SWISS-PROT45:Cytoplasmic. | | | |  |  |  |  |  |  |  |  |  |
| 5 | At5g55160.1 | nucl | 279.529 | 11.07% | [Arath] |  |  |  |  |  |  |  |  |  |  |  |  |
| 6 | At2g40340.1 | nucl | 287.731 | 13.74% | [Arath] |  |  |  |  |  |  |  |  |  |  |  |  |
| 7 | At4g26840.1 | nucl | 288.35 | 9.40% | [Arath] |  |  |  |  |  |  |  |  |  |  |  |  |
| 8 | ERFC_ARATH | cyto | 291.9 | 15.86% | [Uniprot] SWISS-PROT45:Cytoplasmic. | | | |  |  |  |  |  |  |  |  |  |
| 9 | At5g53290.1 | nucl | 300.844 | 9.04% | [Arath] |  |  |  |  |  |  |  |  |  |  |  |  |
| 10 | At4g39780.1 | nucl | 308.928 | 12.04% | [Arath] |  |  |  |  |  |  |  |  |  |  |  |  |
| 11 | ICI1_PHAAN | extr | 312.377 | 8.05% | [Uniprot] SWISS-PROT45:Secreted. | | | |  |  |  |  |  |  |  |  |  |
| 12 | G6PD_SOLTU | cyto | 313.581 | 14.09% | [Uniprot] SWISS-PROT45:Cytoplasmic. | | | |  |  |  |  |  |  |  |  |  |
| 13 | At1g67990.1 | cyto | 316.709 | 14.43% | [Arath] |  |  |  |  |  |  |  |  |  |  |  |  |
| 14 | At1g74930.1 | nucl | 324.148 | 14.09% | [Arath] |  |  |  |  |  |  |  |  |  |  |  |  |
| The top two most similar predicted localization sites are coloured in red. | | | | | | |  |  |  |  |  |  |  |  |  |  |  |

| **Table S13**. Annotated pathways of co-expressed genes | |  |  |  |
| --- | --- | --- | --- | --- |
| **Gene Name** | **GO Term Name** | **MainClass** | **p-value** | **Co-expressed genes** |
| *SlAlba1* | B 09101 Carbohydrate metabolism | A09100 Metabolism | 0.004167 | [Solyc01g009240, Solyc01g005510, Solyc01g066420, Solyc01g056310, Solyc01g010390, Solyc01g096410, Solyc01g066360] |
| *SlAlba1* | 99986 Glycan metabolism | A09190 Not Included in Pathway or Brite | 0.0054095 | [Solyc01g008050] |
| *SlAlba1* | 00460 Cyanoamino acid metabolism | A09100 Metabolism | 0.005674 | [Solyc01g009240, Solyc01g010390] |
| *SlAlba1* | 00053 Ascorbate and aldarate metabolism | A09100 Metabolism | 0.0085951 | [Solyc01g005510, Solyc01g056310] |
| *SlAlba1* | 02000 Transporters | A09180 Brite Hierarchies | 0.0094008 | [Solyc01g008240, Solyc01g087070, Solyc01g068410, Solyc01g091850, Solyc01g081250, Solyc01g066560] |
| *SlAlba1* | B 09183 Protein families: signaling and cellular processes | A09180 Brite Hierarchies | 0.0141336 | [Solyc01g010360, Solyc01g008240, Solyc01g087070, Solyc01g068410, Solyc01g094400, Solyc01g091300, Solyc01g091850, Solyc01g081250, Solyc01g066560] |
| *SlAlba1* | B 09106 Metabolism of other amino acids | A09100 Metabolism | 0.0237072 | [Solyc01g009240, Solyc01g010390, Solyc01g081250] |
| *SlAlba1* | 00040 Pentose and glucuronate interconversions | A09100 Metabolism | 0.0395269 | [Solyc01g066420, Solyc01g066360] |
| *SlAlba1* | 00500 Starch and sucrose metabolism | A09100 Metabolism | 0.0580914 | [Solyc01g009240, Solyc01g010390] |
| *SlAlba1* | 00760 Nicotinate and nicotinamide metabolism | A09100 Metabolism | 0.0605392 | [Solyc01g068630] |
| *SlAlba1* | A09100 Metabolism | A09100 Metabolism | 0.0771657 | [Solyc01g009240, Solyc01g066620, Solyc01g005510, Solyc01g068630, Solyc01g005110, Solyc01g066420, Solyc01g056310, Solyc01g010390, Solyc01g096410, Solyc01g066360, Solyc01g081250] |
| *SlAlba1* | 04130 SNARE interactions in vesicular transport | A09120 Genetic Information Processing | 0.0981085 | [Solyc01g091020] |
| *SlAlba1* | 00510 N-Glycan biosynthesis | A09100 Metabolism | 0.1103079 | [Solyc01g005110] |
| *SlAlba1* | 00940 Phenylpropanoid biosynthesis | A09100 Metabolism | 0.1133896 | [Solyc01g009240, Solyc01g010390] |
| *SlAlba1* | 00592 alpha-Linolenic acid metabolism | A09100 Metabolism | 0.1175514 | [Solyc01g066620] |
| *SlAlba1* | 00071 Fatty acid degradation | A09100 Metabolism | 0.1318688 | [Solyc01g066620] |
| *SlAlba1* | 04131 Membrane trafficking | A09180 Brite Hierarchies | 0.1635698 | [Solyc01g094400, Solyc01g091300, Solyc01g096410, Solyc01g091020] |
| *SlAlba1* | 04070 Phosphatidylinositol signaling system | A09130 Environmental Information Processing | 0.1757509 | [Solyc01g096410] |
| *SlAlba1* | 00562 Inositol phosphate metabolism | A09100 Metabolism | 0.1780005 | [Solyc01g096410] |
| *SlAlba1* | 04147 Exosome | A09180 Brite Hierarchies | 0.1932533 | [Solyc01g010360, Solyc01g094400, Solyc01g091300] |
| *SlAlba1* | B 09110 Biosynthesis of other secondary metabolites | A09100 Metabolism | 0.2123418 | [Solyc01g009240, Solyc01g010390] |
| *SlAlba1* | 01004 Lipid biosynthesis proteins | A09180 Brite Hierarchies | 0.2174993 | [Solyc01g091560] |
| *SlAlba1* | 00480 Glutathione metabolism | A09100 Metabolism | 0.2612796 | [Solyc01g081250] |
| *SlAlba1* | B 09107 Glycan biosynthesis and metabolism | A09100 Metabolism | 0.2693477 | [Solyc01g005110] |
| *SlAlba1* | B 09191 Unclassified: metabolism | A09190 Not Included in Pathway or Brite | 0.2853175 | [Solyc01g094910, Solyc01g008050] |
| *SlAlba1* | 04812 Cytoskeleton proteins | A09180 Brite Hierarchies | 0.3562572 | [Solyc01g094400] |
| *SlAlba1* | 04144 Endocytosis | A09140 Cellular Processes | 0.3598012 | [Solyc01g096410] |
| *SlAlba1* | A09190 Not Included in Pathway or Brite | A09190 Not Included in Pathway or Brite | 0.4104152 | [Solyc01g094910, Solyc01g008050] |
| *SlAlba1* | 01009 Protein phosphatases and associated proteins | A09180 Brite Hierarchies | 0.5026504 | [Solyc01g096170] |
| *SlAlba1* | 01001 Protein kinases | A09180 Brite Hierarchies | 0.5040355 | [Solyc01g096170] |
| *SlAlba1* | B 09108 Metabolism of cofactors and vitamins | A09100 Metabolism | 0.5335866 | [Solyc01g068630] |
| *SlAlba1* | 03400 DNA repair and recombination proteins | A09180 Brite Hierarchies | 0.5853759 | [Solyc01g010360] |
| *SlAlba1* | 99980 Enzymes with EC numbers | A09190 Not Included in Pathway or Brite | 0.6157035 | [Solyc01g094910] |
| *SlAlba1* | A09140 Cellular Processes | A09140 Cellular Processes | 0.6316198 | [Solyc01g096410] |
| *SlAlba1* | B 09141 Transport and catabolism | A09140 Cellular Processes | 0.6316198 | [Solyc01g096410] |
| *SlAlba1* | 03029 Mitochondrial biogenesis | A09180 Brite Hierarchies | 0.6367832 | [Solyc01g087070] |
| *SlAlba1* | B 09103 Lipid metabolism | A09100 Metabolism | 0.67289 | [Solyc01g066620] |
| *SlAlba1* | B 09132 Signal transduction | A09130 Environmental Information Processing | 0.7178608 | [Solyc01g096410] |
| *SlAlba1* | 03000 Transcription factors | A09180 Brite Hierarchies | 0.7257998 | [Solyc01g094320] SlPLIM2a flower specific gene |
| *SlAlba1* | A09130 Environmental Information Processing | A09130 Environmental Information Processing | 0.7447201 | [Solyc01g096410] |
| *SlAlba1* | B 09123 Folding, sorting and degradation | A09120 Genetic Information Processing | 0.7814045 | [Solyc01g091020] |
| *SlAlba1* | B 09181 Protein families: metabolism | A09180 Brite Hierarchies | 0.9276829 | [Solyc01g096170, Solyc01g091560] |
| *SlAlba1* | A09180 Brite Hierarchies | A09180 Brite Hierarchies | 0.9663864 | [Solyc01g010360, Solyc01g008240, Solyc01g094400, Solyc01g091300, Solyc01g091850, Solyc01g096410, Solyc01g091020, Solyc01g094320, Solyc01g091560, Solyc01g087070, Solyc01g096170, Solyc01g068410, Solyc01g081250, Solyc01g066560] |
| *SlAlba1* | B 09182 Protein families: genetic information processing | A09180 Brite Hierarchies | 0.9898759 | [Solyc01g010360, Solyc01g087070, Solyc01g094400, Solyc01g091300, Solyc01g096410, Solyc01g091020, Solyc01g094320] |
| *SlAlba1* | A09120 Genetic Information Processing | A09120 Genetic Information Processing | 0.990845 | [Solyc01g091020] |
| *SlAlba2* | 00040 Pentose and glucuronate interconversions | A09100 Metabolism | 0.0014094 | [Solyc01g099940, Solyc03g058910, Solyc02g067450] |
| *SlAlba2* | 04626 Plant-pathogen interaction | A09150 Organismal Systems | 0.0073591 | [Solyc04g008000, Solyc02g063340, Solyc03g097100] |
| *SlAlba2* | B 09159 Environmental adaptation | A09150 Organismal Systems | 0.011852 | [Solyc04g008000, Solyc02g063340, Solyc03g097100] calcium ion binding; calmodulin; SlCML27 SlCML8 |
| *SlAlba2* | A09150 Organismal Systems | A09150 Organismal Systems | 0.011852 | [Solyc04g008000, Solyc02g063340, Solyc03g097100] |
| *SlAlba2* | 00564 Glycerophospholipid metabolism | A09100 Metabolism | 0.0158787 | [Solyc04g079880, Solyc03g116620] |
| *SlAlba2* | A09140 Cellular Processes | A09140 Cellular Processes | 0.0312843 | [Solyc03g098500, Solyc02g078190, Solyc03g116620] |
| *SlAlba2* | B 09141 Transport and catabolism | A09140 Cellular Processes | 0.0312843 | [Solyc03g098500, Solyc02g078190, Solyc03g116620] |
| *SlAlba2* | 04144 Endocytosis | A09140 Cellular Processes | 0.0394022 | [Solyc03g098500, Solyc03g116620] |
| *SlAlba2* | B 09103 Lipid metabolism | A09100 Metabolism | 0.041388 | [Solyc04g079880, Solyc02g094040, Solyc03g116620] |
| *SlAlba2* | 00565 Ether lipid metabolism | A09100 Metabolism | 0.0532613 | [Solyc03g116620] |
| *SlAlba2* | 00100 Steroid biosynthesis | A09100 Metabolism | 0.0716251 | [Solyc02g094040] |
| *SlAlba2* | A09100 Metabolism | A09100 Metabolism | 0.117494 | [Solyc04g079880, Solyc01g099940, Solyc02g094040, Solyc03g058910, Solyc02g078190, Solyc02g067450, Solyc03g116620, Solyc02g085660] |
| *SlAlba2* | 00561 Glycerolipid metabolism | A09100 Metabolism | 0.1417706 | [Solyc04g079880] |
| *SlAlba2* | 04145 Phagosome | A09140 Cellular Processes | 0.1485029 | [Solyc02g078190] |
| *SlAlba2* | 02000 Transporters | A09180 Brite Hierarchies | 0.1512258 | [Solyc03g005980, Solyc02g091420, Solyc03g114200] |
| *SlAlba2* | 01004 Lipid biosynthesis proteins | A09180 Brite Hierarchies | 0.1618181 | [Solyc04g079880] |
| *SlAlba2* | 04131 Membrane trafficking | A09180 Brite Hierarchies | 0.1982193 | [Solyc03g098500, Solyc02g094370, Solyc03g116620] |
| *SlAlba2* | B 09101 Carbohydrate metabolism | A09100 Metabolism | 0.1987293 | [Solyc01g099940, Solyc03g058910, Solyc02g067450] |
| *SlAlba2* | 00190 Oxidative phosphorylation | A09100 Metabolism | 0.2874254 | [Solyc02g078190] |
| *SlAlba2* | 00940 Phenylpropanoid biosynthesis | A09100 Metabolism | 0.344657 | [Solyc02g085660] |
| *SlAlba2* | 03021 Transcription machinery | A09180 Brite Hierarchies | 0.3563425 | [Solyc01g108310] |
| *SlAlba2* | 01003 Glycosyltransferases | A09180 Brite Hierarchies | 0.3576286 | [Solyc02g085660] |
| *SlAlba2* | B 09110 Biosynthesis of other secondary metabolites | A09100 Metabolism | 0.4682645 | [Solyc02g085660] |
| *SlAlba2* | B 09102 Energy metabolism | A09100 Metabolism | 0.5704954 | [Solyc02g078190] |
| *SlAlba2* | B 09183 Protein families: signaling and cellular processes | A09180 Brite Hierarchies | 0.5862166 | [Solyc03g005980, Solyc02g091420, Solyc03g114200] SWEET5a |
| *SlAlba2* | 03000 Transcription factors | A09180 Brite Hierarchies | 0.6059362 | [Solyc02g067760] |
| *SlAlba2* | 03036 Chromosome and associated proteins | A09180 Brite Hierarchies | 0.7195953 | [Solyc02g094370] |
| *SlAlba2* | B 09181 Protein families: metabolism | A09180 Brite Hierarchies | 0.8102446 | [Solyc04g079880, Solyc02g085660] |
| *SlAlba2* | A09180 Brite Hierarchies | A09180 Brite Hierarchies | 0.9521377 | [Solyc04g079880, Solyc01g108310, Solyc03g098500, Solyc02g094370, Solyc03g005980, Solyc02g091420, Solyc03g114200, Solyc02g067760, Solyc03g116620, Solyc02g085660] |
| *SlAlba2* | B 09182 Protein families: genetic information processing | A09180 Brite Hierarchies | 0.9801184 | [Solyc01g108310, Solyc03g098500, Solyc02g094370, Solyc02g067760, Solyc03g116620] |
| *SlAlba4* | 03032 DNA replication proteins | A09180 Brite Hierarchies | 1.407E-07 | [Solyc05g014690, Solyc06g008510, Solyc01g088380, Solyc02g093300, Solyc01g111550, Solyc01g079500] |
| *SlAlba4* | 03030 DNA replication | A09120 Genetic Information Processing | 6.337E-05 | [Solyc01g088380, Solyc02g093300, Solyc01g079500] |
| *SlAlba4* | B 09124 Replication and repair | A09120 Genetic Information Processing | 1.72E-04 | [Solyc03g117510, Solyc01g088380, Solyc02g093300, Solyc01g079500] |
| *SlAlba4* | 03400 DNA repair and recombination proteins | A09180 Brite Hierarchies | 2.92E-03 | [Solyc03g117510, Solyc05g014690, Solyc03g007800, Solyc02g094100] SlDEAH14 |
| *SlAlba4* | B 09182 Protein families: genetic information processing | A09180 Brite Hierarchies | 1.17E-02 | [Solyc03g117510, Solyc01g086930, Solyc06g008510, Solyc03g114690, Solyc03g007800, Solyc02g093300, Solyc01g111550, Solyc01g079500, Solyc03g093310, Solyc02g094100, Solyc02g069680, Solyc05g014690, Solyc01g088380, Solyc02g087980] |
| *SlAlba4* | A09180 Brite Hierarchies | A09180 Brite Hierarchies | 0.0177595 | [Solyc03g117510, Solyc01g086930, Solyc06g008510, Solyc03g114690, Solyc03g007800, Solyc02g093300, Solyc01g111550, Solyc01g079500, Solyc03g093310, Solyc02g094100, Solyc02g069680, Solyc05g014690, Solyc01g088380, Solyc03g025470, Solyc03g119490, Solyc03g025230, Solyc02g087980] |
| *SlAlba4* | 03036 Chromosome and associated proteins | A09180 Brite Hierarchies | 0.0303528 | [Solyc06g008510, Solyc03g114690, Solyc02g087980, Solyc02g094100] |
| *SlAlba4* | 03410 Base excision repair | A09120 Genetic Information Processing | 0.0770684 | [Solyc03g117510] |
| *SlAlba4* | 04812 Cytoskeleton proteins | A09180 Brite Hierarchies | 0.2716742 | [Solyc03g025470] |
| *SlAlba4* | 04144 Endocytosis | A09140 Cellular Processes | 0.2745623 | [Solyc02g069680] |
| *SlAlba4* | 04626 Plant-pathogen interaction | A09150 Organismal Systems | 0.3393994 | [Solyc03g119490] |
| *SlAlba4* | A09120 Genetic Information Processing | A09120 Genetic Information Processing | 0.3710518 | [Solyc03g117510, Solyc01g088380, Solyc02g093300, Solyc01g079500] |
| *SlAlba4* | B 09159 Environmental adaptation | A09150 Organismal Systems | 0.3902295 | [Solyc03g119490] |
| *SlAlba4* | A09150 Organismal Systems | A09150 Organismal Systems | 0.3902295 | [Solyc03g119490] |
| *SlAlba4* | 01009 Protein phosphatases and associated proteins | A09180 Brite Hierarchies | 0.3951042 | [Solyc01g079500] |
| *SlAlba4* | 01001 Protein kinases | A09180 Brite Hierarchies | 0.396317 | [Solyc03g119490] |
| *SlAlba4* | 04075 Plant hormone signal transduction | A09130 Environmental Information Processing | 0.4452339 | [Solyc01g111310] salinity induced auxin-related gene LAX2 used for SlAlba6 |
| *SlAlba4* | 03041 Spliceosome | A09180 Brite Hierarchies | 0.4650306 | [Solyc01g086930] |
| *SlAlba4* | A09140 Cellular Processes | A09140 Cellular Processes | 0.512635 | [Solyc02g069680] |
| *SlAlba4* | B 09141 Transport and catabolism | A09140 Cellular Processes | 0.512635 | [Solyc02g069680] |
| *SlAlba4* | B 09183 Protein families: signaling and cellular processes | A09180 Brite Hierarchies | 0.5862166 | [Solyc02g069680, Solyc03g025470, Solyc03g025230] |
| *SlAlba4* | B 09132 Signal transduction | A09130 Environmental Information Processing | 0.5977576 | [Solyc01g111310] |
| *SlAlba4* | 04121 Ubiquitin system | A09180 Brite Hierarchies | 0.6155432 | [Solyc03g093310] |
| *SlAlba4* | A09130 Environmental Information Processing | A09130 Environmental Information Processing | 0.6257011 | [Solyc01g111310] |
| *SlAlba4* | 04147 Exosome | A09180 Brite Hierarchies | 0.6781937 | [Solyc02g069680] |
| *SlAlba4* | 02000 Transporters | A09180 Brite Hierarchies | 0.7579823 | [Solyc03g025230] |
| *SlAlba4* | 04131 Membrane trafficking | A09180 Brite Hierarchies | 0.8026207 | [Solyc02g069680] |
| *SlAlba4* | B 09181 Protein families: metabolism | A09180 Brite Hierarchies | 0.8102446 | [Solyc03g119490, Solyc01g079500] |
| *SlAlba5* | 03110 Chaperones and folding catalysts | A09180 Brite Hierarchies | 8.854E-05 | [Solyc03g115230, Solyc02g077670, Solyc01g090550] HSP101 Chaperone protein dnaJ-B fruit DnaJ-A Solyc01g090550 heat stress |
| *SlAlba5* | 99975 Protein processing | A09190 Not Included in Pathway or Brite | 0.0030303 | [Solyc02g031980] |
| *SlAlba5* | B 09192 Unclassified: genetic information processing | A09190 Not Included in Pathway or Brite | 0.0082091 | [Solyc02g031980] |
| *SlAlba5* | 04141 Protein processing in endoplasmic reticulum | A09120 Genetic Information Processing | 0.0855238 | [Solyc01g090550] |
| *SlAlba5* | 03029 Mitochondrial biogenesis | A09180 Brite Hierarchies | 0.1494335 | [Solyc01g090550] |
| *SlAlba5* | A09190 Not Included in Pathway or Brite | A09190 Not Included in Pathway or Brite | 0.2052012 | [Solyc02g031980] |
| *SlAlba5* | B 09123 Folding, sorting and degradation | A09120 Genetic Information Processing | 0.2157282 | [Solyc01g090550] |
| *SlAlba5* | B 09182 Protein families: genetic information processing | A09180 Brite Hierarchies | 0.2940862 | [Solyc03g115230, Solyc02g077670, Solyc01g090550] HSP101 Chaperone protein dnaJ-B fruit DnaJ-A Solyc01g090550 heat stress |
| *SlAlba5* | A09120 Genetic Information Processing | A09120 Genetic Information Processing | 0.5276394 | [Solyc01g090550] |
| *SlAlba5* | A09180 Brite Hierarchies | A09180 Brite Hierarchies | 0.6706342 | [Solyc03g115230, Solyc02g077670, Solyc01g090550] HSP101 Chaperone protein dnaJ-B fruit DnaJ-A Solyc01g090550 heat stress |
| *SlAlba6* | 03032 DNA replication proteins | A09180 Brite Hierarchies | 1.776E-15 | [Solyc01g087500, Solyc03g115050, Solyc05g008540, Solyc06g008510, Solyc03g098250, Solyc02g093300, Solyc01g111550, Solyc01g079500, Solyc02g070780, Solyc05g014690, Solyc01g110130, Solyc01g088380, Solyc01g020440, Solyc01g103960] |
| *SlAlba6* | 03030 DNA replication | A09120 Genetic Information Processing | 1.694E-11 | [Solyc02g070780, Solyc03g115050, Solyc01g110130, Solyc01g088380, Solyc01g020440, Solyc03g098250, Solyc02g093300, Solyc01g079500] |
| *SlAlba6* | 03036 Chromosome and associated proteins | A09180 Brite Hierarchies | 3.809E-11 | [Solyc01g087500, Solyc02g080390, Solyc06g008510, Solyc03g114690, Solyc03g119220, Solyc03g112550, Solyc02g094520, Solyc02g038690, Solyc02g094100, Solyc02g068580, Solyc02g070780, Solyc01g068280, Solyc01g088390, Solyc01g020440, Solyc03g121580, Solyc02g085390, Solyc01g086690, Solyc02g087880, Solyc02g087980] |
| *SlAlba6* | B 09124 Replication and repair | A09120 Genetic Information Processing | 1.181E-10 | [Solyc02g070780, Solyc03g117510, Solyc03g115050, Solyc01g110130, Solyc01g088380, Solyc01g020440, Solyc01g103960, Solyc03g098250, Solyc02g093300, Solyc01g079500, Solyc01g079520] |
| *SlAlba6* | B 09182 Protein families: genetic information processing | A09180 Brite Hierarchies | 8.983E-10 | [Solyc03g117510, Solyc04g077490, Solyc03g115050, Solyc06g008510, Solyc03g007800, Solyc03g098250, Solyc01g111550, Solyc01g111870, Solyc03g093310, Solyc02g094520, Solyc02g094100, Solyc02g069680, Solyc02g070780, Solyc05g014690, Solyc01g110130, Solyc01g088380, Solyc01g103960, Solyc02g085390, Solyc01g087500, Solyc02g080390, Solyc01g086930, Solyc05g008540, Solyc01g010540, Solyc03g114690, Solyc03g119220, Solyc01g079100, Solyc02g093300, Solyc03g112550, Solyc03g113760, Solyc01g079500, Solyc01g079520, Solyc02g038690, Solyc02g068580, Solyc01g068280, Solyc01g088390, Solyc01g020440, Solyc03g121580, Solyc01g086690, Solyc02g069850, Solyc06g065590, Solyc02g087880, Solyc02g087980] |
| *SlAlba6* | 03400 DNA repair and recombination proteins | A09180 Brite Hierarchies | 2.843E-06 | [Solyc03g117510, Solyc05g014690, Solyc03g115050, Solyc01g020440, Solyc01g103960, Solyc03g007800, Solyc03g098250, Solyc01g111870, Solyc01g079520, Solyc02g094100] |
| *SlAlba6* | A09180 Brite Hierarchies | A09180 Brite Hierarchies | 2.864E-06 | [Solyc04g082840, Solyc03g117510, Solyc04g077490, Solyc03g115050, Solyc06g008510, Solyc03g007800, Solyc03g098250, Solyc01g111550, Solyc01g111870, Solyc03g093310, Solyc02g094520, Solyc02g094100, Solyc02g069680, Solyc02g070780, Solyc05g014690, Solyc01g110130, Solyc01g088380, Solyc01g103960, Solyc02g085390, Solyc01g087500, Solyc02g080390, Solyc01g086930, Solyc05g008540, Solyc01g010540, Solyc03g114690, Solyc03g119220, Solyc01g079100, Solyc02g093300, Solyc03g112550, Solyc03g113760, Solyc01g079500, Solyc01g079520, Solyc02g038690, Solyc02g068580, Solyc01g068280, Solyc01g088390, Solyc01g020440, Solyc03g121580, Solyc03g025470, Solyc03g119490, Solyc01g086690, Solyc02g069850, Solyc06g065590, Solyc02g087880, Solyc02g087980] |
| *SlAlba6* | 03430 Mismatch repair | A09120 Genetic Information Processing | 0.0005192 | [Solyc03g115050, Solyc01g020440, Solyc01g079520] |
| *SlAlba6* | 03420 Nucleotide excision repair | A09120 Genetic Information Processing | 0.0020402 | [Solyc03g115050, Solyc01g020440, Solyc03g098250] |
| *SlAlba6* | 03410 Base excision repair | A09120 Genetic Information Processing | 0.0176094 | [Solyc03g117510, Solyc03g098250] |
| *SlAlba6* | A09120 Genetic Information Processing | A09120 Genetic Information Processing | 0.0182621 | [Solyc03g117510, Solyc03g115050, Solyc01g010540, Solyc03g098250, Solyc02g093300, Solyc01g079500, Solyc01g079520, Solyc02g070780, Solyc01g110130, Solyc01g088380, Solyc01g020440, Solyc01g103960, Solyc02g069850, Solyc06g065590] |
| *SlAlba6* | 03440 Homologous recombination | A09120 Genetic Information Processing | 0.0284991 | [Solyc03g115050, Solyc01g103960] |
| *SlAlba6* | 00310 Lysine degradation | A09100 Metabolism | 0.1263486 | [Solyc02g094520] |
| *SlAlba6* | 01001 Protein kinases | A09180 Brite Hierarchies | 0.1334187 | [Solyc04g082840, Solyc03g119490, Solyc01g111870] |
| *SlAlba6* | 04812 Cytoskeleton proteins | A09180 Brite Hierarchies | 0.191198 | [Solyc03g025470, Solyc02g087880] |
| *SlAlba6* | 03010 Ribosome | A09120 Genetic Information Processing | 0.2461736 | [Solyc01g010540, Solyc02g069850, Solyc06g065590] |
| *SlAlba6* | 03011 Ribosome | A09180 Brite Hierarchies | 0.2718919 | [Solyc01g010540, Solyc02g069850, Solyc06g065590] |
| *SlAlba6* | 04145 Phagosome | A09140 Cellular Processes | 0.3373161 | [Solyc02g087880] |
| *SlAlba6* | 01009 Protein phosphatases and associated proteins | A09180 Brite Hierarchies | 0.3625192 | [Solyc03g119220, Solyc01g079500] |
| *SlAlba6* | 03000 Transcription factors | A09180 Brite Hierarchies | 0.4108401 | [Solyc04g077490, Solyc03g115050, Solyc03g113760] floral regulator |
| *SlAlba6* | 04147 Exosome | A09180 Brite Hierarchies | 0.5383659 | [Solyc02g069680, Solyc02g087880, Solyc02g038690] |
| *SlAlba6* | A09140 Cellular Processes | A09140 Cellular Processes | 0.54217 | [Solyc02g069680, Solyc02g087880] |
| *SlAlba6* | B 09141 Transport and catabolism | A09140 Cellular Processes | 0.54217 | [Solyc02g069680, Solyc02g087880] |
| *SlAlba6* | 04144 Endocytosis | A09140 Cellular Processes | 0.560247 | [Solyc02g069680] |
| *SlAlba6* | 04626 Plant-pathogen interaction | A09150 Organismal Systems | 0.6539526 | [Solyc03g119490] |
| *SlAlba6* | B 09122 Translation | A09120 Genetic Information Processing | 0.6961318 | [Solyc01g010540, Solyc02g069850, Solyc06g065590] |
| *SlAlba6* | B 09159 Environmental adaptation | A09150 Organismal Systems | 0.7180751 | [Solyc03g119490] |
| *SlAlba6* | A09150 Organismal Systems | A09150 Organismal Systems | 0.7180751 | [Solyc03g119490] |
| *SlAlba6* | 04075 Plant hormone signal transduction | A09130 Environmental Information Processing | 0.7786666 | [Solyc01g111310] LAX2 |
| *SlAlba6* | 03041 Spliceosome | A09180 Brite Hierarchies | 0.7983241 | [Solyc01g086930] |
| *SlAlba6* | B 09181 Protein families: metabolism | A09180 Brite Hierarchies | 0.8822479 | [Solyc04g082840, Solyc03g119220, Solyc03g119490, Solyc01g079500, Solyc01g111870] |
| *SlAlba6* | 03019 Messenger RNA biogenesis | A09180 Brite Hierarchies | 0.8862324 | [Solyc02g087880] |
| *SlAlba6* | B 09132 Signal transduction | A09130 Environmental Information Processing | 0.9027995 | [Solyc01g111310] |
| *SlAlba6* | B 09105 Amino acid metabolism | A09100 Metabolism | 0.9063114 | [Solyc02g094520] |
| *SlAlba6* | 04121 Ubiquitin system | A09180 Brite Hierarchies | 0.9134243 | [Solyc03g093310] |
| *SlAlba6* | 04131 Membrane trafficking | A09180 Brite Hierarchies | 0.9158106 | [Solyc02g069680, Solyc01g079100] |
| *SlAlba6* | A09130 Environmental Information Processing | A09130 Environmental Information Processing | 0.9191593 | [Solyc01g111310] salinity induced auxin-related gene LAX2 |
| *SlAlba6* | B 09183 Protein families: signaling and cellular processes | A09180 Brite Hierarchies | 0.9571513 | [Solyc02g069680, Solyc03g025470, Solyc02g087880, Solyc02g038690] |
| *SlAlba6* | A09100 Metabolism | A09100 Metabolism | 0.9999999 | [Solyc02g094520] |
| *SlAlba7* | 03110 Chaperones and folding catalysts | A09180 Brite Hierarchies | 0.0075831 | [Solyc02g061950, Solyc08g062700] |
| SlAlba7 | 03041 Spliceosome | A09180 Brite Hierarchies | 0.0108412 | [Solyc08g062700, Solyc07g017490] |
| SlAlba7 | 04130 SNARE interactions in vesicular transport | A09120 Genetic Information Processing | 0.0204184 | [Solyc07g065030] salt induced SlSYP51.2 Solyc07g065030 development |
| SlAlba7 | 04070 Phosphatidylinositol signaling system | A09130 Environmental Information Processing | 0.0378784 | [Solyc01g107750] Tryosin phospatase |
| SlAlba7 | 00562 Inositol phosphate metabolism | A09100 Metabolism | 0.0384036 | [Solyc01g107750] |
| SlAlba7 | 01009 Protein phosphatases and associated proteins | A09180 Brite Hierarchies | 0.1302408 | [Solyc01g107750] |
| SlAlba7 | B 09182 Protein families: genetic information processing | A09180 Brite Hierarchies | 0.1723121 | [Solyc02g061950, Solyc08g062700, Solyc07g017490, Solyc07g065030] |
| SlAlba7 | A09180 Brite Hierarchies | A09180 Brite Hierarchies | 0.1813071 | [Solyc01g107750, Solyc02g061950, Solyc08g062700, Solyc07g017490, Solyc07g065030] |
| SlAlba7 | B 09132 Signal transduction | A09130 Environmental Information Processing | 0.2233686 | [Solyc01g107750] |
| SlAlba7 | A09130 Environmental Information Processing | A09130 Environmental Information Processing | 0.2387357 | [Solyc01g107750] |
| SlAlba7 | B 09123 Folding, sorting and degradation | A09120 Genetic Information Processing | 0.2619668 | [Solyc07g065030] |
| SlAlba7 | 04131 Membrane trafficking | A09180 Brite Hierarchies | 0.3626254 | [Solyc07g065030] |
| SlAlba7 | B 09101 Carbohydrate metabolism | A09100 Metabolism | 0.3630032 | [Solyc01g107750] |
| SlAlba7 | B 09181 Protein families: metabolism | A09180 Brite Hierarchies | 0.5835008 | [Solyc01g107750] Tyrosine phosphatase cellular process |
| SlAlba7 | A09120 Genetic Information Processing | A09120 Genetic Information Processing | 0.6084225 | [Solyc07g065030] |
| SlAlba7 | A09100 Metabolism | A09100 Metabolism | 0.8181393 | [Solyc01g107750] |
